# Topology optimization of random memristors for input-aware dynamic SNN

**DOI:** 10.1126/sciadv.ads5340

**Published:** 2025-04-16

**Authors:** Bo Wang, Xinyuan Zhang, Shaocong Wang, Ning Lin, Yi Li, Yifei Yu, Yue Zhang, Jichang Yang, Xiaoshan Wu, Yangu He, Songqi Wang, Tao Wan, Rui Chen, Guoqi Li, Yue Deng, Xiaojuan Qi, Zhongrui Wang, Dashan Shang

**Affiliations:** ^1^ Department of Electrical and Electronic Engineering, The University of Hong Kong, Hong Kong, China.; ^2^ ACCESS – AI Chip Center for Emerging Smart Systems, InnoHK Centers, Hong Kong Science Park, Hong Kong, China.; ^3^ State Key Lab of Fabrication Technologies for Integrated Circuits, Institute of Microelectronics, Chinese Academy of Sciences, Beijing 100029, China.; ^4^ Laboratory of Microelectronic Devices & Integrated Technology, Institute of Microelectronics, Chinese Academy of Sciences, Beijing 100029, China.; ^5^ University of Chinese Academy of Sciences, Beijing 100049, China.; ^6^ Key Laboratory of Brain Cognition and Brain-inspired Intelligence Technology, Institute of Automation, Chinese Academy of Sciences, Beijing 100190, China.; ^7^ School of Artificial Intelligence, Beihang University, Beijing 100191, China.; ^8^ School of Astronautics, Beihang University, Beijing 100191, China.

## Abstract

Machine learning has advanced unprecedentedly, exemplified by GPT-4 and SORA. However, they cannot parallel human brains in efficiency and adaptability due to differences in signal representation, optimization, runtime reconfigurability, and hardware architecture. To address these challenges, we introduce pruning optimization for input-aware dynamic memristive spiking neural network (PRIME). PRIME uses spiking neurons to emulate brain’s spiking mechanisms and optimizes the topology of random memristive SNNs inspired by structural plasticity, effectively mitigating memristor programming stochasticity. It also uses the input-aware early-stop policy to reduce latency and leverages memristive in-memory computing to mitigate von Neumann bottleneck. Validated on a 40-nm, 256-K memristor-based macro, PRIME achieves comparable classification accuracy and inception score to software baselines, with energy efficiency improvements of 37.8× and 62.5×. In addition, it reduces computational loads by 77 and 12.5% with minimal performance degradation and demonstrates robustness to stochastic memristor noise. PRIME paves the way for brain-inspired neuromorphic computing.

## Teaser

PRIME optimizes memristive SNNs with pruning and input-aware early stop, enhancing energy efficiency for neuromorphic hardware.

## INTRODUCTION

Machine learning with artificial neural networks (ANNs) has undergone substantial advancements in recent years (*1*–*3*), as demonstrated by the development of sophisticated large language models (*4*, *5*) such as GPT-4 and advanced world simulators (*6*) like SORA. These models, operating on digital computers, exhibit human-like capabilities and are steps toward the long-term goal of artificial general intelligence.

Despite these achievements, a discernible discrepancy in performance persists compared to the human brain, especially in terms of energy efficiency (*7*–*11*) and adapting to inputs of different difficulties by dynamically allocating computing resources (*12*–*16*). This gap can be attributed to fundamental differences in signal representation, optimization, runtime reconfigurability, and architecture.

**Fig. 1.**
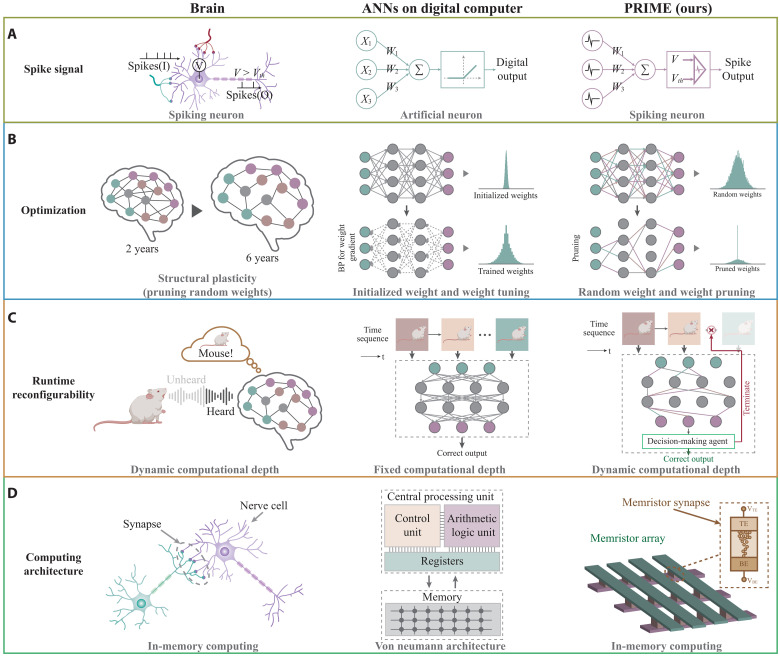
Brain-inspired topology optimization for input-aware dynamic SNN on memristors. (**A**) Comparison of the information representation in human brain, the artificial neuron model of conventional static ANNs, and the spiking neuron model of PRIME. Both the human brain and PRIME encode information with spikes, whereas ANNs do not. (**B**) Comparison of the optimization scheme in human brain with structural plasticity, weight-trained neural network, and topology-optimized neural network. The human brain and PRIME optimize network topology instead of relying on fine-tuning of synaptic weights, as seen in conventional ANNs. (**C**) Comparison of runtime reconfigurability in human brain with dynamic computational depth, conventional static ANNs, and PRIME. The human brain and PRIME feature dynamic computational depth and dynamically adapt to stimuli for reducing computational costs. In contrast, conventional ANNs are of fixed computational depth that is constant to inputs of different difficulties. (**D**) Comparison of the hardware architecture in human brain, the digital hardware implementing conventional ANNs, and memristive neuromorphic system on PRIME. The human brain and PRIME use in-memory computing, which collocates processing and memory in biological synapses and memristors, respectively, thereby enhancing energy efficiency. In contrast, digital computers based on the von Neumann architecture, separate storage and computing.

In terms of signal representation, the human brain uses spikes (*17*, *18*) for information representation (Fig. 1A, left). These spikes are sparse, robust to signal noise, and enable the brain to perform advanced cognitive tasks at a minimal energy of only 20 watts (*19*, *20*). In contrast, mainstream artificial intelligence (AI) systems use digital-valued computation instead of the spiking mechanisms (Fig. 1A, middle).

In terms of optimization, a major step in brain’s development is initially characterized by an abundance of random synaptic connections. This configuration undergoes structural plasticity (*21*–*23*), a process that entails the elimination of less important connections while retaining informative ones (Fig. 1B, left). In contrast, current AI predominantly concentrates on the meticulous tuning of synaptic weights (Fig. 1B, middle) that does not work well on emerging synaptic devices.

In terms of runtime reconfigurability, the brain dynamically reallocates its computational resources by adjusting the depth of computation in sequential decision-making tasks according to task demands (*12*–*16*). When solving complex tasks, the human brain estimates future consequences by integrating past experiences (*13*–*15*), including accumulated scores (*14*), the sequential interdependence of previous inputs (*15*), etc. (*13*), to determine the appropriate termination point for making a final decision. This dynamic computational depth enables the brain to balance efficiency and accuracy within the constraints of limited computational budget (*12*, *16*). Nevertheless, major conventional ANNs remain static, which works on entire inputs before decision making (Fig. 1C, middle).

In terms of hardware architecture, nerve cells communicate with each other through synaptic connections (Fig. 1D, left). The latter both store information as synaptic strength and perform computation (i.e., modulating signal transmission) right at where the information is stored (*24*, *25*). This mechanism is highly energy efficient as no data movement is needed, while being parallel. Conversely, mainstream AI hardware relies on the digital von Neumann architecture (*26*–*28*), which physically separates memory from computing, incurring large energy and time consumption due to massive data shuttling. To address these differences, we proposed pruning optimization for input-aware dynamic memristive spiking neural network (PRIME).

In terms of signal representation, PRIME is a spiking neural network (SNN) with brain-inspired leaky integrate-and-fire (LIF) neurons to process and propagate information (Fig. 1A, right). This spiking mechanism enhances energy efficiency with emerging neuromorphic hardware.

In terms of optimization, PRIME implements structural plasticity in a manner akin to human brain. According to strong lottery ticket hypothesis theory (*29*, *30*), a subnet sampled from a supernet with sufficient random weights can achieve competitive accuracy relative to the target network with well-optimized weights. Here, PRIME leverages the inherent programming stochasticity of memristive synapses for initiating random weights, followed by topology optimization (Fig. 1B, right) to preserve informative synapses while eliminating those considered less critical. This topology optimization approach naturally avoids the energy-intensive and time-consuming memristor conductance tuning.

In terms of runtime reconfigurability, inspired by the dynamic computational depth of human brain, PRIME implements the input-aware dynamic latency for SNN during the inference phase. Since the latency (represented by the number of timesteps) in SNN profoundly influences performance and directly correlates with energy consumption in hardware (*31*–*33*), we introduce an agent to compute time-wise SNN output confidence, which dynamically adjusts timesteps for each input sample (Fig. 1C, right). This strategy adapts to inputs of different difficulties at minimal accuracy loss and computational load.

In terms of hardware architecture, PRIME uses neuromorphic in-memory computing paradigm analogous to the brain, using memristor synapses to store synaptic weights and modulate signal transmission between spiking neurons (Fig. 1D, right). The integration of memory and computing in neuromorphic hardware substantially enhances parallelism and reduces energy consumption compared with the traditional von Neumann architecture (Fig. 1D, middle). In addition, we exploit the inherent electroforming stochasticity of memristors to produce large-scale and cost-effective true random weights, turning it into an advantage through our topology optimization strategy.

Our research validates this system on two tasks: event image classification and image inpainting, executed on a hybrid analog-digital system with a 40-nm, 256-K memristor-based in-memory computing core. In the N-MNIST classification task, PRIME achieves comparable accuracy to software baseline but with 37.83× improvements in energy efficiency and 67.6% reduction in calculation cost. For image inpainting, our system parallels the reconstruction loss and inception score (IS) (*34*) of software baseline, showcasing its ability to generate diverse and high-quality images. In addition, it achieves approximately 62.50× reductions in energy consumption compared to software baseline on conventional digital hardware and 12.5% computational load savings with minimal performance degradation. Furthermore, PRIME is robust to synaptic noise. PRIME paves the way for future brain-inspired, energy-efficient, and low-latency neuromorphic computing paradigm.

## RESULTS

### PRIME: Topology pruning optimization for input-aware dynamic memristive SNN

**Fig. 2.**
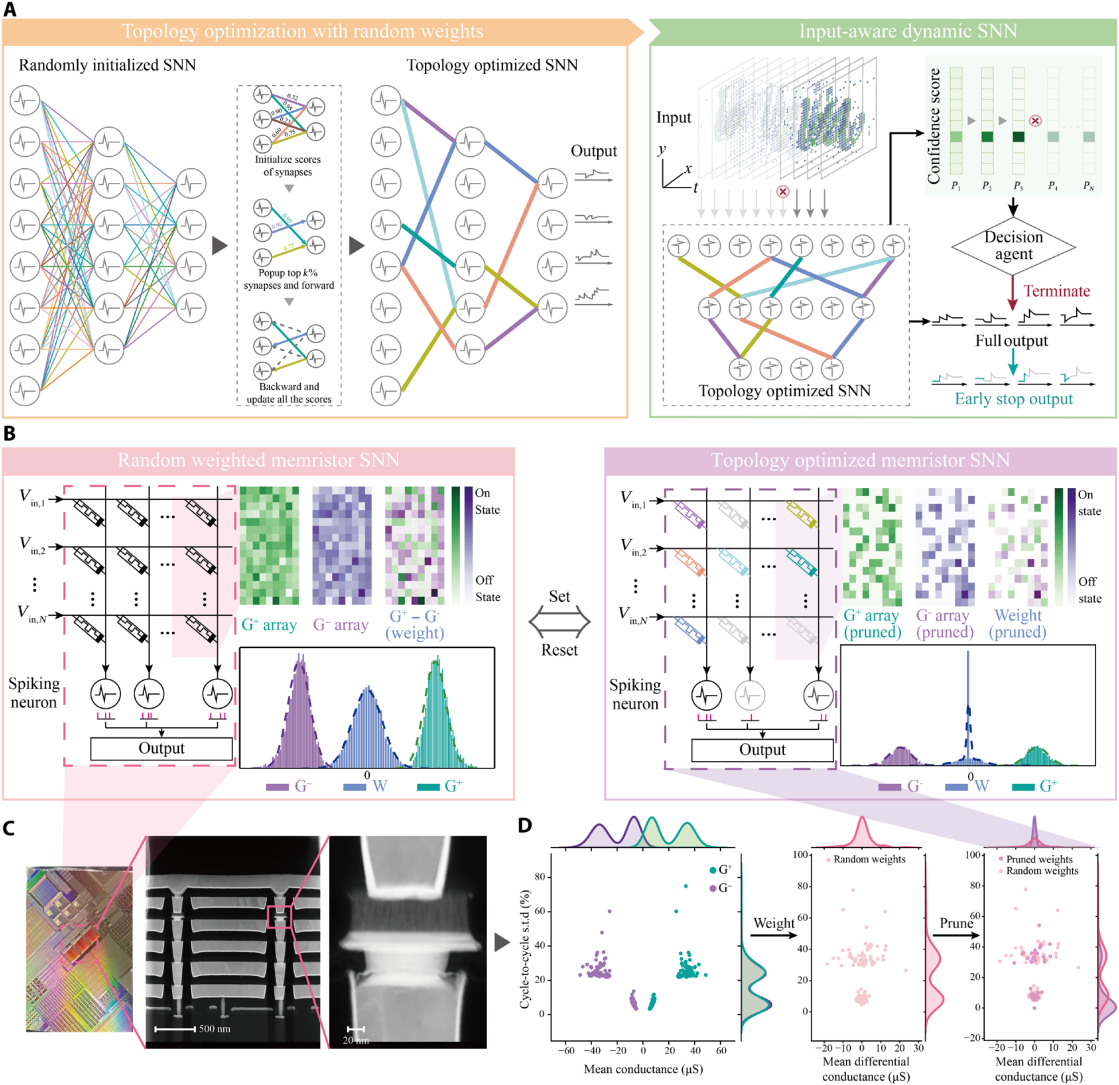
Overview of PRIME. (**A**) The brain-inspired topology optimization of randomly initialized SNN (left). Initially, an overparameterized SNN with random memristor connections is generated using inherit programming stochasticity. Each random synaptic weight is then assigned a score *s*, reflecting its importance. Synapses with the top *k*% scores are retained, while others are pruned to form a subnet. The loss is calculated using this subnet and backpropagated through the entire network to optimize the scores. This process is iterated until convergence, yielding the optimal subnet. Further details are provided in Materials and Methods. The brain-inspired input-aware dynamic SNN in inference (right). In inference, time-wise confidence is calculated, either as a softmax score or a consistency score, depending on the task. If the confidence meets the threshold policy, the inference terminates. Further details are provided in Materials and Methods. (**B**) The schematic of memristor-based SNN before (left) and after (right) topology optimization, consisting of memristor crossbar arrays and LIF neurons. Before pruning, the differential memristor pairs in the randomly overparameterized supernet (**G**^+^ and **G**^−^) follows a mixture of Gaussian distributions. After pruning, the redundant memristor pairs are RESET, resulting in a conductance peak around zero. (**C**) Optical photo of the 40-nm, 256-K memristor in-memory computing macro (left). Cross-sectional high-angle annular dark-field imaging–scanning transmission electron microscopy image of the memristor array (middle and right). (**D**) Joint distribution of the mean conductance and SD of 128 randomly selected resistive memory cells in 10,000 reinstating programming cycles (left). Joint distribution of the 128 resistive differential pairs before (middle) and after pruning (right).

PRIME is systematically illustrated in Fig. 2. Figure 2A (left) illustrates the pruning optimization inspired by human brain’s structural synaptic plasticity (*21*, *22*), which contributes to the maturation of human brain by preserving the functional synapses while eliminating redundant ones (Fig. 1B, left). In the training phase, random synaptic weights are generated by the inherent programming stochasticity of memristors (Fig. 2A, left). Each synapse has a pop-up score *s*, indicative of its significance. During the forward pass, the synapses with top *k*% pop-up scores are preserved while others are pruned. Subsequently, in the backward pass, these scores undergo gradient-based updates aimed at minimizing the training loss, thereby optimizing the topology of SNN (see Materials and Methods). This topology optimization gets rid of the expensive memristor conductance fine-tuning (figs. S1 to S3; see note S1 for details about memristor conductance fine-tuning) and transforms the memristor programming stochasticity into an advantage (see note S2 for a more detailed theoretical proof of topology optimization for memristor-based SNN).

In Fig. 2A (right), drawing inspiration from the human brain’s dynamic computational depth (*12*–*16*), which enhances the brain’s ability to rapidly and efficiently recognize and adapt to stimuli, we implement the input-aware dynamic early stop policy in the pruned memristive SNN during the inference phase. The input spikes to SNN are temporally spanned in a time window. As such, increase in SNN timesteps increases the network performance, a common observation in computing with event data (*31*–*33*). Here we introduce the confidence thresholding, where an agent computes the confidence scores of SNN outputs over different timesteps. If the confidence score meets the predefined threshold, the inference process terminates (see Materials and Methods for details). This approach dynamically adjusts the computational load of each sample, aiming to reduce both latency and energy consumption, with minimal impact on the model’s overall performance.

Hardware-wise, the memristor array is divided into positive and negative sub-arrays (**G**^+^ and **G**^−^), representing the weight matrix through the conductance difference (Fig. 2B, left). Initially, these memristors are insulating, leading to a weight distribution centered around zero conductance. After electroforming, these arrays exhibit random and analog conductance, this translates to neural network weights following a mixture of two quasi-normal distributions due to the stochasticity in memristor programming. These random weights constitute the overparameterized supernet. After training on computers, the optimized subnet is acquired by pruning unnecessary connections, which is physically done by RESET the corresponding memristor pairs (fig. S4). The conductance distribution in the optimized subnet shows a peak around zero conductance due to the pruned synapses (Fig. 2B, right). The integrated memristor arrays are the analog cores of a hybrid analog-digital computing system (Fig. 2C, figs. S5 to S9, and note S3), which implements vector-matrix multiplications. The digital core carries out nonmatrix operations, including LIF neurons (figs. S6 and S7; see note S3 for details about LIF neuron circuit). The conductance distributions of selected memristors are presented in Fig. 2D, illustrating changes in the means and standard deviations of synaptic weights as pruning occurs.

### Image classification for neuromorphic dataset using PRIME

**Fig. 3.**
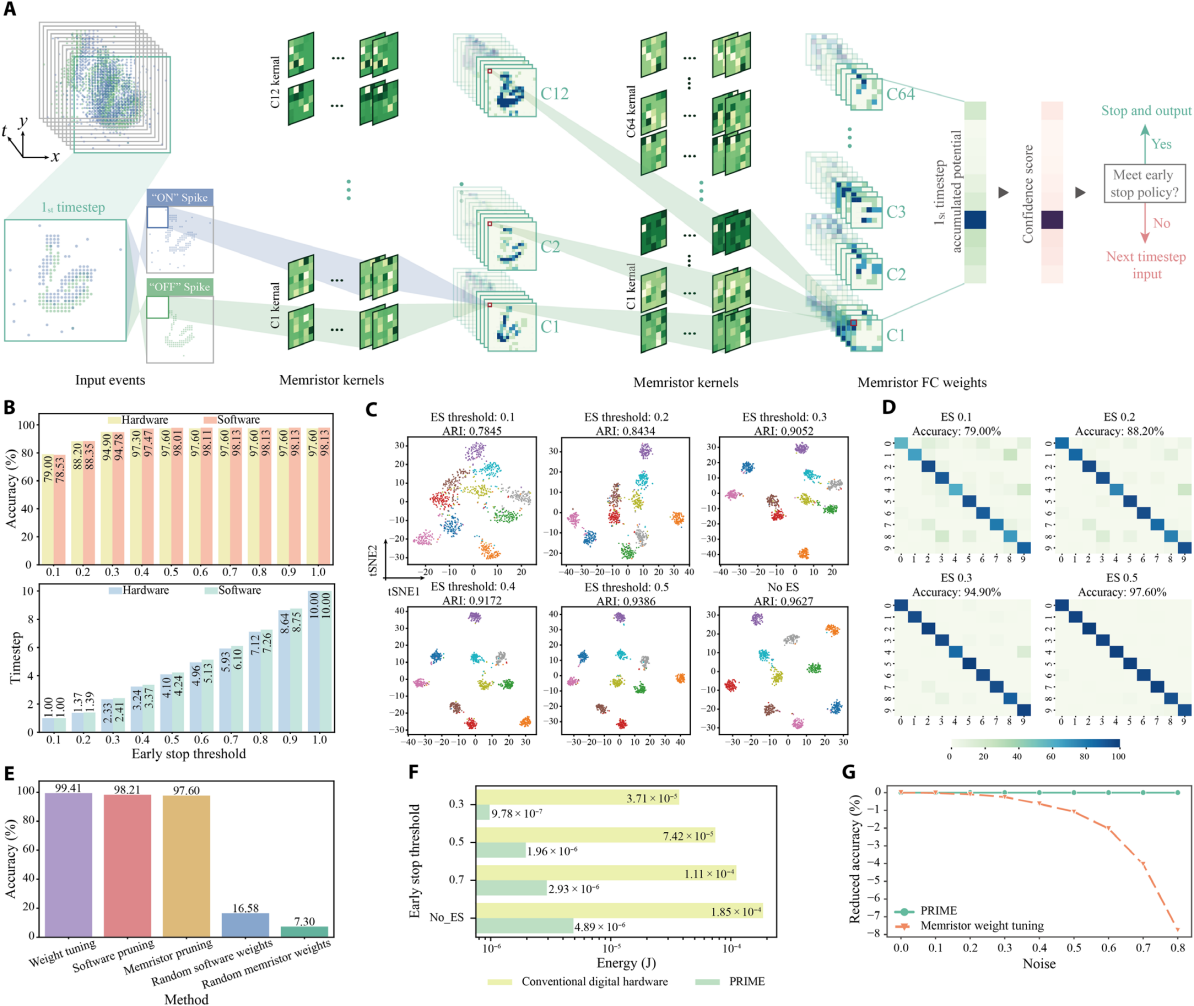
Experimental image classification for N-MNIST dataset with PRIME. (**A**) Illustration of the convolutional SNN of PRIME during the inference, showing pruned random memristor kernels and associated feature maps in the N-MNIST classification. The network outputs at each timestep are used to compute confidence scores. (**B**) The classification accuracy and dynamic latency (evaluated as the average timesteps of the test data) comparisons of hardware PRIME and software baseline at various early stop thresholds. (**C**) tSNE visualizations of feature maps from PRIME at different early stop thresholds, colored according to ground truth labels. (**D**) Confusion matrices and classification accuracy of PRIME at different early stop thresholds. (**E**) The classification accuracy for SNNs by different optimization methods. Weight tuning: Optimize the weights of software SNN through STBP. Software pruning: Optimize the topology of software SNN, with randomly initialized weights. Memristor pruning: Optimize the topology of memristor-based SNN, where the random weights are produced by memristor programming stochasticity. Random software and memristor weights: The SNNs are initialized with random weights, separately implemented in software and on memristors. (**F**) Comparison of the inference energy of a single image on a projected hybrid analog-digital system and digital hardware at different early stop thresholds. The former shows a substantial energy reduction due to in-memory computing. (**G**) Impact of memristor programming noise on accuracy between memristor-based SNNs optimized by different methods at various noise levels.

We first validate PRIME on classifying the representative N-MNIST dataset using a three-layer spiking convolutional neural network (Fig. 3A).

N-MNIST (*35*) is a neuromorphic image datasets of 10 digits captured by dynamic vision sensors (DVSs). Each sample, comprising “on” and “off” spike streams within a 34 × 34 pixel frame spanning 300 ms, is processed into 10 time bins using SpikingJelly (*36*) (Fig. 3A, figs. S10 and S11, and table S1; see note S4 for discussion about the spike stream preprocessing). Here, we use a supernet consisting of two convolution layers and a linear classification layer, with all synaptic weights initially mapped to random memristor conductance differentials. The softmax-based confidence score (Fig. 3A; see Materials and Methods) dynamically adjusts the inference timestep for each input sample, optimizing processing efficiency.

To assess PRIME’s performance in terms of classification accuracy and inference efficiency, we first conduct a comparative analysis between the software baseline (software) and PRIME (hardware) across various early stop thresholds (Fig. 3B). The average timesteps in the test dataset are used as evaluation metric. PRIME closely matches the accuracy of the software baseline at timestep 10 for N-MNIST classification. Moreover, PRIME achieves substantial latency reductions while maintaining high accuracy, thanks to the dynamic early stop using thresholding method. For example, at a threshold of 0.5, PRIME attains 97.60% accuracy with about 59% reduction in calculation cost (the average timesteps), and at a threshold of 0.3, it achieves 94.9%accuracy with about a 77% reduction in calculation cost. We then visualize the embedded features for the classification head under different early stop thresholds of PRIME (Fig. 3C and fig. S12A) and software counterpart (fig. S12B) using t-distributed stochastic neighbor embedding (tSNE) (*37*). The tSNE visualization results suggest that PRIME representations maintain a discernable subpopulation structure of 10 clusters at the proper early stop threshold (Fig. 3C). The confusion matrices (Fig. 3D and fig. S13) of PRIME is dominated by the diagonal elements at the proper threshold (e.g., 0.5) and hence corroborates the high classification accuracy with a substantial reduction in timesteps.

We compare PRIME with other optimization methods (Fig. 3E) and SNN pruning methods (tables S2 and S3; see note S5 for more details). PRIME parallels the performance of weight-optimized SNN, while eliminating the need for weight fine-tuning. In addition, compared to memristor-initialized SNNs (fixed random weights without any further optimization), PRIME shows notable performance improvement, consistent with software-pruned SNNs. This reveals that the memristor programming stochasticity is well-suited for generating random weights for topology optimization. The performance gap between software-pruning and software-initialized networks and that between memristor-pruning and memristor-initialized networks are similar, proving again that our proposed pruning algorithm is particularly effective for memristor-based networks.

In addition, we present a comparative analysis of energy consumption for a single image classification between a projected hybrid analog-digital system and state-of-the-art digital system including RTX4090 graphic processing unit (GPU) (Fig. 3F) (see fig. S14A and table S8 for comparison with other digital systems). The bottom panel (no early stop) illustrates that our PRIME can markedly decrease energy consumption, by approximately 37.83 times compared to the digital hardware, due to memristive in-memory computing. Moreover, the input-aware dynamic early stop further reduces energy consumption as the early stop threshold decreases (see fig. S15A for detailed energy breakdown). This corroborates the superior energy efficiency of memristor in-memory computing.

We also compare PRIME with conventional memristor weights fine-tuning for network inference under different memristor programming noise (*38*–*40*). For conventional weight fine-tuning, the inevitable programming stochasticity degrades the precision in mapping weights to memristor conductance, thus degrading the network inference performance as the noise increases. In contrast, PRIME leverages this programming stochasticity for weight initialization and its performance is not affected by the noise level.

We further evaluate PRIME using the DVS128 Gesture (*41*) dataset on spiking VGG-11 with simulated memristor conductance differentials (fig. S16; Materials and Methods). The results indicate that PRIME achieves comparable accuracy with weight tuning and software pruning (fig. S16C), while drastically reduces computational cost by 87.15% with only a 1.74% decrease in accuracy (fig. S16, D and E). In addition, energy consumption is drastically reduced by 238.74 times compared to the RTX 4090 GPU (fig. S16F). This simulated experiment shows that PRIME exhibits excellent scalability on larger memristor-based neural networks and promises substantial energy efficiency gains on deeper neural networks.

### Image inpainting using PRIME

Despite neuromorphic image classification, we extended our validation to more complex tasks such as image inpainting, using a spiking variational autoencoder [spiking-VAE (*42*); figs. S17 to S19; see note S6 for more details about spiking-VAE]. Image inpainting, the task of completing missing regions in images, typically uses generation models like VAEs due to their ability to come up with multiple perceptual outcomes (*43*, *44*).

**Fig. 4.**
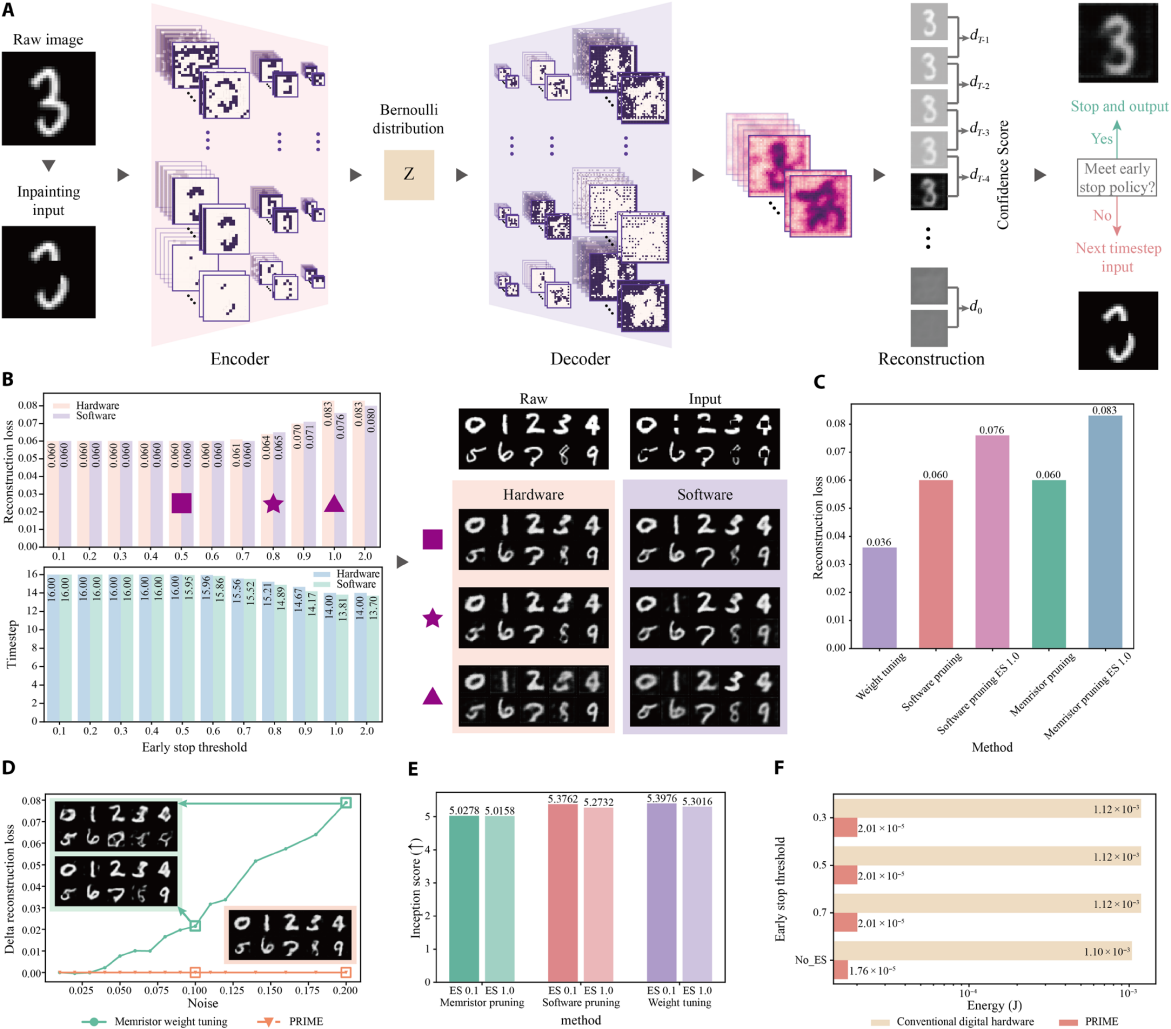
Experimental image inpainting of MNIST dataset with PRIME. (**A**) The spiking VAE of PRIME showing example feature maps during MNIST image inpainting. The latency of spiking VAE is dynamically adapted by the image consistence confidence score (Materials and Methods). (**B**) The reconstruction loss (Materials and Methods) and dynamic timestep (evaluated as the average timesteps of the test data) comparisons of hardware PRIME and software baseline at various early stop thresholds on MNIST (left). The raw, input, and reconstructed images at different thresholds (right). (**C**) The reconstruction loss for SNNs optimized by different methods. Weight tuning: Optimize the weights of software SNN through STBP. Software pruning and software pruning ES 1.0: Optimize the topology of software SNN with randomly initialized weights. The dynamic early stop policy is either applied (former) or not applied (latter) in inference. Memristor pruning and memristor pruning ES 1.0: Optimize the topology of memristor-based SNN, where the random weights are produced by memristor programming stochasticity. The dynamic early stop policy is either applied (former) or not applied (latter) in inference. (**D**) Impact of memristor programming noise on SNNs optimized by different methods at various noise levels. (**E**) The IS comparisons of SNNs by different optimization methods at various early stop thresholds. (**F**) Comparison of the inference energy of a projected hybrid analog-digital system and digital hardware at different early stop thresholds.

Here, we use the representative MNIST (*45*) dataset. We purposely remove the central region of each MNIST image (Fig. 4A; see Materials and Methods). The PRIME uses a spiking-VAE, incorporating encoder, decoder layers, and the Bernoulli sampling layer. The synaptic weights within encoder and decoder layers are first mapped to random memristor conductance differentials before pruning optimization (fig. S20 and table S4; see note S6 for details about the mapping). The confidence score measures the consistency of decoder output over consecutive frames, which dynamically regulates the inference timesteps for each image reconstruction, thereby enhancing energy efficiency and improving inference speed.

We first compare the experimental PRIME with software one across varying early stop thresholds (Fig. 4B). The results, displayed in the left of Fig. 4B, indicate that experimental PRIME shows consistent performance with software one in terms of reconstruction accuracy at different thresholds. Notably, as the early stop threshold increases, there is an approximately 12.5% reduction in latency with minimal impact on reconstruction quality. The experimentally reconstructed images at different thresholds are shown in the right of Fig. 4B, which can hardly be differentiated from those from MNIST dataset.

We compare PRIME with other optimization methods (Fig. 4C). The pruning optimization shows a slightly increased reconstruction loss over weight optimization, while weight optimization is more notably limited by memristor programming noise (Fig. 4D). In addition, early stop leads to a slightly higher reconstruction loss while saving inference computational load.

The IS (*46*) is a widely used metric for generative models. IS assesses the quality and diversity of generated images using a pretrained classifier. A higher IS indicates that the generated images are both high-quality and diverse. The results illustrated in Fig. 4E demonstrate that PRIME is capable of achieving IS comparable to the weight optimized models on software.

Figure 4F shows the estimated energy consumption for a single image reconstruction (see figs. S14B and S15B for comparison with other digital systems). The findings indicate that PRIME substantially reduces energy consumption by a factor of 62.5, compared to SNNs implemented on mainstream digital hardware, which is particularly advantageous for edge AI.

We then evaluate PRIME using the Fashion-MNIST (*47*) dataset on larger neural networks with simulated memristor conductance differentials (fig. S21; Materials and Methods). The results show that PRIME achieves comparable reconstruction accuracy and produces similar reconstructed images to weight-tuning models (fig. S21B). In addition, the early stop policy enables PRIME to reduce computational cost by 8.46% with minimal loss (fig. S21, C and D). Moreover, energy consumption is drastically reduced by 102.82 times compared to the RTX 4090 GPU (fig. S21E). This simulated experiment further demonstrates that PRIME has excellent scalability on larger memristor-based neural networks and can effectively process more complex datasets.

### Impact of memristor read noise

**Fig. 5.**
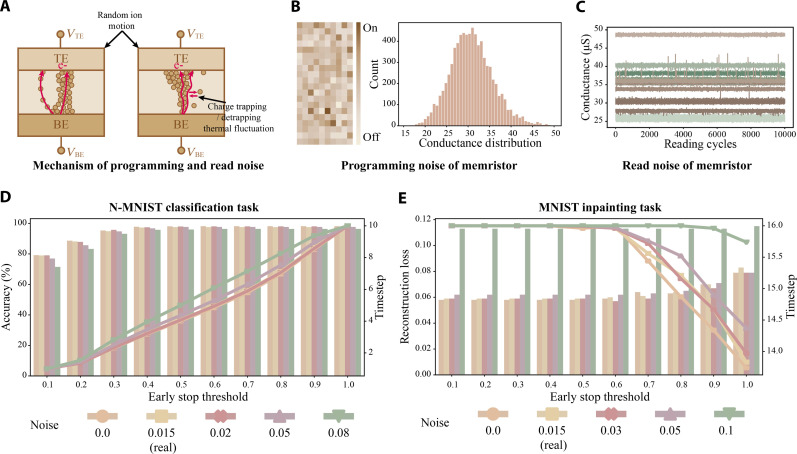
Memristor noise and impact on PRIME. (**A**) The mechanism of memristor programming and read noise. The random ion motion in filament formation and dissolution leads to programming noise, while charge trapping/detrapping and thermal fluctuation yields read noise. (**B**) The memristor programming noise represented as the heatmap and histogram, which follows a quasi-normal distribution for initializing random weights in PRIME. (**C**) The memristor read noise of 15 randomly selected memristors with 10,000 read cycles, showing clear conductance temporal fluctuation that degrades model’s performance. (**D**) Noise robustness evaluation of PRIME at various early stop thresholds on N-MNIST classification. (**E**) Noise robustness evaluation of PRIME at various early stop thresholds on MNIST image inpainting.

We further assess PRIME’s capacity to mitigate memristor read noise using its input-aware dynamic early stop mechanism. The memristive switching and transport mechanisms are illustrated in Fig. 5A. The charge transport through nanoscale conducting channels that are formed due to electrochemical reactions. This results in two types of noise: programming and read noise. The programming noise is due to random ionic motions in channel formation and rupture (*48*), giving rise to cycle-to-cycle and device-to-device variation in programming conductance (Fig. 5B), which can be effectively addressed by our proposed pruning optimization. The read noise is the temporal fluctuation of memristor conductance due to charge trapping and detrapping (e.g., random telegraphic noise) and thermal noise (*49*, *50*) (Fig. 5C).

To demonstrate PRIME’s effectiveness in mitigating memristor read noise, we experiment on various early stop thresholds with different levels of simulated Gaussian read noise (Fig. 5, D and E; see note S7 for the theoretical analysis of the impact of read noise on PRIME, and fig. S22 for simulated conductance fluctuations). PRIME demonstrates remarkable stability in terms of model performance within the typical memristor read noise range of 0.01 to 0.03 (see definition of noise scale in Materials and Methods). In high-noise scenarios (e.g., 0.08 and 0.1), PRIME shows more performance degradation in inpainting (Fig. 5E), while the impact on classification is relatively minor (Fig. 5D). In addition, the study shows that variations in early stop thresholds affect timesteps and accuracy, especially under high-noise conditions, because confidence thresholding of PRIME dynamically balance timesteps and network performance.

## DISCUSSION

Although ANNs have shown unprecedented development, they still cannot parallel the brain in terms of adaptability and energy efficiency. PRIME offers a potential solution via the hardware-software codesign. In terms of hardware, the memristive neuromorphic computer mimics the in-memory computing of the brain. In addition, it also practices pruning optimization that is inspired by biological structural plasticity, making it robust to both programming and read noise of memristors. In terms of software, inspired by brain’s dynamic adjustment of computational depth, we introduce an input-aware dynamic early stop policy for SNN using confidence score, which further boosts energy efficiency and inference speed.

Evaluations on neuromorphic datasets reveals that PRIME matches the accuracy of static weight-optimized SNNs in software, while exhibiting notable advantages in energy consumption and inference speed. PRIME achieves 37.83× energy efficiency improvements and 77.0% computational load savings in neuromorphic image classification. This performance motivates the application of PRIME to complex tasks like image inpainting using a spiking VAE. PRIME shows markedly improved energy efficiency in these tasks, with 62.50× reduction in energy consumption compared to baseline SNNs and 12.5% computational load savings with minimal reduction in performance. This is corroborated by the similar IS achieved by PRIME and the software baseline, and PRIME further exhibits excellent scalability on larger and deeper neural networks. In addition, PRIME effectively mitigates memristor programming and read noise, a notable challenge in emerging neuromorphic hardware. In conclusion, PRIME offers a precise, energy-efficient, and low-latency framework for future neuromorphic computing.

## MATERIALS AND METHODS

### Fabrication of resistive memory chip

Using the advanced 40-nm technology node, the engineered resistive memory device showcases a sophisticated 512 × 512 crossbar configuration. This intricate arrangement features resistive memory elements strategically positioned between four and five metal layers, using a backend-of-line fabrication technique. Each cell within this array is meticulously crafted, consisting of bottom and top electrodes (BE and TE), complemented by a dielectric layer made of transition-metal oxide. The fabrication process begins with the precise patterning of the BE via, which boasts a 60-nm diameter, achieved through photolithography and subsequent etching. This is followed by the deposition of TaN using physical vapor deposition techniques, capped with a 10-nm TaN buffer layer for enhanced stability. A thin, 5-nm layer of Ta is then applied and subjected to oxidation, culminating in the formation of an 8-nm-thick TaO*_x_* dielectric layer. The construction of the TE involves a carefully sequenced deposition of 3-nm Ta and 40-nm TiN, also via physical vapor deposition. The fabrication process is finalized with the deposition of the requisite interconnection metals, adhering to conventional logic processing protocols. Within this architecture, cells aligned in the same row are interconnected through BE, whereas column-aligned cells share TE connectivity. A postfabrication annealing step, conducted for 30 min at 400°C under vacuum conditions, substantially enhances the chip’s performance. This meticulous manufacturing process yields a resistive memory chip with exemplary performance metrics, characterized by its high operational yield and exceptional endurance capabilities.

### Hybrid analog-digital hardware system

The innovative hybrid system merges analog-digital technologies, integrating a 40-nm resistive memory chip with a Xilinx ZYNQ system-on-chip (SoC). This SoC amalgamates a field-programmable gate array (FPGA) with an advanced reduced instruction set computer machines (ARM) processor, all mounted on a printed circuit board for cohesive operation. The resistive memory chip is designed to function in three distinct modes, each crucial for the edge pruning topology optimization: electroform mode, reset mode, and multiplication mode.

In the electroform mode, a controlled dielectric breakdown is initiated within the resistive memory arrays, effectively creating random conductance matrices. This is achieved by biasing all source lines (SLs) to a predetermined programming voltage, delivered by an eight-channel digital-to-analog converter (DAC; DAC80508 from Texas Instruments) boasting a 16-bit resolution. Meanwhile, bit lines (BLs) are grounded, and word lines receive biasing from the DAC to enforce a compliance current across the cells, thereby averting a hard breakdown (fig. S5; see note S3 for discussion about the role of transistors). The nuances of the SL voltage amplitude and duration are key to shaping the postbreakdown conductance distribution and its sparsity.

Transitioning to the reset mode enables the reversion of a resistive memory cell to its nonconductive state, wherein a selected BL is biased via the DAC, the corresponding SL is grounded, and the remaining SLs are left in a floating state. The multiplication mode involves the utilization of a four-channel analog multiplexer (CD4051B, Texas Instruments) coupled with an 8-bit shift register (SN74HC595, Texas Instruments), which collectively apply a DC voltage across the BLs of the resistive memory chip.

Throughout each phase of training, the chip undergoes readings, and the resultant multiplication values, manifested as currents from the SLs, are transformed into voltages. This conversion is facilitated by trans-impedance amplifiers (OPA4322-Q1, Texas Instruments) and analog-to-digital converters (ADS8324, Texas Instruments, with a 14-bit resolution), with the processed data subsequently relayed to the Xilinx SoC for advanced computation. The FPGA component of the SoC is intricately designed to manage the resistive memory operations and facilitate data exchange with the ARM processor via a direct memory access control unit, using double-data rate memory access. Furthermore, the FPGA is tasked with the hardware implementation of certain neural network functions, including activation and pooling, enhancing the system’s overall computational efficacy.

### Reset and set operations

#### Edge pruning topology optimization

The process of synaptic pruning within this system is physically manifested by transitioning the relevant differential pairs of resistive memory into an off-state via the reset operation. Conversely, the reactivation of these synaptical weights is facilitated by restoring the resistive memory cells to their conductive states through a set operation. This set operation is executed by administering uniform pulses, characterized by a 3.3 V amplitude and a 300 ns duration, to the BL of the resistive memory array. This procedure revives the previously pruned cells, reintegrating them into the active subnetwork. On the other hand, the reset operation is effected through the application of uniform pulses, this time with a 2.6 V amplitude and a 400 ns duration, to the SL of the resistive memory array, effectively eliminating the conductive pathway.

It is crucial to highlight the substantial distinction between the off-state and the conducting state of these cells. This differentiation underscores the fact that programming the cells to precise conductance values is not requisite for the system’s functionality, allowing for a more flexible and efficient approach to the modulation of conductive states within the network.

### PRIME model

#### Spiking neuron model

The iterative LIF spiking model (*51*) is used, which is a LIF model solved using the Euler method.

$$\begin{array}{c} u_{t}=\tau_{\text{decay}}u_{t-1}\left(1-o_{t-1}\right)+x_{t}\\ o_{t}=\Theta \left(u_{t}-V_{\mathrm{th}}\right) \end{array}$$ (1)

where τ_decay_ represents membrane decay, *u*_*t*_, *u*_*t*−1_, *o*_*t*_, and *o*_*t*−1_ are the membrane potential and spike output (i.e., 0 or 1) at timestep *t* and *t* − 1. *x*_*t*_ denotes the weighted sum of spikes from the connected neurons, where $x_{j,t}=\sum_{j}\;w_{j}o_{j,t}$. Θ(*x*) represents the heaviside step function, which will generate a spike when *x* > 0. *V*_*th*_ is the threshold potential. Because of the discontinuity of the heaviside step function, we use the approach of pseudo-derivative to solve the issue. In detail, we approximate it as follows

$$\frac{\partial o_{t}}{\partial u_{t}}=\frac{1}{a}\text{sign}\left(|u_{t}-V_{\mathrm{th}}|<\frac{a}{2}\right)$$ (2)

where *a* is a hyperparameter defined as 1 within the context of this study.

#### Pruning topology optimization with random weights

In the pruning topology optimization method (fig. S23), there are two sets of parameters, i.e., randomly distributed weights *W* and pop-up scores *S*. Initially, an SNN with random weights is established using an analog resistive memory chip. Unlike the traditional codesign model, where the weights *W* are expensively tuned, this configuration involves learning a pop-up score *s* for each synaptic weight *w*, while maintaining the weights in their initial random values (fig. S24; see note S8 for comparisons between memristor-based initialization and other popular initializations).

The pruning process can be divided into two phases, the forward pass and the backward pass. On the forward pass, the hardware synapses are selected with top-*k*% highest pop-up scores in each layer according to the predefined sparsity, leading to a pop-up score–based subnetwork. Inputs are then fed into the subnetwork for forward propagation and loss evaluation. The input of neuron *i* in layer *l* at timestep *t* can be computed as

$$I_{i,t}=\sum_{j\in V^{l-1}}\;w_{\mathrm{ij}}o_{j,t}H\left(s_{\mathrm{ij}}\right)$$ (3)

where the *j* neuron in layer *l* − 1 is connected with the *i* neuron via the synaptic weight *w*_*ij*_, *o*_*j*,*t*_ denotes the output of the neuron *j* at timestep *t*, and the *H*(*s*_*ij*_) = 1 if *s*_*ij*_ is in the top-*k*% pop-up scores in layer *l*. On the backward pass, the general digital processor calculates the loss function’s gradients to optimize the scores of all synapses with the random weights fixed. The pop-up score is updated using the straight-through estimator (*29*, *52*) and backpropagation through time (BPTT) (fig. S25)

$$s_{\mathrm{ij}}\leftarrow s_{\mathrm{ij}}-\alpha \frac{\partial L}{\partial s_{\mathrm{ij}}}$$ (4)

where *s*_*ij*_ and α denote the pop-up score between the connected neuron *i* and *j* and the learning rate. $\frac{\partial L}{\partial s_{\mathrm{ij}}}$ is the partial derivative of loss (*L*) with respect to the pop-up score *s*_*ij*_ (see note S9 for details). These processes are repeated until a well-performed subnetwork is selected from the randomly initialized neural network.

#### Dynamic confidence thresholding method

The dynamic confidence thresholding method is input dependent, which dynamically decides which timestep to early stop for each input sample during inference, leading to faster, lower overhead, more energy-efficient edge computing. Consequently, the number of timesteps varies for each input, potentially allowing for a reduction in the average timesteps required per inference.

In the dynamic confidence thresholding method, the confidence metric is calculated on the basis of the output and then used to determine the optimal point to prematurely conclude the inference process. The confidence calculation varies across different tasks. Considering the classification tasks, given an input *X* with a label *Y*, the prediction probability distribution for each timestep *t* is usually calculated by Softmax, $P\left(\bar{Y}=Y\right)=\text{softmax}\left[f\left(X_{t}\right)\right]=\left[p_{1},p_{2},\ldots ,p_{N}\right]$, where $\bar{Y}$ is the predicted output label, *N* is the number of categories. Then, the confidence is defined as $\bar{P_{t}}=\text{max}\left(P\right)$

$$\bar{P_{t}}=\text{max}\left[\frac{e^{f{\left(X_{t}\right)}_{i}/\alpha }}{\sum_{n=1}^{N}\;e^{f{\left(X_{t}\right)}_{i}/\alpha }}\right]i=1,2,\ldots ,N$$ (5)

where $\bar{P_{t}}$ represents the calculated confidence of timestep *t*, α denotes a scale parameter designed to prevent the saturation of confidence levels. Given a predefined threshold β_1_, the inference process will terminate early, using the output at this timestep for prediction, if the confidence reaches or exceeds β_1_ (i.e. $\bar{P_{t}}\geq \beta_{1}$).

Considering the inpainting task, for a given inpainting input image *X*, the output at each timestep is the reconstructed image $\bar{X_{t}}$. The confidence for this task is determined by the consistence calculation

$$\bar{P_{t}}=∥X_{t}-X_{t-1}∥$$ (6)

where ∥⋅∥ represents the *L*1 norm, which is used to quantify the difference between two images at consecutive two timesteps. When the predefined threshold β_2_ is defined, the inference process will terminate early when the condition $\bar{P_{t}}<\bar{P_{t-1}}<\beta_{2}$ is met. This implies that when the change in consecutively generated images becomes minimal, indicating a negligible difference, the process is considered to have reached its termination point.

### Details of the experiments

#### Classification on neuromorphic image dataset

The event-based dataset, N-MNIST (*35*) is used to evaluate the performance of our model. The N-MNIST dataset is a spiking version of the MNIST dataset, created using a DVS mounted on a pan-tilt unit. It comprises 60,000 training samples and 10,000 testing samples, distributed across 10 classes representing digits from “0” to “9.” Each sample in this dataset includes on and off spike events and is represented in a resolution of 34 × 34 pixels, spanning a duration of 300 ms. As shown in Fig. 3A, the 12C5-P2-64C5-P2 architecture is configured. The detailed training settings and default parameters of PRIME on this task is shown in tables S5 and S8, respectively.

The event-based dataset, DVS128 Gesture (*41*) is used to evaluate the scalability of our model. The DVS128 Gesture dataset is a neuromorphic dataset designed for gesture recognition tasks using DVS. The dataset consists of 11 different human actions, including hand clapping, hand waving, and arm rotation, among others. Each sample in this dataset includes on and off spike events and is represented in a resolution of 128 × 128 pixels. As shown in fig. S16, the spiking VGG-11 architecture is configured. The detailed training settings and default parameters of PRIME on this task is shown in tables S6 and S9, respectively.

In addition, the proper memristor array size for pruning in classification is discussed in note S2 (figs. S26 and S27 and tables S11 and S13).

#### Evaluation metric for classification

*Accuracy*. $\text{Accuracy}=\frac{\sum_{i}\;1\left(y_{i}={\hat{y}}_{i}\right)}{n}$, where *y*_*i*_ represents the predicted output for sample *i*, ${\hat{y}}_{i}$ denotes the ground truth, and *n* is the total number of samples.

Average timesteps: $\text{Average}\;\text{ts}=\frac{\sum_{i}\;{\mathrm{ts}}_{i}}{n}$, where ts_*i*_ denotes the inference timesteps for the sample *i*, and the *n* represents the total number of samples.

*ARI*. $\text{ARI}=\frac{\sum_{\mathrm{ij}}\;\left(\begin{array}{c} n_{\mathrm{ij}}\\ 2 \end{array}\right)-\left[\sum_{i}\;\left(\begin{array}{c} a_{i}\\ 2 \end{array}\right)\sum_{j}\;\left(\begin{array}{c} b_{j}\\ 2 \end{array}\right)\right]/\left(\begin{array}{c} N\\ 2 \end{array}\right)}{\frac{1}{2}\left[\sum_{i}\;\left(\begin{array}{c} a_{i}\\ 2 \end{array}\right)+\sum_{j}\;\left(\begin{array}{c} b_{j}\\ 2 \end{array}\right)\right]-\left[\sum_{i}\;\left(\begin{array}{c} a_{i}\\ 2 \end{array}\right)\sum_{j}\;\left(\begin{array}{c} b_{j}\\ 2 \end{array}\right)\right]/\left(\begin{array}{c} N\\ 2 \end{array}\right)}$, where *n*_*ij*_ represents the number of elements in both cluster *i* and cluster *j*, *a*_*i*_ and *b*_*j*_ is the sum of elements in row *i* and column *j*, indicating the total elements in cluster *i* and *j*, *N* is the total number of elements, and $\left(\begin{array}{c} n\\ 2 \end{array}\right)$ denotes the binomial coefficient, representing the number of unique pairs that can be formed from *n* items. The adjusted Rand index (ARI) is a measure used to evaluate the similarity between two data clusterings. A higher ARI value, closer to 1, indicates greater similarity between the two clusterings, suggesting a more accurate clustering process.

*Energy consumption*. The detailed components of energy consumption and their calculation methods are illustrated in table S15.

#### Inpainting on image dataset

The MNIST (*45*) dataset is composed of single-channel grayscale images, representing digits from 0 to 9, thus encompassing 10 distinct classes. Each image in this dataset is characterized by a resolution of 28 × 28 pixels. The dataset is divided into two subsets: 60,000 images for training and 10,000 for testing. In the inpainting task, as illustrated in Fig. 4A, the central 8 × 8 pixel region of each image is obscured. These modified images serve as inputs to a spiking-VAE (*42*), which aims to reconstruct the original, unaltered image (the ground truth). The network’s configuration follows the same structure as outlined in Kamata’s work. However, a notable modification is made in the number of channels in both the encoder and decoder components of the network; They are adjusted to 32 channels, a reduction from the originally specified 64 and 128 channels. The detailed training settings and default parameters of PRIME on image inpainting is presented in tables S7 and S10, respectively.

Fashion-MNIST (*47*) consists of 70,000 grayscale images, each with a resolution of 28 × 28 pixels. The images represent 10 different categories of fashion items, such as T-shirts, trousers, pullovers, dresses, coats, sandals, shirts, sneakers, bags, and ankle boots. Each category contains 7000 images, with 60,000 images in the training set and 10,000 images in the test set. As illustrated in fig. S21, the network’s configuration follows deeper and larger structures. The detailed training settings and default parameters of the experiment is presented in tables S7 and S10, respectively. In addition, the proper memristor array size for pruning in inpainting is discussed in note S2 (figs. S26 and S28 and tables S12 and S13).

#### Evaluation metric for inpainting

*Reconstruction loss*. $L_{\text{reconstruction}}=\text{MSE}\left(x,\hat{x}\right)$, where *x* and $\hat{x}$ distinctly represent the ground truth and reconstructed images, respectively.

$\text{IS}=\text{exp}\left(E_{x∼p_{g}}\left[D_{\text{KL}}\left(p\left(y|x\right)\|p\left(y\right)\right)\right]\right)$, where **x** is the generated data, *p*_*g*_ is the model’s generated data distribution, *p*(*y*) is the marginal class distribution over the generated data, and *p*(*y*∣**x**) represents the conditional class distribution given the generated sample **x**, typically obtained from an Inception model. The IS is commonly used to evaluate the quality of images generated by models like VAEs. The IS measures the diversity and quality of the generated images, where a higher score generally indicates better image quality and more variety in the generated samples.

*Fréchet inception distance*. Fréchet inception distance (FID) is a metric commonly used to assess the quality of generated images in generative models, similar to the IS. FID quantifies the similarity between the feature representations of real and generated images using a pretrained Inception network. It is calculated as $\mathrm{FID}=∥\mu_{r}-\mu_{g}{∥}^{2}+\text{Tr}\left[\Sigma_{r}+\Sigma_{g}-2{\left(\Sigma_{r}\Sigma_{g}\right)}^{1/2}\right]$, where μ_*r*_ and μ_*g*_ are the mean feature vectors of the real and generated images, respectively, and *a*_*r*_ and Σ_*g*_ are their covariance matrices. FID measures both the quality and diversity of the generated images, with lower values indicating better performance. It captures the perceptual similarity between the real and generated image distributions, providing a comprehensive assessment of the generative model’s performance. The FID results are depicted in table S14.

*Energy consumption*. The detailed components of energy consumption and their calculation methods are illustrated in table S15.

#### Memristor read conductance simulation

The memristor conductance with different levels of read noise is simulated using Gaussian noise, which is formulated as

mem_g=mem_g+A×noise_scale×mem_g (7)

where the mem_*g* denotes the memristor conductance, *A* is a random variable following a normal distribution with mean 0 and variance 1, and noise_scale represents the level of read noise in the memristor.

## Supplementary Materials

### This PDF file includes:

Supplementary Text Figs. S1 to S28 Tables S1 to S15 References
