# Supplementary material for: Topology optimization of random memristors for input-aware dynamic SNN
Source: Sci Adv. 2025 Apr 16;11(16):eads5340. doi: 10.1126/sciadv.ads5340 (PMC12002125; doi:10.1126/sciadv.ads5340)
Supplement: Supplementary file 1 — Supplementary Text Figs. S1 to S28 Tables S1 to S15 References [file sciadv.ads5340_sm.pdf]

Science Advances
AAAS

Supplementary Materials for

Topology optimization of random memristors for input-aware dynamic SNN

Bo Wang *et al.*

Corresponding author: Zhongrui Wang, wangzr@sustech.edu.cn; Xiaojuan Qi, xjqi@eee.hku.hk; Dashan Shang, shangdashan@ime.ac.cn

Sci. Adv. **11**, eads5340 (2025)
DOI: 10.1126/sciadv.ads5340

This PDF file includes:

Supplementary Text
Figs. S1 to S28
Tables S1 to S15
References

S1. Supplementary Text

Supplementary Note 1. Comparisons between memristor pruning and precise memristor programming

1.1 Precise memristor programming (Memristor Conductance Fine-tuning)

The programming of memristor conductance is typically achieved by applying pulses with specific widths and voltage amplitudes across the device. In our experiment, the voltage across the 1T1R cell is kept constant, while the gate voltage of the transistor within the 1T1R cell is adjusted. This modulates the channel conductance of the transistor, which controls the voltage division between the transistor and the memristor, thereby programming the memristor to the target conductance (Fig. S1a).

Given the gate voltage, the memristor conductance follows a quasi-normal distribution rather than a specific value due to programming stochasticity, as shown in Fig. S1b. The mean value of this distribution exhibits a linear relationship with the gate voltage (Fig. S1a). As a result, precise memristor programming requires multiple iterative write and read cycles to accurately achieve a reasonable conductance level due to this inherent stochasticity. The programming procedure for the memristor is illustrated in Fig. S1c. Programming a memristor can be viewed as sampling from a quasi-normal distribution defined by the gate voltage. Each programming pulse adjusts memristor conductance which can be considered as a sample from this distribution. If the sampled conductance falls within an acceptable range, the programming process terminates.

Impact of the acceptable range on programming count. The probability of successfully writing a memristor cell to a target conductance is highly influenced by the chosen acceptable conductance range. As shown in Fig. S2a, narrower acceptable ranges (e.g., ±0.005 $\times10^{-3}$S) result in a lower probability of success, while wider ranges (e.g., ±0.02 $\times10^{-3}$S) increase the probability. Fig. S2b illustrates how the probability of writing the memristor conductance within the acceptable range increases with additional programming trials, reflecting the inherent trade-off between acceptable range and the number of pulses required for precise tuning. For example, to reach a probability of 0.6, only 2 pulses are needed for the widest acceptable range (±0.02 $\times10^{-3}$S), whereas 9 pulses are required for a narrower range (±0.005 $\times10^{-3}$S). Additionally, as the desired probability increases, the required number of pulses (N) grows for a given range. For instance, achieving probabilities of 0.6 and 0.8 requires 5 and 9 pulses, respectively, for the same range (±0.005 $\times10^{-3}$S). In contrast, our pruning method requires 1 (50% synapse) or 2 (50% synapse) programming pulses for each connected(pruned) synapse, significantly reducing the number of programming pulses needed.

Energy estimation for single memristor programming. The energy required for memristor programming is determined by the applied voltage, the device resistance, and the duration of the programming pulse. For simplicity, we estimate the energy consumption by using the formula: $E = \frac{V^2 t}{R}$, where V is the programming voltage, t is the pulse duration, and R is the resistance of the memristor. For the given parameters: - The SET operation uses an average voltage of 1.2 V, a resistance of 10 kΩ, and a pulse duration of 10 ms. - The RESET operation uses an average voltage of 1.8 V, a resistance of 100 kΩ, and the same pulse duration of 10 ms. To simplify the estimation, we calculate the energy for both SET and RESET, then take the average value as the energy required for a single memristor programming operation: $E = 8.82 \times 10^{-10}$ J.

1.2 Analysis on memristor-based neural networks

We further demonstrate the advantages of our method over precise memristor programming for neural networks by comparing their accuracy and energy consumption on the N-MNIST classification task according to the network setup in Table S11.

Analysis of precise programming pulse numbers in experimentally measured programming stochasticity across different acceptable ranges. The results depicted in Fig. S3a-b illustrate the accuracy and energy consumption of memristor-based networks programmed with varying acceptable ranges as a function of programming trials in the presence of programming stochasticity (Here we simulated with the experimentally measured programming stochasticity, quasi-normal distributed conductance with 38.4 uS standard distribution). Narrower acceptable ranges provide higher accuracy but require additional programming trials, thereby increasing energy consumption and time costs. Compared to the pruned network, memristor-programmed networks with broader acceptable conductance ranges (e.g., ±0.05) fail to achieve comparable accuracy (Fig. S3a). While narrower ranges (e.g., ±0.005) can achieve similar accuracy through multiple programming trials (N = 25), this comes at a higher cost in terms of energy and time (Fig. S3b). When compared to the Pruned Network 1 (see Table S11 for network details), which achieves even higher accuracy (Fig. S3a), precise memristor programming requires significantly more programming trials to reach similar accuracy (i.e., N = 31 for acceptable conductance range ±0.005), resulting in considerably higher energy and time costs (Fig. S3b).

Analysis of precise programming pulse numbers in different simulated programming stochasticity in acceptable range 0.005. To better evaluate the effect of programming stochasticity on the number of programming trials (N) required for precise memristor programming, we simulated various levels of programming stochasticity within an acceptable conductance range of ±0.005 (Fig. S3c-d). The results show that as programming stochasticity decreases, fewer programming pulses are needed to achieve accuracy comparable to that of our pruned networks. For Pruned Network 1, when the programming stochasticity is 29.8 µS, precise memristor programming achieves similar accuracy (Fig. S3c) with an equivalent number of programming pulses (as indicated by the energy consumption in Fig. S3d). When programming stochasticity exceeds 29.8 µS, our pruning method becomes more energy-efficient than precise memristor programming; however, for values below 29.8 µS, precise programming becomes more efficient. For the original network, under the same acceptable range of ±0.005, when programming stochasticity is 15.1 µS, precise programming achieves comparable accuracy (Fig. S3c) with similar programming pulses (as shown in the energy consumption Fig. S3d). For programming stochasticity values greater than 15.1 µS, our pruning method is more energy-efficient, while for values lower than 15.1 µS, it becomes less efficient.

These results demonstrate that our method offers advantages over precise programming in both accuracy and programming energy in the current experimentally programming stochastically. And our pruning method can also be extended, to some extent, to memristor arrays with smaller programming stochasticity.

Supplementary Note 2. Discussion on the Array Size Requirements for Different Tasks

2.1 The theoretical analysis of the array size.

The Strong Lottery Ticket Hypothesis (SLTH) posits that within a sufficiently overparameterized neural network, there exists a subnetwork that, when initialized randomly and without training, can achieve the same accuracy as a fully trained target network. Building upon existing theoretical works on SLTH (30, 53–55), we discuss the required network size for over-

parameterized neural networks with random memristor weights, considering both fully connected and convolutional neural networks.
Key results are summarized as follows:

- **Fully connected neural network**: Given $\epsilon, \delta > 0$, a memristor-based fully-connected (FC) random network with width $O(d\log(\frac{dl}{\min\{\epsilon,\delta\}}))$ and depth $2l$, can be pruned to approximate any weight-optimized neural network with width d and depth l within error ϵ, and with probability at least $1-\delta$.

- **Convolutional neural network**: Given $\epsilon, \delta > 0$, a memristor-based random convolutional neural network (CNN) with $O(k\log(\frac{kl}{\min\{\epsilon,\delta\}}))$ parameters and $2l$ layers, can be pruned to approximate any weight-optimized CNN with k parameters and l layers within error ϵ, and with probability at least $1-\delta$.

2.1.1 Fully connected neural network analysis

In PRIME, we utilize the fully connected networks with the LIF spiking neuron, $\Theta(u) = 0, 1$. The random weights are initialized using the programming stochasticity of memristors, which follows a quasi-normal distribution. The memristor-based spiking neural network (SNN) is defined as follows,

$$G_M^t(s^t) = L^{t,0}(s^{t,0}) \circ \cdots \circ L^{t,l}(s^{t,l}), \tag{S1}$$

where $L^{t,i} = H(W^{L(i)}s^{t,i})$ denotes the i-th layer of the memristor-based spiking neural network G_M^t, $s^{t,i}$ represents the received spike of layer i at time t. Recognizing the fact that the firing rates of the vanilla SNN can be estimated by activations of an artificial neural network (ANN) with corresponding architecture and weights (56–58), we remove the temporal term for the ease of theoretical analysis. The network is reformulated as follows,

$$G_M(x) = W^l \circ H(\cdots \circ H(W^0 x)), \tag{S2}$$

where x represents the activations (firing rates of spiking neurons), H is the activation function, which is typically ReLU (56–58). SNNs can be viewed similarly to ANNs through this simplification.

Drawing from previous SLTH research on ANNs (30, 53), we establish a pruning theorem for our SNNs. Theorems 1 and 2 of (30, 53) build on three key assumptions: the parameter initialization scheme, the network input, and the activation function. In (30, 53), the parameter initialization G follows a uniform distribution $U[-1, 1]$, which is loosely constrained and centered around zero. This initialization approach can be generalized to an arbitrary distribution, including the quasi-normal distribution of memristor differential pair conductance. Secondly, the input to the network in (30, 53) is restricted to values between 0 and 1. Similarly, in our case of SNNs, as described above, the input can be interpreted as the spiking rates, which also falls within the range of 0 to 1. Thirdly, the activation function used in (30, 53) is ReLU, which is compatible with the activation function in our network. Therefore, based on the theorem 1 and 2 of publications (30, 53), we can establish the theorem below:

- **Theorem 1**: For a target weight-optimized neural network F of width n with l layers, and let G_M be a $2l$-layer memristor-based neural network, with weights randomly initialized from quasi-normal distribution,

$$\begin{aligned} F(x) &= W^l \circ H(\cdots \circ H(W^0 x)), \\ G_M(x) &= W^{2l} \circ H(\cdots \circ H(W^0 x)) \end{aligned} \tag{S3}$$

where $||W||_2 \leq 1$ in F, and the width of W^{2i} and W^{2i-1} in G_M are represented as follows,

$$W^{2i} : d_i \times Cd_{i-1} \log\left(\frac{d_{i-1}d_i l}{\min\{\epsilon, \delta\}}\right) \tag{S4}$$

$$W^{2i-1} : Cd_{i-1} \log\left(\frac{d_{i-1}d_i l}{\min\{\epsilon, \delta\}}\right) \times d_{i-1}, \tag{S5}$$

where C is a constant. Let two variables $\epsilon, \delta \in (0, 1)$. Then, with probability at least $1 - \delta$, there exists a subnetwork $\tilde{G}_M$ of G_M such that,

$$\sup_{x \in \mathcal{X}} \left| F(x) - \tilde{G}_M(x) \right| \leq \epsilon \tag{S6}$$

Theorem 1 shows that there exists a pruned subnetwork $\tilde{G}_M$, derived from the randomly initialized memristor-based neural network G_M, which can achieve comparable performance with the target weight-tuning network F. This result is summarized in section 2.1-**Fully connected neural network**:

- **Fully connected neural network**: Given $\epsilon, \delta > 0$, a memristor-based fully-connected (FC) random network with width $O(d \log(\frac{dl}{\min\{\epsilon,\delta\}}))$ and depth $2l$, can be pruned to approximate any weight-optimized neural network with width d and depth l within error ϵ, and with probability at least $1 - \delta$.

2.1.2 Convolutional neural network analysis.

Similar to FC neural network, we simplify the SCNN to its CNN counterpart according to the spiking rate, $G_M : [0, 1]^{D \times D \times c_0} \to \mathbb{R}^{D \times D \times c_l}$, for the sake of simplicity

$$G_M(x) = K^l * H(K^{l-1} * \cdots * H(K^1 * X)), \tag{S7}$$

where $K^i \in \mathbb{R}^{d_i \times d_i \times c_{i-1} \times c_i}$, and the convolutions have no bias and are suitably padded with zeros. A proven theorem in (54) demonstrates that a subnetwork from an overparameterized random convolutional neural network can achieve similar accuracy to a weight-tuned convolutional neural network. The same assumptions are made as in the fully connected (FC) network described in Section 2.1.1. Building on Lemma 1, Lemma 2 and theorem 1 from previous work (54), and based on the assumption analysis above, we derive the following theorem:

- **Theorem 2**: Let $d_i, c_0, l, n \in \mathbb{N}$, and $\epsilon, C \in \mathbb{R}_{>0}$. For $i \in [l]$, let F be the target weight-optimized neural network,

  $$F(x) = K^l * H(K^{l-1} * \cdots * H(K^1 * X)), \tag{S8}$$

  where $K^l \in \mathbb{R}^{d_l \times d_l \times c_{l-1} \times c_l}$, and $\|K^l\|_2 \leq 1$.

  Let $d_i, c_i, n_i \in \mathbb{N}$, where $n_i \geq Cc_i \log\left(\frac{c_{i-1}d_i^2 l}{\min\{\epsilon,\delta\}}\right)$, and $G_M : [0, 1]^{D \times D \times c_0} \to \mathbb{R}^{D \times D \times c_{2l}}$ be a $2l$-layered random memristor-based CNN given by

  $$G_M(x) = L^{2l} * H(L^{2l-1} * \cdots * H(L^1 * X)), \tag{S9}$$

  where for $i \in [l]$ the kernels L^{2i}, L^{2i-1}, i.i.d. the quasi-normal distribution, are random variables of shape $1 \times 1 \times n_i \times c_{i-1}$ and $d_i \times d_i \times c_{i-1} \times n_i$, respectively.

Finally, let $\tilde{G}_M$ be subnetwork pruned from G_M. There exists a constant $C > 0$ such that, with probability at least $1 - \delta$, we have the following theorem,

$$\sup_{x \in [0,1]^{D \times D \times c_0}} |F(x) - \tilde{G}_M(x)| \leq \epsilon, \tag{S10}$$

According to the previous publication (54), Theorem 2 can be summarized as shown in section 2.1-**Convolutional neural network**:

- **Convolutional neural network**: Given $\epsilon, \delta > 0$, a memristor-based random convolutional neural network (CNN) with $O(k \log(\frac{kl}{\min\{\epsilon,\delta\}}))$ parameters and $2l$ layers, can be pruned to approximate any weight-optimized CNN with k parameters and l layers within error ϵ, and with probability at least $1 - \delta$.

2.2 Numerical Experiments on Array Size for Different Tasks

We conducted experiments to validate Theorem 2 on the N-MNIST classification and MNIST inpainting tasks. As illustrated in Fig. S26, the process begins with a target weight-tuned network (Step 1). Based on Theorem 2, we determine a set of neural network sizes for random networks by adjusting ϵ and δ in the parameter count restriction $k \log\left(\frac{kl}{\min \epsilon,\delta}\right)$ (Step 2). Next, randomly initialized memristor weights are acquired based on the estimated size, M (Step 3). Then, pruning mask is optimized in software according to the acquired memristor weights (Step 4), and the memristors corresponding to pruned weights are RESET to zero conductance (Step 5) before evaluating performance using memristor hardware. Our experimental results demonstrate that these over-parameterized networks, optimized through pruning, achieve performance comparable to weight-tuned networks (See Fig. S27 and S28).

2.2.1 N-MNIST-based classification

We will now illustrate the execution of these steps for the N-MNIST classification task and present the final performance outcomes.

Step 1: We use the well-performing weight-tuned network in our manuscript as the example. This network architecture, Input-12C5-P2-64C5-P2-10, with 42,840 parameters, is commonly adopted for classifying N-MNIST dataset (59, 60).

Step 2: We estimate the number of the required parameters for pruned network under the constraints of Theorem 2, i.e., $k \log\left(\frac{kl}{\min\{\epsilon,\delta\}}\right)$. We explore different sets of variables (ϵ, δ) to estimate parameter counts according to the weight-tuned network($k = 42,840$):

- $\delta = 0.05$, $\epsilon = 0.05$, $\log\left(\frac{kl}{\min\{\epsilon,\delta\}}\right) = 6.23$, $k \log\left(\frac{kl}{\min\{\epsilon,\delta\}}\right) = 267,060$
- $\delta = 0.05$, $\epsilon = 0.01$, $\log\left(\frac{kl}{\min\{\epsilon,\delta\}}\right) = 6.93$, $k \log\left(\frac{kl}{\min\{\epsilon,\delta\}}\right) = 297,004$
- $\delta = 0.05$, $\epsilon = 0.001$, $\log\left(\frac{kl}{\min\{\epsilon,\delta\}}\right) = 7.93$, $k \log\left(\frac{kl}{\min\{\epsilon,\delta\}}\right) = 339,844$
- $\delta = 0.01$, $\epsilon = 0.05$, $\log\left(\frac{kl}{\min\{\epsilon,\delta\}}\right) = 6.93$, $k \log\left(\frac{kl}{\min\{\epsilon,\delta\}}\right) = 297,004$
- $\delta = 0.001$, $\epsilon = 0.05$, $\log\left(\frac{kl}{\min\{\epsilon,\delta\}}\right) = 7.93$, $k \log\left(\frac{kl}{\min\{\epsilon,\delta\}}\right) = 339,844$

Based on the estimated number of parameters, we then design three over-parameterized random networks (Table S11) by widening or deepening the weight-tuned network. Each Pruned Network in Table S11 corresponds to specific values of ϵ and δ, allowing us to explore how variations in these parameters affect the network size.

Step 3: Memristors are electroformed to obtain random synaptic weights, which then form the over-parameterized network.

Step 4: An optimal pruning mask with 50% sparsity for the corresponding network, tailored for N-MNIST classification, is obtained in software using our proposed pruning algorithm.

Step 5: The optimal pruning mask is physically mapped onto memristors, with pruned memristors being hard RESET to zero conductance.

Experimental results. Fig. S27a shows that networks configured with different values of δ and ϵ consistently achieve accuracy within the predefined error margins (as determined by ϵ), underscoring the validity of the network sizes estimated by our theorem. Furthermore, as depicted in Fig. S27b, the box plots of accuracy over 10 trials for each configuration align well with the theoretical probability bound (1 - δ). In addition, as the network size increases, the gap between the highest and lowest accuracy scores becomes narrower. The results (Fig. S27) also show that selecting the network size parameters (ϵ, δ) involves a trade-off. Smaller parameters (ϵ, δ) result in a larger network size but higher accuracy, and vice versa.

2.2.2 MNIST-based inpainting

We also demonstrate how these steps are applied in the the MNIST inpainting task.

Step 1: We use the well-performing weight-tuned network in our manuscript as the example. The original encoder of PRIME in MNIST-inpainting is input-32C3-32C3-32C3-128, with 84,256 parameters, and the decoder of PRIME is latent-32C3-32C3-32C3-32C3-1C3, with 93,472 parameters. The additional parameters of decoder compared to encoder are from the output layer. For consistency, we use the encoder parameter count 84,256 as k to estimate the encoder and decoder parameters.

Step 2: We estimate the number of the required parameters for pruned networks under the constraints of Theorem 2, i.e., $k \log\left(\frac{kl}{\min\{\epsilon,\delta\}}\right)$. We also explore the effects of different combinations of ϵ and δ on the parameter count using the weight-tuned network in our manuscript ($k = 84,256$):

- $\delta = 0.1$, $\epsilon = 0.1$, $\log\left(\frac{kl}{\min\{\epsilon,\delta\}}\right) = 6.40$, $k \log\left(\frac{kl}{\min\{\epsilon,\delta\}}\right) = 539,467$
- $\delta = 0.001$, $\epsilon = 0.1$, $\log\left(\frac{kl}{\min\{\epsilon,\delta\}}\right) = 8.40$, $k \log\left(\frac{kl}{\min\{\epsilon,\delta\}}\right) = 707,979$
- $\delta = 0.0001$, $\epsilon = 0.1$, $\log\left(\frac{kl}{\min\{\epsilon,\delta\}}\right) = 9.40$, $k \log\left(\frac{kl}{\min\{\epsilon,\delta\}}\right) = 792,235$
- $\delta = 0.1$, $\epsilon = 0.001$, $\log\left(\frac{kl}{\min\{\epsilon,\delta\}}\right) = 8.40$, $k \log\left(\frac{kl}{\min\{\epsilon,\delta\}}\right) = 707,979$
- $\delta = 0.1$, $\epsilon = 0.0001$, $\log\left(\frac{kl}{\min\{\epsilon,\delta\}}\right) = 9.40$, $k \log\left(\frac{kl}{\min\{\epsilon,\delta\}}\right) = 792,235$

Based on the estimated number of parameters, we then design three over-parameterized random networks (Table S12) by widening or deepening the weight-tuned network. Each Pruned Network in Table S12 corresponds to specific values of ϵ and δ, allowing us to explore how variations in these parameters affect the network size.

Step 3: Memristors are electroformed to obtain random synaptic weights, which then form the over-parameterized network.

Step 4: An optimal pruning mask with 50% sparsity for the corresponding network, tailored for N-MNIST classification, is obtained in software using our proposed pruning algorithm.

Step 5: The optimal pruning mask is physically mapped onto memristors, with pruned memristors being hard RESET to zero conductance.

Experimental results. The results (Fig. S28a) show that networks configured with different values of δ and ϵ achieve image reconstruction accuracy within the predefined error bounds (set by ϵ). Similarly, as shown in Fig. S28b, the box plots of reconstruction loss over 10 trials for each configuration consistently align with the theoretical probability bound (1 - δ), further confirming the validity of our approach. In addition, by adjusting ϵ and δ, we can control the network size to balance task requirements and resource efficiency, demonstrating the flexibility and relevance of our provided steps.

2.3 Cost-effectiveness of our approach

It is true that our method, which leverages the randomness of memristor programming, requires a larger array compared to a precisely programmed weight-tuned approach (e.g. using conventional digital hardware) to achieve comparable performance. However, memristor arrays offer relatively low fabrication costs and high integration density, resulting in significantly reduced manufacturing expenses and a more compact overall array size. Additionally, the pruning method eliminates the need for costly precise programming, leading to substantially lower energy consumption.

Low hardware cost: While an increased array size increases chip area consumption, memristors are cost-effective to manufacture in large volumes compared to conventional digital memory. Using a 100 nm process node, we performed a rough cost estimation based on standard assumptions about fabrication costs and die area utilization (61,62) (Table S13). The fabrication of both RRAM and SRAM is compatible with standard CMOS processes, and for standard CMOS technology, the manufacturing cost can generally be considered proportional to the area. As the footprint of RRAM is significantly smaller than that of SRAM (e.g. SRAM $> 120f^2$, RRAM $4f^2$, see Table S13), RRAM has a significant cost advantage over SRAM in terms of cost per bit, particularly in large-scale production. Consequently, even with a larger array size, memristor-based solutions remain highly cost-competitive. For example, achieving similar performance, the fabricated array area of RRAM for pruned random networks is significantly smaller than that of SRAM utilizing tuned weights (Table S13).

Avoid expensive precise programming and low energy cost: Precise programming of memristor arrays typically requires multiple write-verify cycles to achieve target weight values. This process introduces considerable time overhead and increases energy consumption. In contrast, our pruning-based approach mitigates the need for such precise programming by leveraging the intrinsic variability of memristor devices to produce Gaussian-distributed weights. As a result, this method reduces energy consumption and shortens programming times to a single programming cycle, offering a reduced programming cost even with the increased array size. A detailed comparison of precise memristor programming versus memristor pruning can be found in Supplementary Note 1.

To summarize, while our method requires a larger array compared to precisely programmed memristor arrays, the overall benefits in terms of low fabrication cost and reduced programming complexity offer a compelling argument for its practicality.

Supplementary Note 3. Discussion on the hardware system

3.1 The role of the transistor during 1T1R array operation

The role of the transistor in 1T1R array is clarified as follows:

Programming. The transistor (T) plays a crucial role in limiting the current during the programming process (Fig. S5a). It ensures that the memristor is not subjected to excessive current, thus preventing potential damage or hard breakdown. This is accomplished by tuning the gate voltage of the transistor, which regulates the compliance current during programming.

SNN Forward. Spikes from the previous layer are applied to the word lines (transistor gates) of the current layer, activating the transistors and allowing the spike voltage to generate weighted currents on the corresponding memristors (Fig. S5b). These weighted currents are then summed by peripheral TIA circuits and digitized by the ADC (Fig. S6a). The digital LIF neuron circuit updates its state (Fig. S6b-d; see Section 3.2.1 for details about the neuron circuit) and generates post-spikes, which are subsequently transmitted to the FPGA. For the first layer (Fig. S6a right), the direct encoding layer, is enable to receive spike input or analog input. If the input signal is an analog value, we convert it to the binary numbers represented by a spike train using time-division multiplexing.

3.2 Hardware implementation of LIF neuron

3.2.1 Digital Circuit Implementation of LIF

Our designed digital LIF neuron, illustrated in Fig. S6b, implements the discrete LIF dynamics described in Eq. (S16) and (S17). The circuit consists of four main components and two multiplexers (MUXs). The first component is on-chip SRAM, which stores the membrane potential V_{mem}. The second component is a comparator that evaluates V_{mem} against a predefined threshold V_{th}. When $V_{\mathrm{mem}} \geq V_{\mathrm{th}}$, the comparator activates the post-spike and resets the membrane potential, respectively. The third component, a leaky module, performs a fixed-point multiplication of V_{mem} with a leakage factor τ, simulating the membrane potential leakage. The fourth component is an adder, which updates V_{mem} for the next time step by summing the weighted input sum I_{ext} from an ADC and the leaky V_{mem} of the current time step. For the non-spiking LIF neuron (Fig. S6c) in the output layer, only the membrane potential storage, adder, and leaky module are utilized, so the neuron accumulates input spikes into membrane potential without emitting spikes. Fig. S6d shows the simulated temporal response of the digital LIF neuron. In the simulation, the digital input (yellow) modulates the membrane potential (green). When the membrane potential exceeds the threshold, a post-spike signal (red) is generated, followed by a reset of the membrane potential. This response demonstrates the spike generation dynamics and the reset behavior characteristic of the digital LIF neuron.

The digital LIF neuron operates at a clock frequency of 100 MHz, with a typical input pulse width matching the clock cycle duration of 10 ns.

3.2.2 Analog Circuit Implementation of LIF

Compared to digital LIF neurons, analog implementation of LIF neurons may bring certain advantages in terms of power consumption and speed, although it increase hardware complexity. Here, we also present an design of analog LIF circuit and analyze its operational functions.

The implementation of LIF neuronal model using an analog circuit is shown in Fig. S7a. This CMOS-based neuron accommodates both excitatory (positive memristor cell) and inhibitory (negative memristor cell) inputs, which are combined through a differential pair to form the synaptic input current. The current scaling blocks adjust the synaptic current, which is then integrated on the membrane capacitor. When the membrane potential reaches the threshold, a comparator and refractory buffer generate a post-spike signal and reset the membrane capacitor. Fig. S7b presents the simulated waveform of the proposed analog neuron. Randomly generated input spike trains are applied to the wordlines (WLs) of the memristor crossbar. The simulation demonstrates the evolution of the membrane potential and the gener-

ation of post-spikes (action potentials), confirming the functionality of the analog LIF neuron model.

The operation speed of our analog LIF neuron is 20MHz. The typical input pulse width of the analog neuron is 50 ns and the amplitude is 1.8V. Based on Fig. S7b, the neuron receives excitatory (red) and inhibitory (blue) input pulses with a typical width of approximately 50 ns. These inputs modulate the membrane potential (V_{mem}, purple trace), increasing with excitatory inputs and decreasing with inhibitory inputs. When V_{mem} exceeds the threshold, a post-spike (green trace) is emitted.

3.3 System latency analysis

3.3.1 System diagram

The hardware system diagram is shown in Fig. S6a, b. Each network layer consists of a 1T1R array that encodes synaptic connection weights, peripheral circuits (TIA + SAR ADC) for digitizing the weighted summation current, and a digital LIF neuron that simulates the behavior of biological neurons. To balance the trade-off between the area and speed of the peripheral circuits, multiple rows of the array share a single set of peripheral circuits. In this system, a multiplexer (MUX) is required to select the shared circuits, and a spike distributor is employed to control the timing of delivering neuron output spikes to different rows in the next layer.

3.3.2 Analysis of system pipeline and latency

We present the latency analysis for implementing the LIF neuron using digital circuit (Fig. S6) and analog circuit (Fig. S7) as below.

Latency analysis for the system with digital LIF neuron. The latency of the system is determined by the input layer and subsequent SNN (Spiking Neural Network) layers, as shown in Fig. S8a. The input layer features a three-stage pipeline, while the subsequent SNN layers operate with a two-stage pipeline. Specifically:

- Input Layer Pipeline:
  - Stage 1 Raw Data Processing: Processes the input analog signals using a time-division multiplexing approach.
  - Stage 2 Spike-Weight Multiplication: Multiplies the input spikes with their corresponding weights.
  - Stage 3 State Update: The digital neuron circuits update their internal states based on the results of the multiplication.
- Subsequent SNN Layer Pipeline:
  - Stage 1 Spike-Weight Multiplication: Multiplies the input spikes with their corresponding weights.
  - Stage 2 State Update: The digital neuron circuits update their internal states based on the results of the multiplication.

For both layers, the primary contributors to system latency are the shared peripheral circuits, which introduce delays when digitizing the analog currents of spike-weight multiplication, and the computational time required for the neurons to update their states.

Therefore, the overall system latency involves a trade-off between power consumption and chip area. The number of shared peripheral circuits (TIA + ADC) is a key factor influencing

latency. Increasing the number of ADCs can reduce the time required for Spike-Weight Multiplication in a single layer, but incurs a larger chip area overhead. Additionally, the state update time of digital neurons also affects latency. The speed of neuron state updates is determined by power consumption and chip area, further impacting the trade-off in system design (Fig. S8b).

The system latency estimation for each layer can be estimated mathematically as:

$$\begin{aligned} \text{Latency} =& L1 + N \times \\ & ((\text{Bit number} \times \text{ADC clock cycle}) + (\text{Neuron pipeline layer} \times \text{Neuron clock cycle})) \end{aligned} \tag{S11}$$

where:

- $L1$ is the latency of TDM block, where only first layer includes
- N is $\frac{\text{number of columns}}{\text{number of ADC}}$
- Bit number refers to the number of bits used, where 16 bits is used
- ADC clock cycles, about 100ns
- Neuron pipeline layers, 3
- Neuron clock cycles, about 10ns

Latency analysis for the system with analog LIF neuron. In addition to digital neurons, we also design fully analog circuits to implement neurons (Fig. S7). Compared to digital LIF neurons, analog implementation of LIF neurons may bring certain advantages in terms of power consumption and speed, although it increases hardware complexity. Our analog LIF circuit design is illustrated in Fig. S7.

The latency characteristics of systems employing analog neurons differ from those using digital neurons. In analog SNNs, the systems are spike-driven and do not require ADCs. Thus, the response latency for a single spike input equals the settling time of the analog neuron, as shown in S7c. However, the overall inference latency of the system is primarily dictated by the average input timesteps multiplied by the duration of the predefined timestep unit (63). The encoding scheme decides the average number of timesteps, while the timestep unit(pulse width) is determined by the circuit's response speed, which depends on the slew rate of the circuit. For a circuit with fixed parasitic capacitance, the slew rate is approximately proportional to power consumption (64). In high-speed circuits where parasitic effects become significant, the relationship between slew rate and power consumption deviates from linearity, (Fig. S7d).

Supplementary Note 4. Discussion on spike stream pre-processing of N-MNIST

4.1 Generation and Original Temporal Resolution of N-MNIST

Event camera (65, 66) is a dynamic vision sensor that detects changes in pixel intensity, providing a biologically inspired representation of visual data. The N-MNIST dataset (35) is a dynamic transformation of the static MNIST images into neuromorphic event-based data using event cameras. The original temporal resolution of the N-MNIST dataset is 1 μs, reflecting the high temporal precision of the event capture.

Different from the conventional camera (65, 66), which records the absolute brightness (Fig. S10a), event cameras operates by detecting changes in pixel intensity (Fig. S10a) with theoretical microsecond temporal resolution. Events are generated when intensity changes exceed a

defined threshold, recording the time, position, and polarity of the change (positive for "ON" events and negative for "OFF" events). This mechanism produces a continuous, asynchronous stream of events corresponding to high-speed changes in the visual scene.

The principle of pixel intensity changes in event cameras is described by the image constancy constrain, which relates changes in pixel brightness to image motion and spatial gradients. This relationship is expressed as:

$$I_t = -I_x V_x - I_y V_y, \tag{S12}$$

where:

- I_t is the derivative of image intensity with respect to time,
- I_x and I_y are the intensity gradients in the x and y directions on the image plane,
- V_x and V_y are the velocities in the x and y directions on the image plane.

This equation shows that the changes in pixel brightness (I_t) arises from the combination of image motion (velocities V_x and V_y) and spatial gradients (I_x and I_y), leading to event generation when significant changes occur.

To create the N-MNIST dataset (35), a neuromorphic sensor is rotated by motors while observing static 2D MNIST images (Fig. S10b). The induced motion generates intensity changes in the sensor's field of view, triggering events. The dataset captures these events with an original temporal resolution of 1 microsecond, offering a precise and dynamic visual representation.

4.2 Methods of Compressing Events to 10 Time Bins

The frame binning compression method (36,67–72) (Fig. S11a top) and voxel binning compression method (65) (Fig. S11a bottom) are both widely used for pre-processing neuromorphic datasets. Here, we apply the frame binning compression approach as described in Spiking-Jelly (36). Neuromorphic datasets record data in the form $E(x_i, y_i, t_i, p_i)$, which includes the event's spatial coordinates (x_i, y_i), timestamp t_i, and p_i its polarity (+1 for "ON", -1 for "OFF"). To compress the data into frames, the total number of events, N, is divided into T segments of approximately equal size, integrating these events into corresponding frames. Here, T also serves as the simulation timesteps.

For each frame $F(j)$ with channels p and spatial coordinates (x, y), the pixel value is calculated by aggregating events with indices between j_l and j_r, defined as:

$$j_l = \left\lfloor \frac{N}{T} \right\rfloor \cdot j, \tag{S13}$$

$$j_r = \begin{cases} \lfloor \frac{N}{T} \rfloor \cdot (j+1), & \text{if } j < T-1 \\ N, & \text{if } j = T-1 \end{cases} \tag{S14}$$

The pixel intensity $F(j, p, x, y)$ is then computed by summing the indicator function $I_{p,x,y}(p_i, x_i, y_i)$ over the selected event range:

$$F(j, p, x, y) = \sum_{i=j_l}^{j_r-1} I_{p,x,y}(p_i, x_i, y_i), \tag{S15}$$

where $\lfloor \cdot \rfloor$ represents the floor operation, and $I_{p,x,y}(p_i, x_i, y_i)$ is an indicator function that equals 1 only when $(p, x, y) = (p_i, x_i, y_i)$. This integration compresses the long spike sequence into a shorter sequence, thereby facilitating further processing.

Voxel binning (65, 73–77) (Fig. S11a bottom) is a compression technique designed to reduce the size of event data by segmenting events into discrete voxel grids. Each voxel corresponds to a specific spatial region and a uniform time interval, maintaining the spatiotemporal properties of the original events. This method effectively converts high temporal resolution event data into a lower resolution format, optimizing it for processing by neural networks running on conventional computers.

Due to the better performance achieved by frame binning (Fig. S11b), we choose frame binning as the compression method in PRIME.

4.3 Rationale for Compression into Short Time Bins

Following prior work on analog in-memory computing, we adopt conventional time binning, as it is not the primary focus of this study. As outlined in Table S1, the use of binning is motivated by the following three reasons:

Time binning facilitates efficient and effective SNN training. While SNNs are inherently compatible with the asynchronous nature of event data due to their ability to process binary signals (78), they face several critical limitations. First, the lack of efficient training algorithms tailored for the asynchronous nature of SNNs necessitates the use of Backpropagation Through Time (BPTT) for gradient computation. This introduces challenges such as gradient explosion or vanishing, as well as prolonged training times, particularly when processing inputs with long timesteps (79–81). Additionally, the significant number of timesteps significantly increases memory costs during training (82, 83). These limitations can render BPTT impractical when the original event streams are retained. Additionally, surrogate gradient functions are typically employed to address the discontinuity of spiking neurons, yet their performance is highly sensitive to the number of timesteps (79–81). Retaining original event data, containing tens of thousands of timesteps, exacerbates cumulative errors in surrogate gradient approximations (84, 85). Therefore, event stream compression techniques, including the frame binning (36, 67–72) and voxel binning (65, 73–77), are widely adopted in current SNN training.

Time binning reduces hardware cost. Retaining uncompressed event data significantly increases the number of timesteps in spike trains, leading to higher hardware inference costs (63, 86). Event compression effectively mitigates these costs by reducing the number of timesteps, despite the additional overhead introduced by binning operations. Notably, the additional overhead associated with event binning is lower than the costs incurred when event data is left uncompressed. Additionally, as demonstrated in (87, 88), compressing events into shorter timesteps reduces both transmission bandwidth and backend energy consumption.

Summary of early literature. Numerous studies have explored the application of analog circuits and asynchronous input processing in SNNs (Table S1). All of the approaches involve compressing event data into shorter timebins.

Supplementary Note 5. Comparisons between PRIME and other pruning methods in SNNs

We provide a detailed comparison of the proposed memristor pruning method (labeled as ”Ours”) with several reported pruning techniques for SNNs, as summarized in Table S2 and S3. This comparison includes key metrics like goal, pruning method, sparsity, accuracy, and parameter count across two datasets, N-MNIST and DVS-Gesture.

Method and Goal: The proposed memristor pruning method focuses on network optimization by leveraging the programming stochasticity of memristors for synapse pruning to improve network performance. In contrast, other methods are predominantly aimed at network compression, focusing on reducing storage and computational requirements while maintaining

accuracy. Notably, these methods like Grad R (89), SNDP (90), Deep R (89) are designed to achieve a compressed network.

Pruning Method: Our approach utilizes unstructured synapse pruning with random weights, a distinctive method that capitalizes on the memristor's inherent properties. Other methods utilize various pruning strategies. For example, Grad R (89) and ADMM (91) adopt unstructured synapse pruning with weight tuning. Some techniques, such as SD-SNN (92) and DPAP (93), involve both neuron and synapse pruning, with SD-SNN further incorporating unstructured neuron pruning and synapse regeneration. Structured pruning strategies are adopted by methods such as SCCD (94), Network Slimming (95), BinWSNN (96)), which target channels or patches.

Sparsity: The proposed memristor pruning method achieves a 50% sparsity on both N-MNIST and DVS-Gesture. In contrast, other methods exhibit a wide range of sparsity levels. For instance, Grad R (89) reaches a high sparsity of 75% on N-MNIST, while SCCD (94) achieves only 15.10% sparsity on DVS-Gesture.

Accuracy: Despite 50% pruning with random weights, the proposed memristor pruning method maintains relatively high accuracy, achieving 97.60% on N-MNIST and 97.57% on DVS-Gesture. This demonstrates the effectiveness of our approach, which achieves competitive accuracy without weight tuning. In contrast, other methods experience an accuracy drop after pruning and rely on weight tuning to restore or enhance performance. For instance, methods like SD-SNN (92), and DPAP (93) achieve slightly higher accuracies (99.53% and 99.55% on N-MNIST, 98.20% and 98.56% on DVS-Gesture, respectively) but through weight tuning.

Parameters: The number of parameters for each method is estimated based on the network architectures reported in their respective studies. The proposed memristor pruning method yields one of the lowest parameter counts on N-MNIST (0.021M), showcasing its effectiveness in reducing model size. On DVS-Gesture, the parameter count is higher (6.662M), attributed to the use of a larger VGG-11 model to address the dataset's complexity. Other methods show a range of parameter counts, with SCCD (94) achieving a very low 0.14M on DVS-Gesture, while SD-SNN (92) and DPAP (93) require more parameters (5.470M and 4.422M, respectively).

Resistance to Memristor Programming Stochasticiy: Compared to other SNN pruning methods, our approach exhibits superior resistance to memristor programming stochasticity. This highlights the enhanced suitability of our method for deployment on neuromorphic hardware.

Summary: The proposed memristor pruning method achieves an effective balance among sparsity, accuracy, parameter reduction, and resistance to programming stochasticity. The comparative analysis further underscores the advantages of our approach, highlighting its suitability for the development of memristor-based neural networks.

Supplementary Note 6. Discussion on spikingVAE details and its implementation on memristor crossbar arrays

6.1 Details of spikingVAE

The Fully Spiking Variational Autoencoder (FSVAE) (42) is a spiking neural network (SNN)-based variational autoencoder (VAE) for image generation, improving energy efficiency using SNNs. It employs spiking neurons across all layers and is compatible with neuromorphic hardware.

Fig. S17 illustrates an overview of the model's operation. Initially, the input image x is converted into spike trains $x_{1:T}$ using direct input encoding (97,98). These spike trains are then passed through the SNN encoder, producing encoded spike sequences $x^E_{1:T}$. Subsequently, the posterior processes these encoded spikes to generate latent spike trains $z_{1:T}$ sequentially. The

SNN decoder uses these latent spikes to produce the reconstructed image x'. In the sampling phase, the prior generates $z_{1:T}$, which is processed by SNN decoder to generate image x'.

6.1.1. Building blocks of FSVAE

FSVAE consists of an encoder, a latent sampler, and a decoder, as detailed below.

Encoder: The encoder is an SNN with convolution layers (see structure details in Table. S4). The input image x is converted to spike trains using direct encoding by the first spiking convolutional layer. These spike trains are processed by the SNN encoder, outputting spike trains $x^E_{1:T}$.

Latent Sampling: The posterior receives z_{t-1} and x^E_t as input and outputs latent variables z_t sequentially, using the autoregressive SNN model. The prior just receives z_{t-1} as input and outputs latent variables z_t sequentially, using the autoregressive SNN model.

Decoder: The decoder, an SNN with deconvolution layers (see structure details in Table. S4), receives latent spike trains $z_{1:T}$ to generate the reconstructed image x'.

6.1.2. Spiking Neuron Model

The iterative leaky integrate-and-fire (iLIF) spiking neuron model is utilized, which is a LIF model solved using the Euler method.

$$u_t = \tau_{\text{decay}} u_{t-1}(1 - o_{t-1}) + x_t, \tag{S16}$$

$$o_t = \Theta(u_t - V_{\text{th}}), \tag{S17}$$

where τ_{decay} represents membrane decay, u_t, u_{t-1}, o_t, o_{t-1} are the membrane potential and spike output (i.e., 0 or 1) at time step t and $t-1$. x_t denotes the weighted sum of spikes from the connected neurons, where $x_{j,t} = \sum_j w_j o_{j,t}$. $\Theta(x)$ represents the Heaviside step function, which will generate a spike when $x > 0$.

Additionally, due to the discontinuity of the Heaviside step function, we utilize the approach of pseudo-derivative for error backpropagation. In detail, we approximate it as follows:

$$\frac{\partial o_t}{\partial u_t} = \frac{1}{a}\text{sign}\left(|u_t - V_{\text{th}}| < \frac{a}{2}\right), \tag{S18}$$

where a is a hyperparameter defined as 1 within the context of this study.

6.1.3. Autoregressive Bernoulli Spike Sampling

To implement Autoregressive Bernoulli Spike Sampling, the posterior and prior probability distributions are defined as follows:

$$q(z_{1:T}|x_{1:T}) = \prod_{t=1}^{T} q(z_t|x_{\leq t}, z_{<t}), \tag{S19}$$

$$p(z_{1:T}) = \prod_{t=1}^{T} p(z_t|z_{<t}). \tag{S20}$$

In this framework, $q(z_t|x_{\leq t}, z_{<t})$ and $p(z_t|z_{<t})$ are modeled as Bernoulli distributions that take binary values, which is suitable for SNNs. Since SNNs require binary outputs, the reparameterization trick commonly used in conventional VAEs is not applicable. Instead, FSVAE samples z_t sequentially from $z_{<t}$ by employing an autoregressive SNN model. Sampling is performed from a Bernoulli distribution by randomly selecting one value per channel. Fig. S18

illustrates the sampling process, where the input is z_{t-1} for the prior and (z_{t-1}, x_t^E) for the posterior.

The autoregressive SNN models $q(z_t|x_{\leq t}, z_{<t})$ and $p(z_t|z_{<t})$ produce outputs $\zeta_{q,t}$ and $\zeta_{p,t}$, respectively, defined as:

$$\zeta_{q,t} := f_q(z_{q,t-1}, x_t^E; U_{q,t}) \in \{0,1\}^{kC}, \tag{S21}$$

$$\zeta_{p,t} := f_p(z_{p,t-1}; U_{p,t}) \in \{0,1\}^{kC}. \tag{S22}$$

Here, C denotes the dimensionality of z_t, and k is a natural number that scales the dimensionality. $U_{q,t}, U_{p,t}$ are the sets of membrane potentials of the neurons in f_q, f_p. The notation $\zeta_{q,t}[k(c-1) : kc]$ represents a subset of binary outputs selected from $\zeta_{q,t}$ within the specified range of indices.

Sampling $z_{q,t}$ or $z_{p,t}$ is achieved by randomly selecting one value per channel from $\zeta_{q,t}$ or $\zeta_{p,t}$, respectively:

$$z_{q,t,c} = \text{random\_select}\left(\zeta_{q,t}[k(c-1) : kc]\right), \tag{S23}$$

$$z_{p,t,c} = \text{random\_select}\left(\zeta_{p,t}[k(c-1) : kc]\right), \tag{S24}$$

where $c = 1, \ldots, C$. Thus, $z_{q,t}$ and $z_{p,t}$ are binary vectors in $\{0,1\}^C$.

This approach is equivalent to sampling from the Bernoulli distribution:

$$z_{q,t}|x_t, z_{q,t-1} \sim \text{Ber}(\pi_{q,t}), \tag{S25}$$

$$z_{p,t}|z_{p,t-1} \sim \text{Ber}(\pi_{p,t}), \tag{S26}$$

where

$$\pi_{q,t,c} = \text{mean}\left(\zeta_{q,t}[k(c-1) : kc]\right), \tag{S27}$$

$$\pi_{p,t,c} = \text{mean}\left(\zeta_{p,t}[k(c-1) : kc]\right). \tag{S28}$$

Therefore, the posterior and prior probability distributions are individually defined as the Bernoulli distributions $\text{Ber}(\pi_{q,t})$ and $\text{Ber}(\pi_{p,t})$.

6.1.4. Image-to-Spike Encoding and Spike-to-Image Decoding

- Image-to-Spike Encoding

In image processing tasks, various coding schemes have been proposed for SNNs (97–100), including temporal coding, rate coding, and direct coding. While temporal coding (97, 100) has been successfully applied to shallow SNNs, these methods are challenging to implement effectively at large network sizes and with large-scale datasets. Consequently, SNNs commonly use rate coding (97, 98) and direct (97–99) input encoding as common approaches to represent input stimuli. In our work, we employ direct input encoding rather than rate encoding, as the latter often introduces challenges related to latency, energy consumption, and accuracy (98).

Rate encoding. In rate encoding (Fig. S19a), real-valued pixel intensities are converted to spike trains, with each spike train's firing rate proportional to the pixel intensity (98). To implement this, a Poisson or Bernoulli generator is typically used to translate analog pixel values into spike trains. The generator produces random numbers at each timestep, normalized within the range $\{0, \frac{1}{T}, \ldots, 1\}$, and compares these values with the pixel intensities to generate spikes. However, achieving high fidelity with rate encoding requires a prolonged simulation window, which leads to increased latency, higher energy consumption, and diminished accuracy.

Direct encoding. Direct input encoding (Fig. S19b) provides an efficient approach for processing image data in SNNs (97–99). In this method, the first layer functions as an encoding

layer, capable of handling both spike-based and continuous inputs. For continuous inputs, pixel intensities are directly applied to the SNN's input layer at each timestep. The first convolutional layer, composed of Leaky Integrate-and-Fire (LIF) neurons, acts as both a feature extractor and a spike generator, accumulating weighted pixel values and producing output spikes based on these inputs. For example, when converting a static MNIST image into a 16-timestep spike sequence, the same image is applied over 16 timesteps, allowing the first convolutional layer (Fig. S20a) to encode pixel intensities into spike trains by leveraging the LIF neurons. This approach enhances computational efficiency and accuracy, making it a widely adopted choice for image-based tasks within SNNs.

- Spike-to-Image Decoding

The output layer consists of non-firing neurons, which only accumulate spikes into their membrane potential instead of firing spikes. The membrane potentials are then converted to real-valued image pixels:

$$u_T^{\text{out}} = \sum_{t=1}^{T} \tau^{T-t} \hat{x}_t \tag{S29}$$

The final reconstructed image x' is obtained by applying a non-linear activation function on u_T^{out}, $x' = \tanh(u_T^{out})$.

6.1.5. Loss Function

Variational Autoencoder (VAE): A VAE learns to encode data by maximizing the Evidence Lower Bound (ELBO), given by:

$$\text{ELBO} = \mathbb{E}_{q(z|x)}\left[\log p(x|z)\right] - \text{KL}\left[q(z|x)\|p(z)\right], \tag{S30}$$

In the SNN setting, the latent variables are spike trains, making traditional normal distribution sampling unsuitable. Instead, FSVAE models the latent space using Bernoulli distributions. The ELBO of FSVAE is as follows:

$$\text{ELBO} = \mathbb{E}_{q(z_{1:T}|x_{1:T})}\left[\log p(x_{1:T}|z_{1:T})\right] - \text{KL}\left[q(z_{1:T}|x_{1:T})\|p(z_{1:T})\right], \tag{S31}$$

The first term in Eqn. S31 is the reconstruction loss, which is formulated by $\text{MSE}(x, x')$. The second term, KL divergence, represents the closeness of the prior and posterior probability distributions. Traditional VAEs use KL divergence to close two distributions. In contrast, FSVAE uses MMD (42, 101), which is a more suitable distance metric for spike trains. MMD is formulated as follows:

$$\begin{aligned}\text{MMD}^2\left[q(z_{1:T}|x_{1:T}), p(z_{1:T})\right] = &\ \mathbb{E}_{z,z'\sim q}\left[k(z_{1:T}, z'_{1:T})\right] + \mathbb{E}_{z,z'\sim p}\left[k(z_{1:T}, z'_{1:T})\right] \\ &- 2\mathbb{E}_{z\sim q, z'\sim p}\left[k(z_{1:T}, z'_{1:T})\right],\end{aligned} \tag{S32}$$

where k is a PSP kernel function, which can capture the time information from spike trains. The first-order synaptic model is used as PSP and the update of PSP is formulated as follows:

$$\text{PSP}(z_{\leq t}) = \left(1 - \frac{1}{\tau_{\text{syn}}}\right)\text{PSP}(z_{\leq t-1}) + \frac{1}{\tau_{\text{syn}}} z_t, \tag{S33}$$

where τ_{syn} is the synaptic time constant and $\text{PSP}(z_{\leq 0}) = 0$. Finally, the MMD loss is represented as below. The detailed derivation can be found in (42).

$$\text{MMD}^2\left[q(z_{1:T}|x_{1:T}), p(z_{1:T})\right] = \sum_{t=1}^{T} \left\|\text{PSP}(\pi_{q,\leq t}) - \text{PSP}(\pi_{p,\leq t})\right\|^2, \tag{S34}$$

where $\pi_{p,\leq t}$ and $\pi_{q,\leq t}$ represent the prior and posterior Bernoulli distributions, individually. Finally, the ELBO of FSVAE is calculated as follows:

$$\mathcal{L} = \text{MSE}(x, x') + \sum_{t=1}^{T} \left\|\text{PSP}(\pi_{q,\leq t}) - \text{PSP}(\pi_{p,\leq t})\right\|^2 \tag{S35}$$

6.2 Implementation of spikingVAE on memrisor crossbar arrays

The SpikingVAE system leverages two 512×512 memristor crossbar arrays for in-memory computing (Fig. S20). The weights of convolutional and fully-connected layers (layer 1 through 9) of both the encoder and decoder are physically mapped onto memristor crossbar arrays, as illustrated in Fig. S20. Each random synaptic weight is mapped to a differential conductance pair, denoted as G^+ and G^-.

As shown in Fig. S20b, c and Table. S4, the encoder and decoder layers of SpikingVAE are physically mapped onto memristor arrays of varying sizes, as detailed below:

- **Layer 1**:The weights of Layer 1 have dimensions of (1, 32, 3), corresponding to the input channels, output channels, and kernel size, respectively. The weights of the direct encoding layer are mapped onto two **9×32** (Table. S4) memristor arrays located in the bottom-right corner of the memristor crossbar array for encoder layers (Fig. S20b).

- **Layers 2 and 3**: The weights of Layer 2 and Layer 3 have dimensions of (32, 32, 3). Each of these convolutional layers requires two **288×32** arrays (Table. S4). These layers are placed upper-left in the memristor crossbar array for encoder layers.

- **Layer 4**: The fully connected (FC) Layer 4 has weight dimensions of **512×128** (Table S4). Within the memristor crossbar array for encoder layers, this layer is implemented across two sub-arrays, specifically **180×128×2** and **332×128×2**.

- **Layer 5**: Similarly, this FC layer also has weight dimension of **512×128** (Table S4), requiring subarrays **180×128×2** and **332×128×2** in the memristor crossbar arrays for decoder layers.

- **Layers 6, 7, and 8**: The weights of these three layers have dimensions of (32, 32, 3). The decoder's convolutional layers, each mapped to two **288×32** arrays (Table. S4), are located upper-left in the memristor crossbar array for decoder layers(Fig. S20c).

- **Layer 9**: The output layer occupies two compact **9×32** arrays (Table. S4), placed in the bottom-right corner of the memristor crossbar array for decoder layers.

Supplementary Note 7. Theoretical Analysis of PRIME with Noise

In the inference phase, the pruned subnetwork $\tilde{G}_M$ is subject to read noise. Assume the read noise η_{read} follows a Gaussian distribution $\mathcal{N}(0, \sigma_{read}^2)$. We analyze the impact of this noise on the pruned network's performance.

Let the pruned network's weights affected by the read noise be:

$$W'_{\text{pruned}} = W_{\text{pruned}} + \eta_{read}, \tag{S36}$$

where W_{pruned} is the random weights initialized from memristor conductance differentials, and $\eta_{read} \sim \mathcal{N}(0, \sigma^2_{read})$.

For the pruned subnetwork's output affected by noise $y'_{\text{pruned}} = \tilde{G}_M(x)$, we can derive the mean and variance as follows:

$$\begin{aligned}
\mathbb{E}[y'_{\text{pruned}}] &= \mathbb{E}[\tilde{G}_M(x)] = \mathbb{E}[W'_{\text{pruned}}x] = \mathbb{E}[(W_{\text{pruned}} + \eta_{read})x] = W_{\text{pruned}}x, \\
\text{Var}(y'_{\text{pruned}}) &= \text{Var}(\tilde{G}_M(x)) = \text{Var}(W'_{\text{pruned}}x) = \mathbb{E}[(W'_{\text{pruned}}x)^2] - (\mathbb{E}[W'_{\text{pruned}}x])^2 \\
&= \sigma^2_{read}\|x\|^2.
\end{aligned} \tag{S37}$$

Let the original output be y_{pruned}, and the actual output affected by noise be y'_{pruned}. The error e_{pruned} is:

$$\begin{aligned}
e_{\text{pruned}} &= y'_{\text{pruned}} - y_{\text{pruned}}, \\
\mathbb{E}[e_{\text{pruned}}] &= \mathbb{E}[y'_{\text{pruned}} - y_{\text{pruned}}] = 0, \\
\text{Var}(e_{\text{pruned}}) &= \text{Var}(y'_{\text{pruned}}) = \sigma^2_{read}\|x\|^2.
\end{aligned} \tag{S38}$$

Through this theoretical analysis, it is evident that the read noise impacts the pruned network's output variance, given by $\sigma^2_{read}\|x\|^2$. Under appropriate noise scale, the input-aware early stop policy of PRIME (see **Methods** for details) can automatically adjust to fluctuations in the output induced by noise (see Fig. 5 for a comprehensive experimental demonstration). If the noise decreases y_{pruned}, the sample may not meet the early stop threshold, resulting in continued processing and potentially increasing the number of inference timesteps to ensure accuracy. Conversely, if the noise increases y_{pruned}, early stopping may occur, conserving computational resources while maintaining accuracy.

Supplementary Note 8. Comparisons between different initialization distributions on classification

To evaluate the distributions of different weight initialization schemes, we compared the performance of pruned networks using Kaiming, Xavier, Truncated Normal, and Orthogonal initialization (Fig. S24a). The results demonstrate that the choice of weight initialization significantly impacts model performance. Among these, Kaiming Normal initialization achieved the highest accuracy (97.83%), similar to that our memristors. In contrast, Kaiming Uniform showed a notable decrease in accuracy (94.40%). These findings illustrate that the conductance distribution of memristors is effective for network pruning optimization. To further analyze this, we calculated the KL divergence to quantify the similarity between the distribution of memristor-based weights and those generated by other initialization methods (Fig. S24b). The results reveal that the distribution of memristor-based weights closely resembles those of the Truncated Normal and Kaiming Normal initializations, explaining its comparable performance in achieving high classification accuracy.

Supplementary Note 9. Discussion on update of the pop-up score through BPTT

1. **Backpropagation Through Time:** Our method leverages BPTT to update the pop-up scores in the SNN with loss backpropagation. In SNNs, the update for s_{ij} with BPTT would involve computing the gradients across all previous timesteps. The iterative LIF dynamics, described in Eqn. (S16) and (S17), are unfolded and backpropagated along the computational graph, as illustrated in Fig. S25. Taking the pruned synapse into account and using neuron i as an example, Eqn. S16 and S17 can be revised as follows:

$$u_{i,t} = \tau_{\text{decay}} u_{i,t-1}(1 - o_{i,t-1}) + \sum_j w_{ij} o_{j,t} H(s_{ij}), \tag{S39}$$

$$o_{i,t} = \Theta(u_{i,t} - V_{\mathrm{th}}), \tag{S40}$$

where τ_{decay} represents membrane decay, $u_{i,t}$, $u_{i,t-1}$, $o_{i,t}$, $o_{i,t-1}$ are the membrane potential and spike output (i.e., 0 or 1) at time step t and $t-1$ of neuron i. $\sum_j w_{ij} o_{j,t} H(s_{ij})$ denotes the weighted sum of spikes from the connected neurons. $\Theta(x)$ represents the Heaviside step function, which will generate a spike when $x > 0$. Then, the gradients with T time steps for s_{ij} are calculated by:

$$\frac{\partial L}{\partial s_{ij}} = \sum_{t=1}^{T} \frac{\partial L}{\partial o_{i,t}} \frac{\partial o_{i,t}}{\partial u_{i,t}} \left(\frac{\partial u_{i,t}}{\partial s_{ij}} + \sum_{\tau<t} \prod_{k=t-1}^{\tau} \left(\frac{\partial u_{i,k+1}}{\partial u_{i,k}} + \frac{\partial u_{i,k}}{\partial o_{i,k}} \right) \frac{\partial u_{i,\tau}}{\partial s_{ij}} \right), \tag{S41}$$

where s_{ij} is the pop-up score from neuron j to neuron i and L is the loss. The non-differentiable terms of spiking neuron Θ are replaced by surrogate derivatives, formulated as Eqn. S43. The non-differentiable term H of pruning is solved by the straight-through estimator, i.e. $\frac{\partial u_{i,t}}{\partial s_{ij}} = w_{ij} o_{j,t}$. Therefore, the pop-up score is updated as follows:

$$s_{ij} \leftarrow s_{ij} - \alpha \frac{\partial L}{\partial s_{ij}}. \tag{S42}$$

2. **Surrogate Gradient Function:** The non-differentiability of the Heaviside step function in the LIF model, which generates a spike when $(u - V_{th}) > 0$ (where V_{th} is the threshold potential), poses a challenge for gradient-based optimization. To address this issue, we employ a surrogate gradient function, approximating the derivative as follows:

$$\frac{\partial o_t}{\partial u_t} = \frac{1}{a} \mathrm{sign} \left(|u_t - V_{th}| < \frac{a}{2} \right), \tag{S43}$$

where a is a hyperparameter defined as 1 within the context of this study.

S2. Supplementary Figures

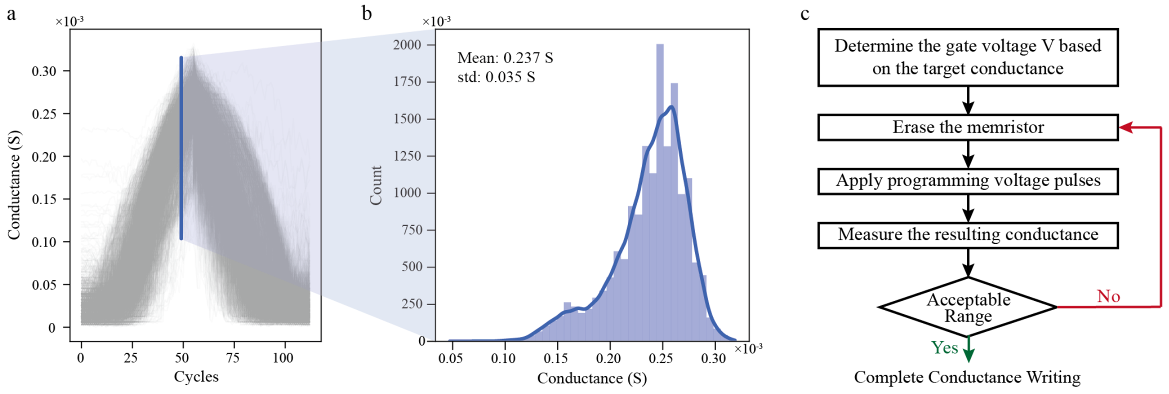

Figure S1: **Programming stochasticity and precise programming flow. a,** The conductance of memristors across programming cycles. Each cycle corresponds to a specific gate voltage. **b,** The distribution of memristors' conductance at cycle 50 (corresponding to a gate voltage of 1.8V), illustrating the stochasticity of memristor programming. **c,** Flowchart of the precise programming process for a single memristor, showing the iterative process of applying voltage and measuring conductance until the desired conductance falls within an acceptable range.

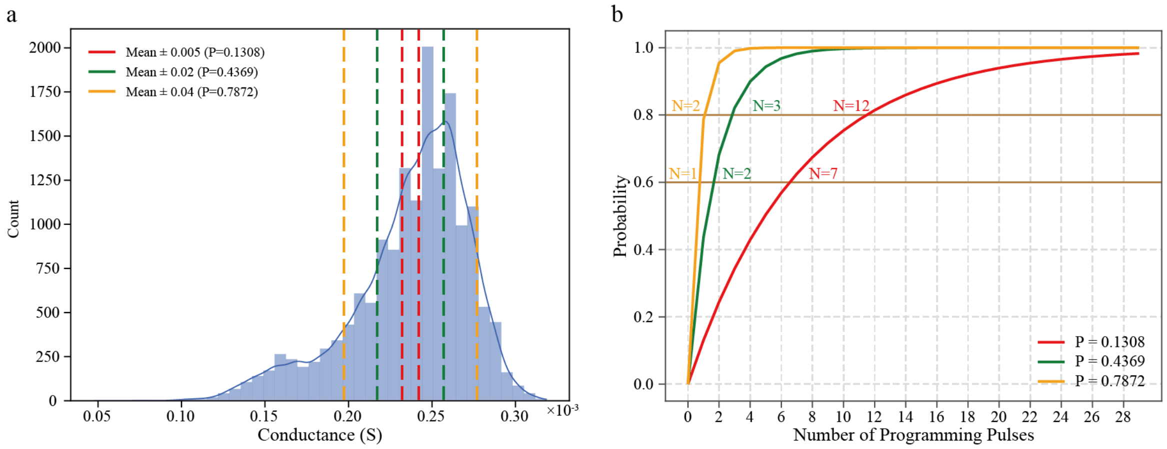

Figure S2: **Probability analysis for writing a memristor to a specific conductance value.** **a,** Probability distribution of the memristor conductance and different acceptable ranges: ±0.005, ±0.02, and ±0.04 $\times 10^{-3}$S. **b,** Cumulative probability of successfully writing the memristor conductance within the acceptable range across programming pulses, showing the impact of different acceptable range thresholds on expected number of programming pulses. The parameter N represents the number of pulses required to reach the given acceptable range with a specific cumulative probability. P represents the probability of writing the memristor conductance within the range using a single programming pulse.

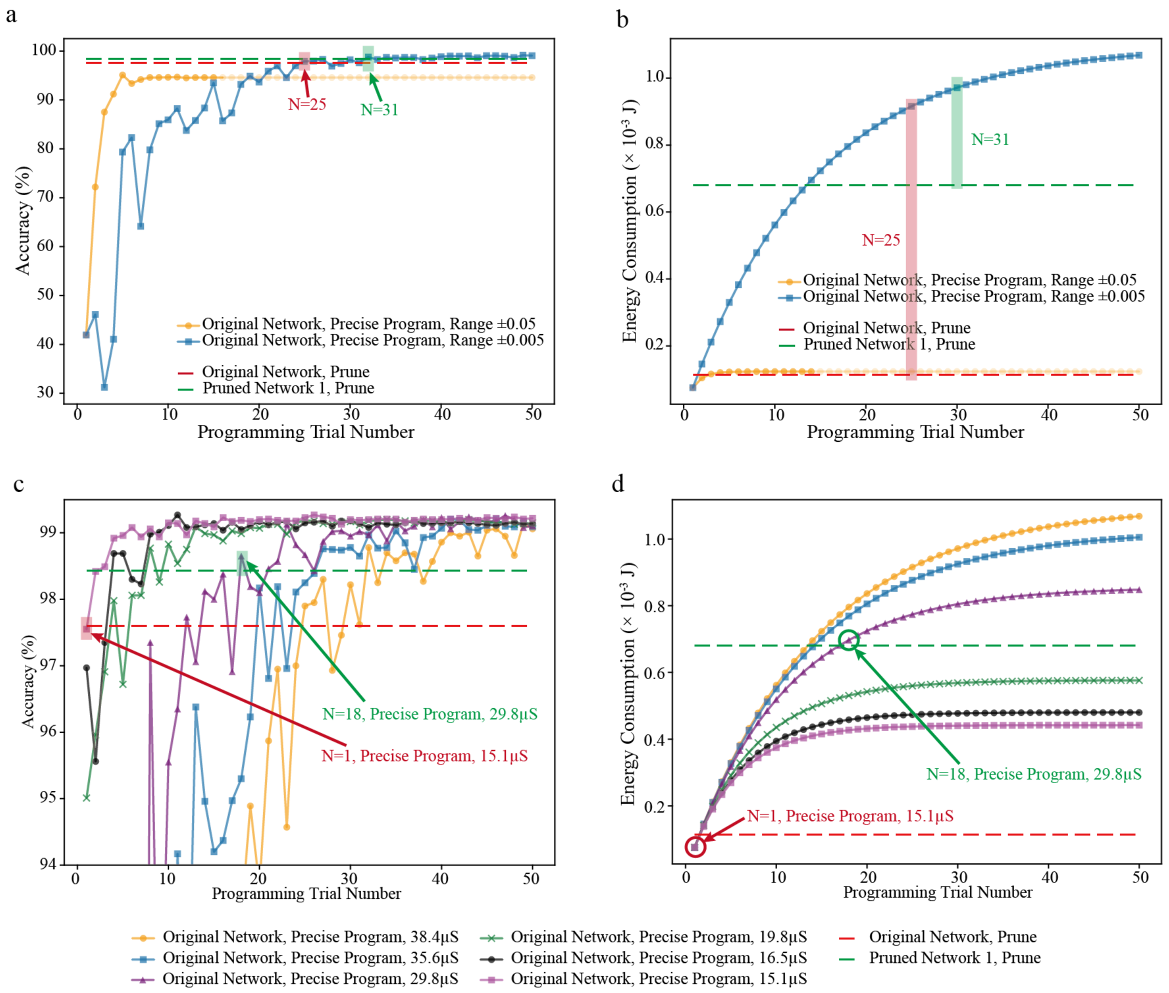

Figure S3: **Accuracy and energy consumption comparisons of different memristor-based networks.** **a,** Accuracy comparison between the precise memristor programming method (which requires multiple programming trials) and the pruning method (which just requires up to two programming trials). N represents the programming trial number for the precise memristor programming. "Range" refers to the acceptable range of the scaled conductance. At $N = 25$, the accuracy of precise programming with an acceptable range of 0.05 matches the accuracy achieved through pruning the original network. At $N = 31$, the accuracy of precise programming with an acceptable range of 0.005 matches the accuracy achieved through pruning the Pruned Network 1 (see Table. S11 for details about Pruned Network 1). **b,** Energy consumption (total number of memristor programming pulses) comparison between precise memristor programming across programming trials and the pruning method. **c,** Accuracy comparison between the precise memristor programming method with different memristor programming stochasticity (which requires multiple programming trials) and the pruning method (which just requires up to two programming trials). N represents the programming trial number for the precise memristor programming. At $N = 1$, the accuracy of precise programming with programming stochasticity 15.1 μS matches the accuracy achieved through pruning the original network. At $N = 18$, the accuracy of precise programming with programming stochasticity 29.8 μS matches the accuracy achieved through pruning the Pruned Network 1 (see Table. S11 for details about Pruned Network 1). **d,** Energy consumption (total number of memristor programming pulses) comparison between precise memristor programming with different programming stochasticity across programming trials and the pruning method.

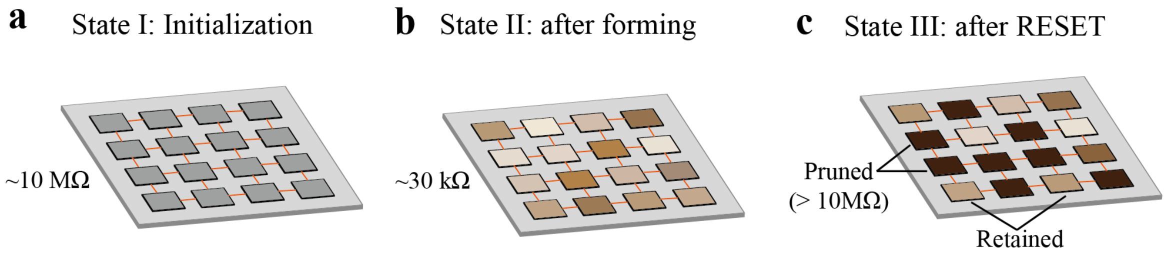

Figure S4: **Random weight initialization and pruning on memristor arrays.** The memristor array physically implements random synaptic weights of PRIME. **a**, The memristors are initially in their pristine states about 10 MΩ. **b**, Following the application of a 3.3 V forming voltage, memristors are programmed into a low-resistance state with a resistance of around 30 kΩ. Owing to the inherent programming stochasticity of memristors, cell condutance follows a quasi-Normal distribution, which is harnessed for physically implementing random weights. **c**, After obtaining the optimal pruning mask from the training phase, memristors with the low pop-up scores are hard RESET to OFF state. The pruned cell resistance increases dramatically above approximately 10MΩ.

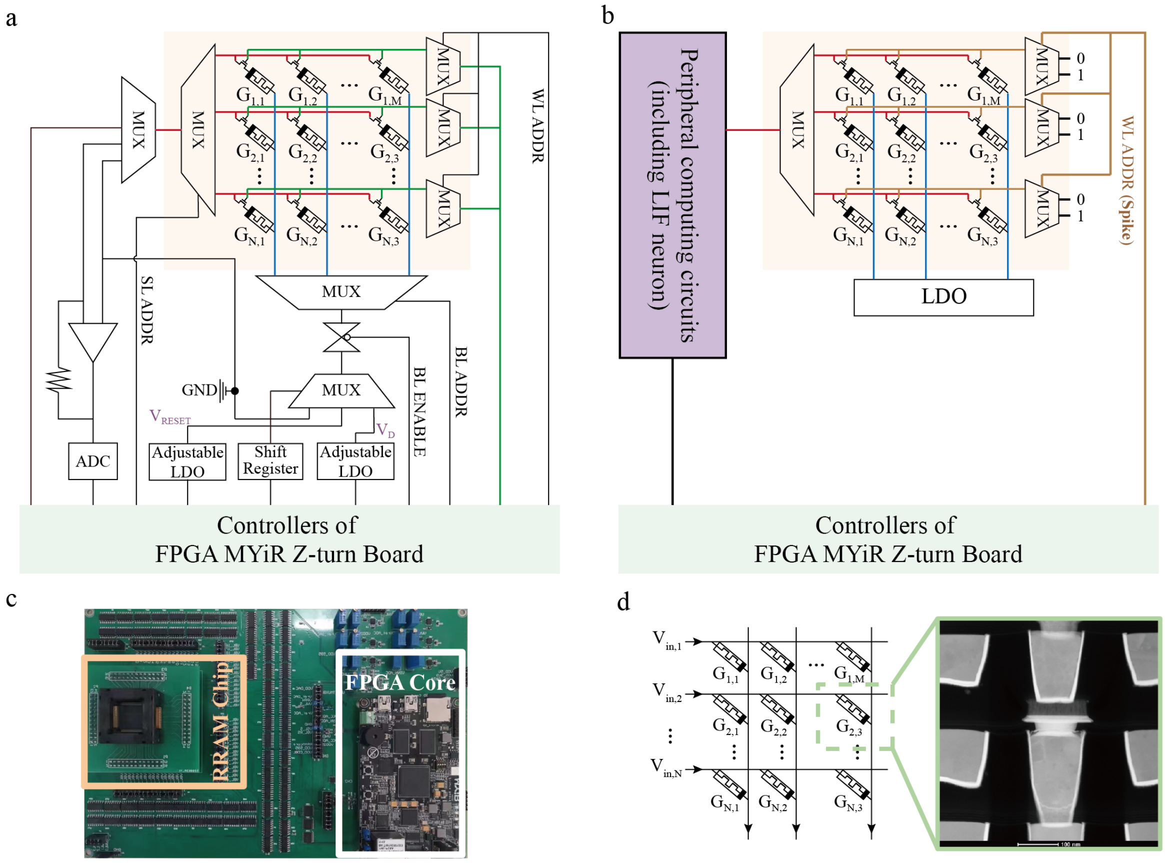

Figure S5: **The hybrid analogue-digital hardware system. a,** The schematic of the hybrid analog-digital computing system in memristor programming phase. **b,** The schematic of the hybrid analog-digital computing system in SNN forward phase. **c,** The photo of the hybrid analog-digital hardware system. **d,** The optical photo of the 1T1R resistive memory array.

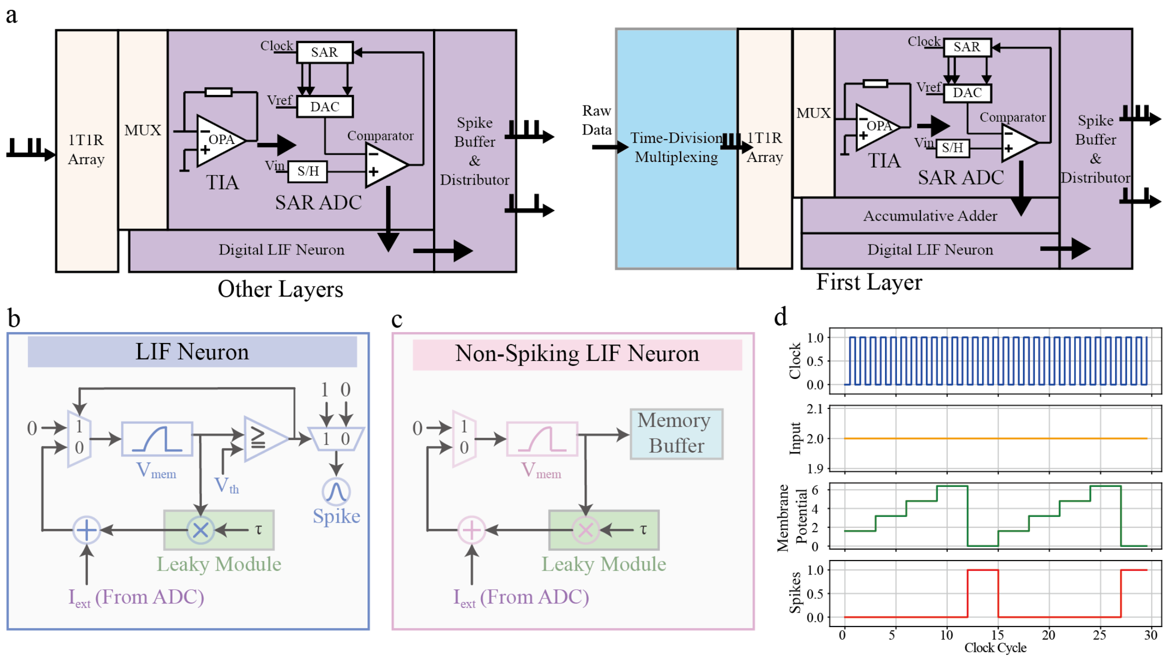

Figure S6: **Overview of the peripheral computing circuits. a, Left:** The system diagram of a single spiking layer. **Right:** The system diagram of the first layer. There is an additional time-division multiplexing block compared to other layers due to its direct encoding function. **b,** Diagram of a LIF neuron circuit that emits spikes or accumulates potential. The diagram represents an LIF neuron with spiking capability, where the membrane potential V_{mem} is updated based on incoming current I_{ext} from an ADC. When V_{mem} exceeds the threshold V_{th}, a spike is generated and the neuron resets. The block labeled as the "Leaky Module" shows the leakage behavior, controlled by parameter τ, which adjusts V_{mem} over time. **c,** Diagram of a non-spiking LIF neuron circuit in the last layer that accumulates input spikes into membrane potential. **d,** Simulated temporal response of the digital LIF neuron. Digital input (yellow) is integrated into the membrane potential (green). When membrane potential exceeds the threshold, a post-spike signal (red) is generated, followed by a reset of membrane potential. This simulation illustrates the neural dynamics of the digital LIF neuron.

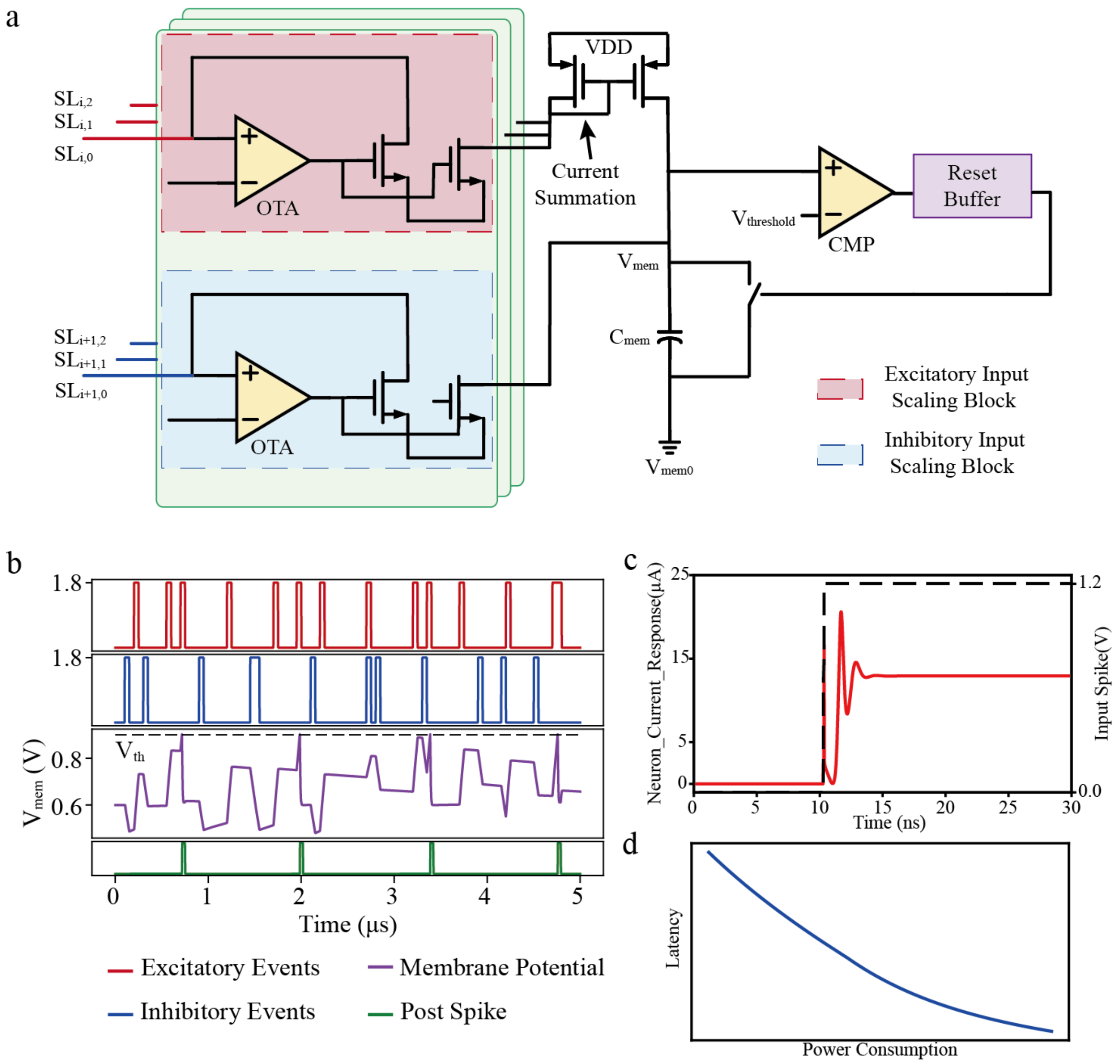

Figure S7: **Overview of the analog LIF neuron.** **a,** Circuit diagram of the analog LIF neuron. The circuit integrates excitatory $SL_{i,k}$) and inhibitory inputs ($SL_{i+1,k}$) through two memristor cells, combined to represent the neuron's external input via differential pairs. These inputs charge the membrane capacitor C_{mem} through a series of transistors, gradually increasing the membrane potential V_{mem}. When V_{mem} reaches a defined threshold, a comparator activates the reset buffer, generating a post-spike signal and resetting V_{mem}. **b,** Simulated temporal response of the analog LIF neuron. Excitatory (red) and inhibitory (blue) events modulate the membrane potential V_{mem} (purple trace). The membrane potential increases with excitatory input and decreases with inhibitory input. When V_{mem} exceeds the threshold V_{th} (dashed line), a post-spike signal (green) is generated, followed by a reset of V_{mem}. This simulation illustrates the neural dynamics of the analog LIF neuron. **c,** Simulated temporal response of the analog LIF neuron. **d,** The trade-off between power and latency in analog circuit.

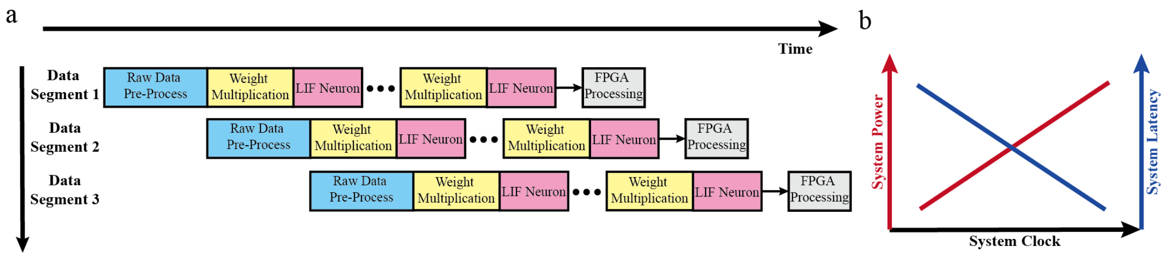

Figure S8: **The latency analysis of the system employing the digital circuit. a,** The latency pipeline of the system. **b,** The trade-off between system power and system latency.

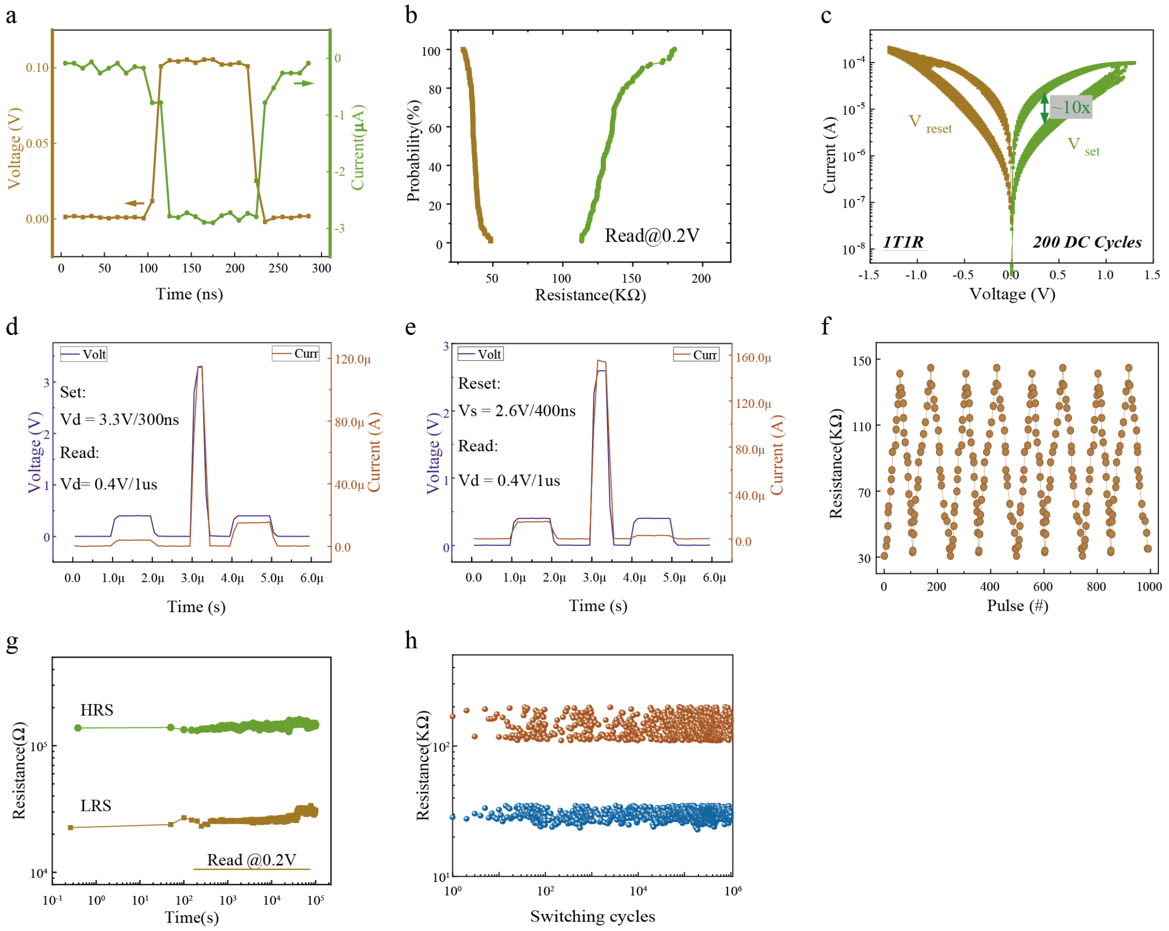

Figure S9: **The electrical characteristics of a 1T1R cell in the 40nm 256Kb RRAM chip.** **a,** Read response of the 1T1R cell. **b,** The high and low resistance states distributions of a 1T1R cell. **c,** The 200-cycle DC current-voltage characteristic of the 1T1R cell. **d,** The voltage and current characteristic of the pulsed SET operation of the 1T1R cell. **e,** The voltage and current characteristic of the pulsed RESET operation of the 1T1R cell. **f,** The incremental analogue programming of the 1T1R cell. **g,** The retention of the 1T1R cell, showing the small noise. **h,** The endurance of the 1T1R cell.

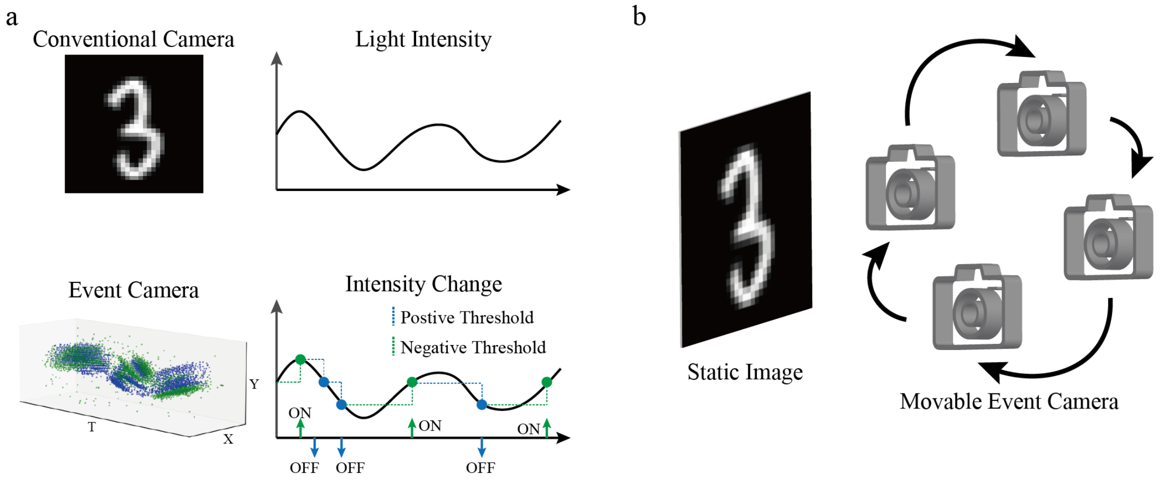

Figure S10: **Illustration of N-MNIST dataset creation using the event camera. a,** Illustration of working mechanism for event camera. The right side shows the light intensity for conventional camera and intensity change on a pixel for event camera. In event cameras, a positive spike ("ON") is generated when pixel intensity exceeds the positive threshold, and a negative spike ("OFF") occurs when it falls below the negative threshold. Some implementations trigger events based on relative intensity changes rather than fixed thresholds, reducing spurious events in varying lighting conditions. **b,** Generation of N-MNIST data. The MNIST image is displayed on a screen, while the event camera is rotated to induce changes in pixel intensity.

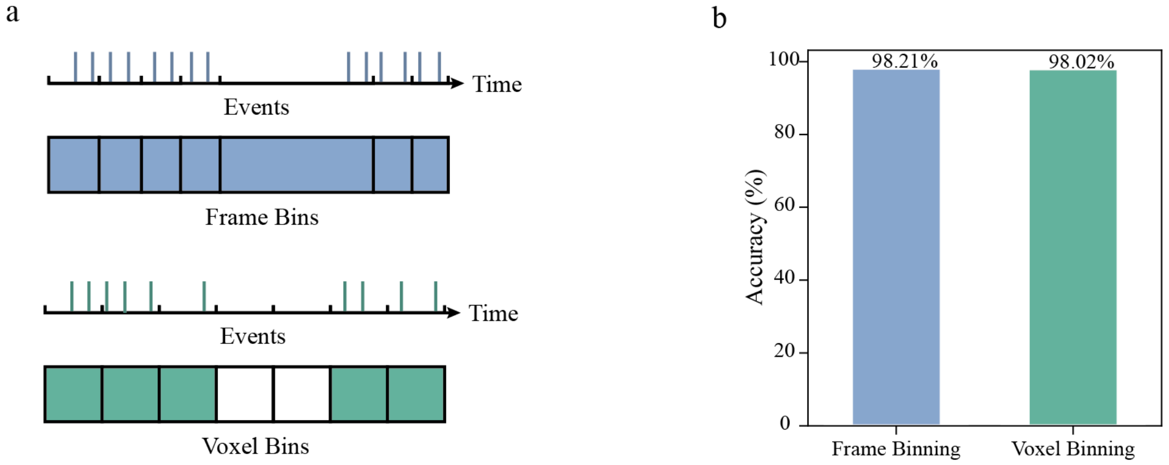

Figure S11: **Overview of event compression methods.** **a,** Schematic representation of frame binning and voxel binning methods. **b,** Comparison of classification accuracy for software-based pruned networks using event data compressed with frame binning and voxel binning methods.

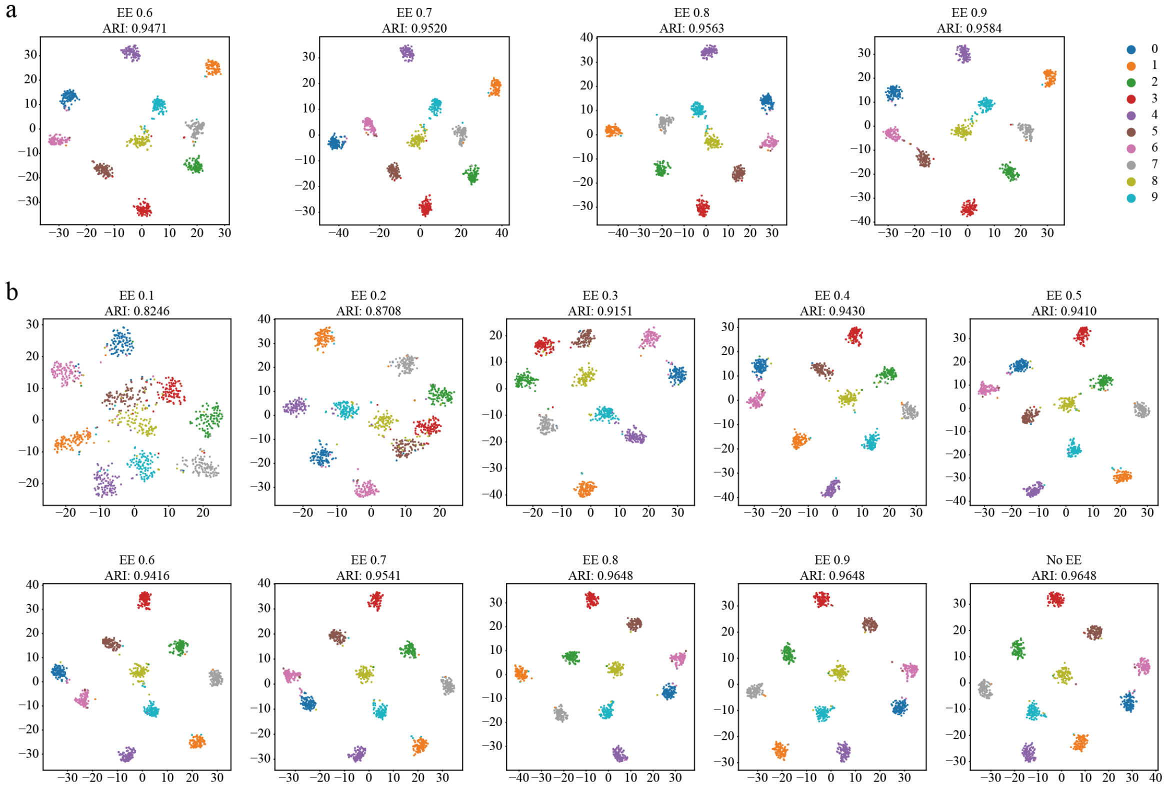

Figure S12: **tSNE visualizations of latent representations at different early stop thresholds on N-MNIST classification.** **a,** tSNE visualizations from PRIME. **b,** tSNE visualizations from software baseline. Colors: ground truth subpopulation labels.

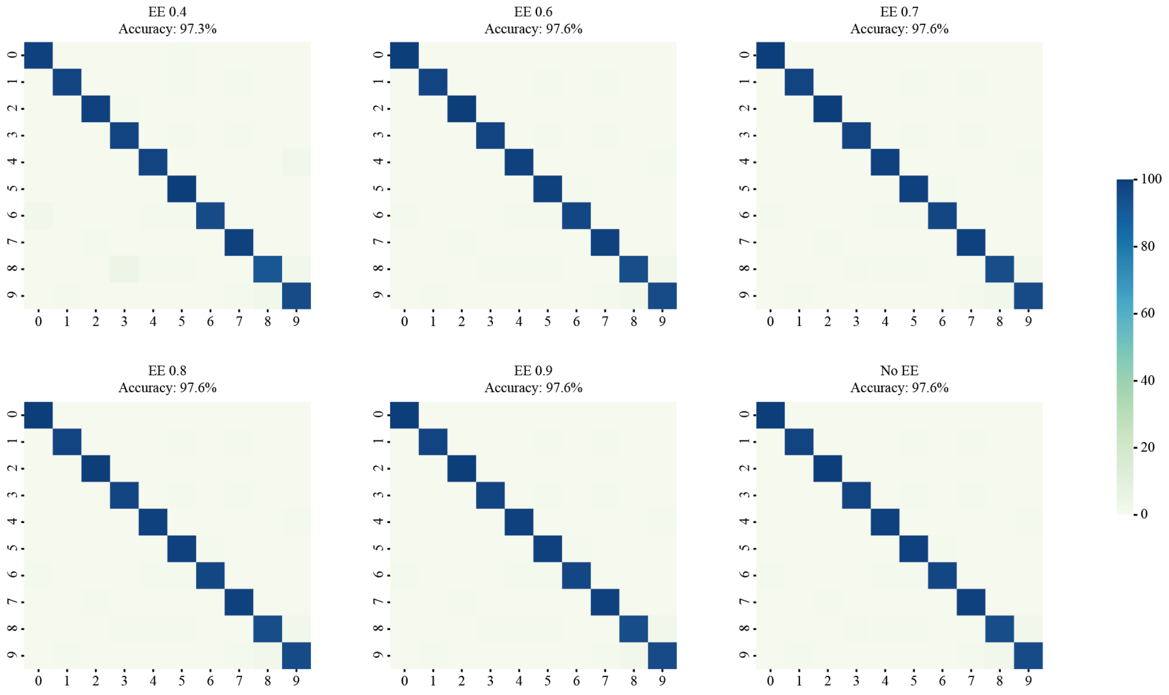

Figure S13: **Confusion matrices and classification accuracy of PRIME at different early stop thresholds.**

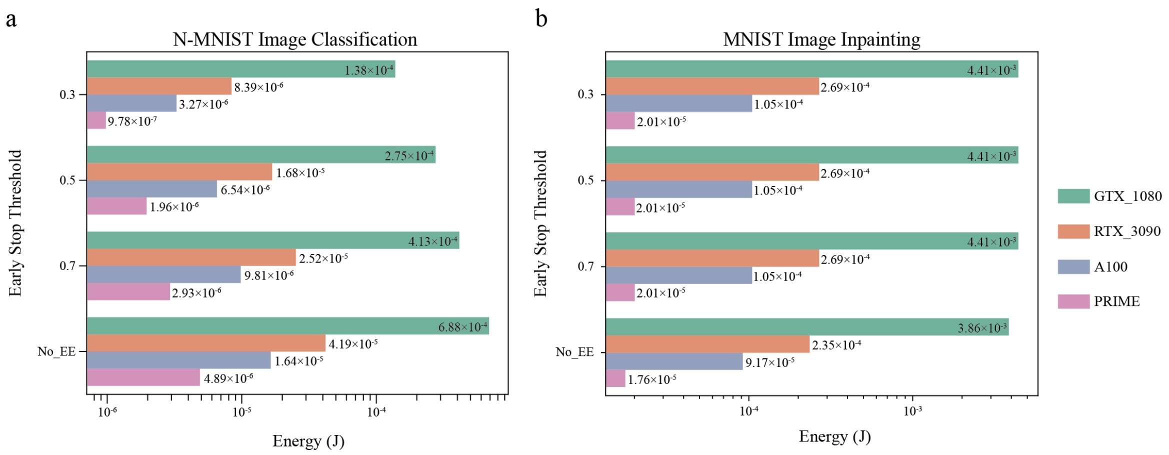

Figure S14: **Comparison of inference energy of a single image on RRAM, GPU 1080, GPU 3090 and GPU A100 at different early stop thresholds.** **a,** Inference energy comparison on N-MNIST classification. **b,** Inference energy comparison on MNIST image inpainting.

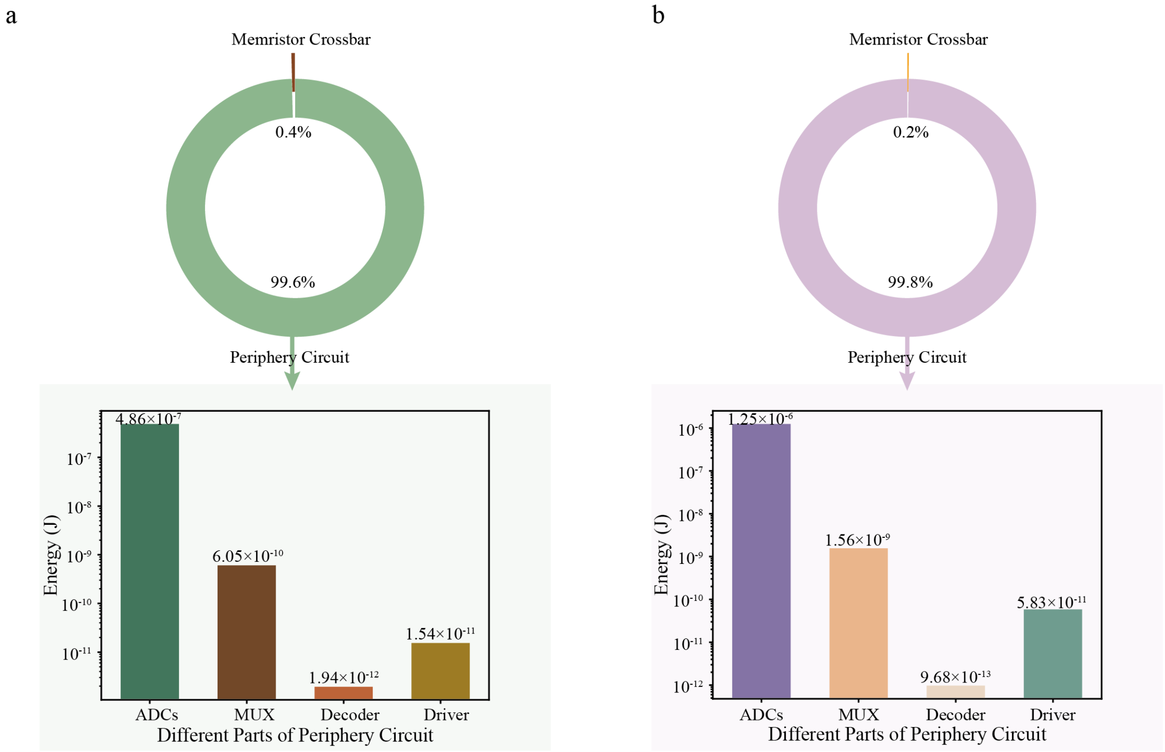

Figure S15: **Energy compositions of our system per timestep. a, N-MNIST image classification.** Energy percentages of two parts of our system (top). Detailed energy compositions of the periphery circuit (bottom). **b, MNIST image inpainting.** Energy percentages of two parts of our system (top). Detailed energy compositions of the periphery circuit (bottom).

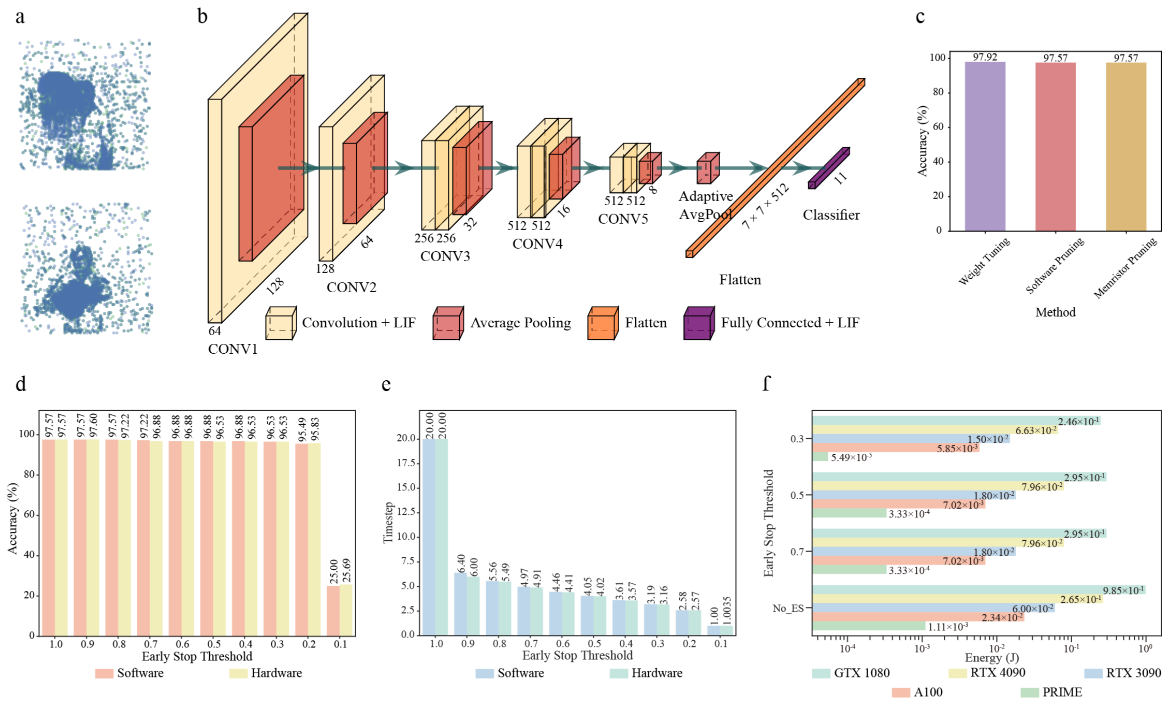

Figure S16: **Experimental image classfication for DVS128Gesture dataset with simulated memristor-based PRIME. a,** Visualizations of the DVS128Gesture neuromorphic dataset, which contains 11 different actions. **b,** Diagram of the spiking VGG-11 architecture (80) used by PRIME for DVS128Gesture classification. **c,** The classification accuracy for SNNs by different optimization methods. **Weight Tuning**: Optimize the weights of software SNN through SLTT (80). **Software Pruning**: Optimize the topology of software SNN, with randomly initialized weights. **Memristor Pruning**: Optimize the topology of memristor-based SNN, where the random weights are produced by memristor programming stochasticity. **d,** The classification accuracy comparisons of hardware PRIME and software baseline at various early stop thresholds. **e,** The dynamic latency (evaluated as the average timesteps of the test data) comparisons of hardware PRIME and software baseline at various early stop thresholds. **f,** Comparison of the inference energy of a single image with PRIME and digital hardware at different early stop thresholds. The former shows a significant energy reduction due to in-memory computing.

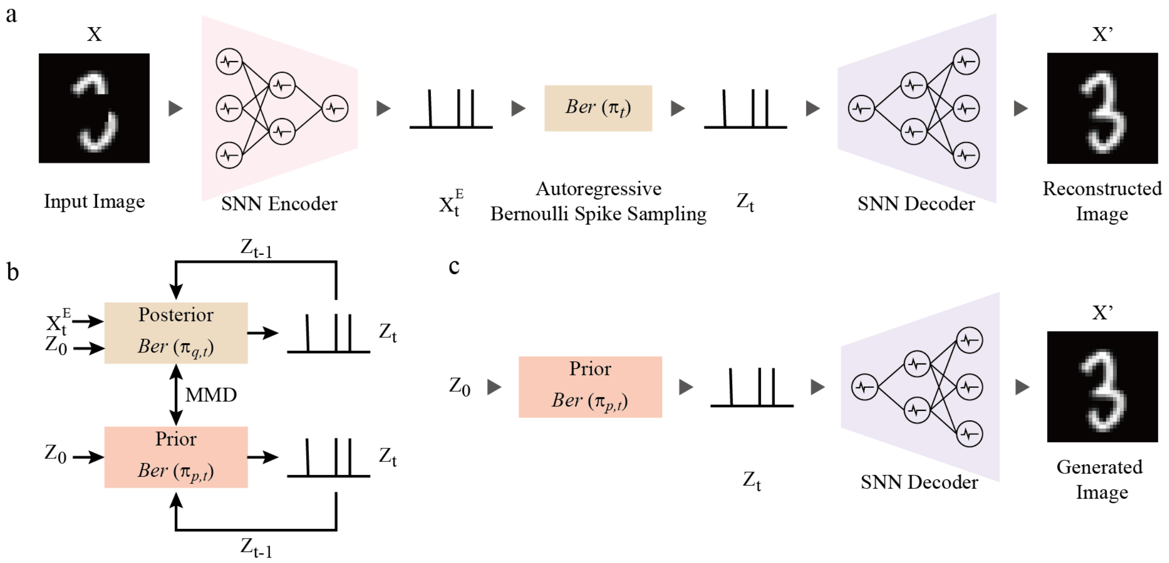

Figure S17: **Overview of FSVAE. a,** Training phase. The input image X is first transformed into spike trains with direct input encoding, and then spike trains are passed through the SNN encoder to obtain $x_{1:t}^E$. When receiving the x_t^E and the previously generated latent variable z_{t-1}, posterior outputs the latent spike z_t incrementally. Then, the SNN Decoder decodes the latent spikes to obtain the reconstructed image x'. **b,** Posterior and prior procedure. The posterior takes the previously generated latent variables z_{t-1} and x_t^E as input and sequentially outputs z_t. In contrast, the prior only takes z_{t-1} as input to generate z_t. Next, the SNN decoder sequentially receives z_t and decodes the spikes to obtain the reconstructed image x'. The MMD loss is used to minimize the prior and posterior probability distributions. **c,** Sampling phase. The image is generated from $z_{1:T}$ sampled in the prior.

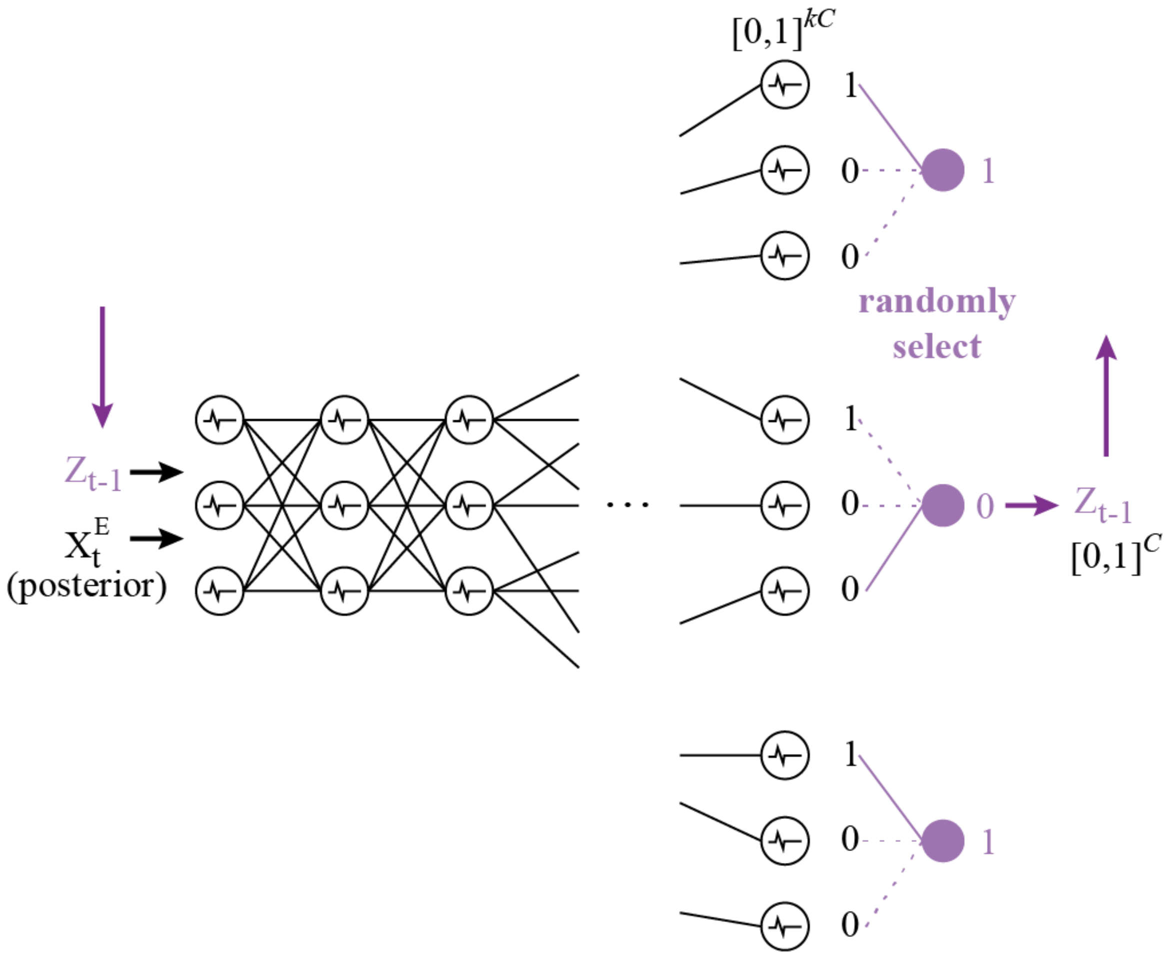

Figure S18: **Autoregressive Bernoulli spike sampling for prior and posterior distributions.** The Bernoulli distribution is realized by randomly selecting C channels from kC channels of the posterior or prior network output. The input of posterior network is z_{t-1} and x_t^E. The input of prior network is z_{t-1}.

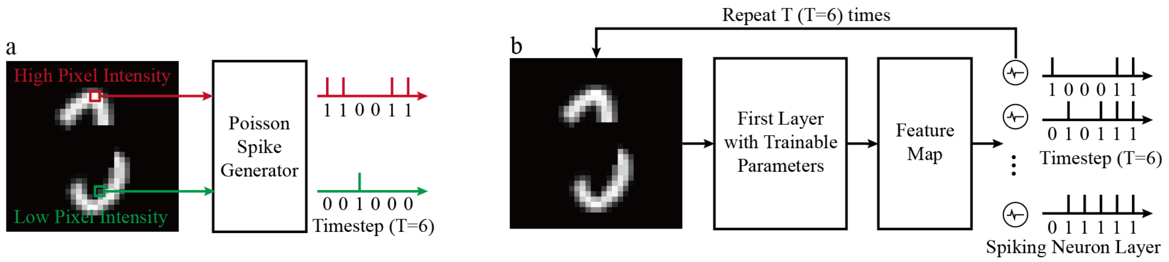

Figure S19: **Schematic diagram of rate coding and direct coding. a,** Rate coding. Rate coding considers each input pixel intensity to be proportional to the firing rate of Poisson-distributed spike trains. **b,** Direct coding. Direct coding uses the first layer (FC layer or Convolutional layer) of the network as a coding layer, and transforms the input into spikes.

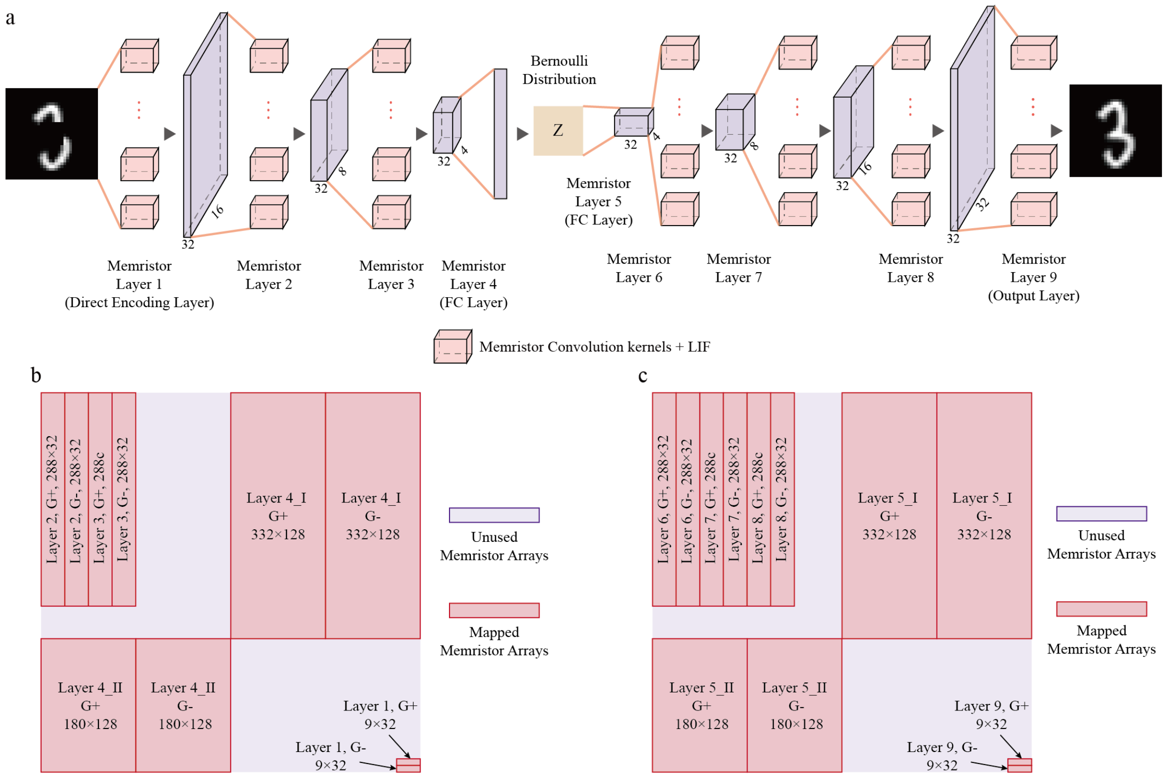

Figure S20: **Deployment of spikingVAE on memristor crossbar array. a,** Diagram of the spikingVAE used in PRIME for MNIST inpainting. The encoders and decoders are physically implemented on memristor array, and the autoregressive Bernoulli spiking sampling is running on GPUs. **b,** Mapped arrays of encoder layers on memristor crossbar array. **c,** Mapped arrays of decoder layers on memristor crossbar array.

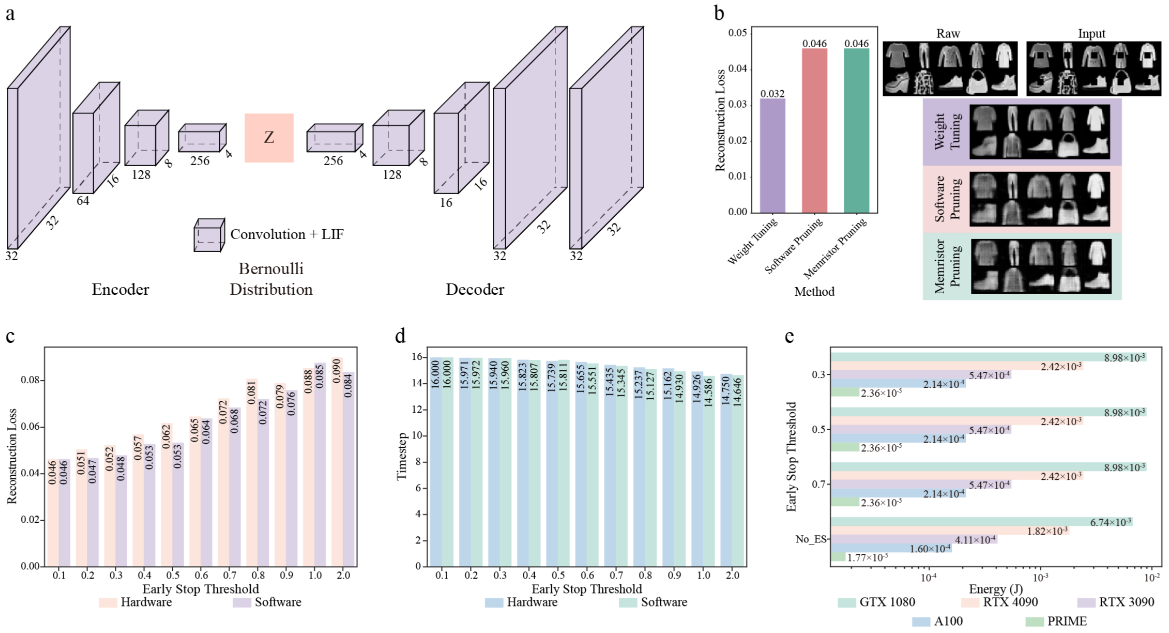

Figure S21: **Experimental image inpainting of Fashion-MNIST dataset with simulated memristor-based PRIME. a,** Diagram of the spiking VAE in PRIME with expanded encoder and decoder networks. **b,** The reconstruction loss for SNNs optimized by different methods (left). **Weight Tuning**: Optimize the weights of software SNN through STBP. **Software Pruning**: Optimize the topology of software SNN with randomly initialized weights. **Memristor Pruning**: Optimize the topology of memristor-based SNN, where the random weights are produced by memristor programming stochasticity. The raw, input, and reconstructed images by different methods (right). **c,** The reconstruction loss comparisons of hardware PRIME and software baseline at various early stop thresholds on Fashion-MNIST. **d,** The dynamic timestep (evaluated as the average timesteps of the test data) comparisons of hardware PRIME and software baseline at various early stop thresholds on Fashion-MNIST. **e,** Comparison of inference energy with PRIME and digital hardwares at different early stop thresholds.

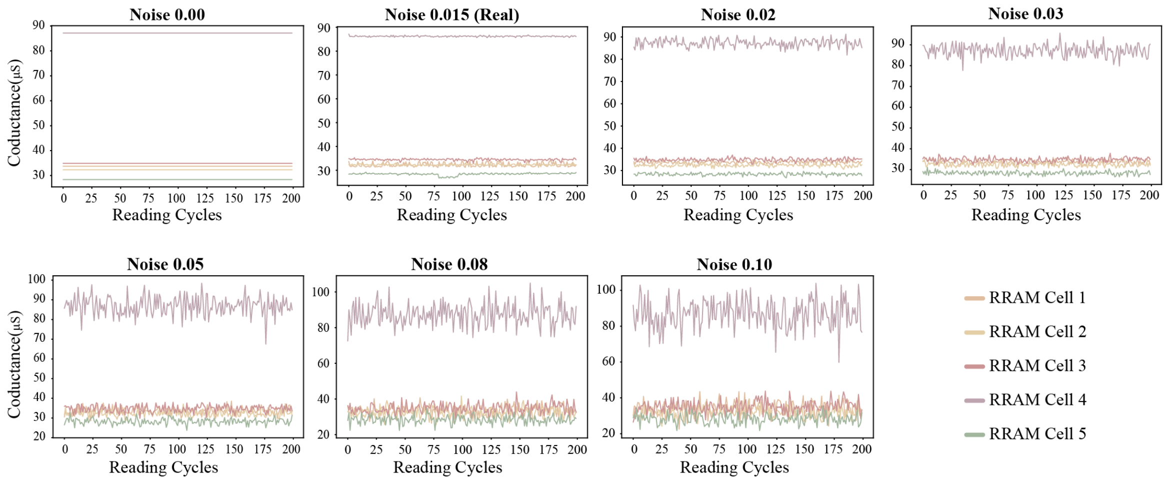

Figure S22: **The conductance perturbations of 5 randomly selected memristors with 200 read cycles over seven levels of noise.**

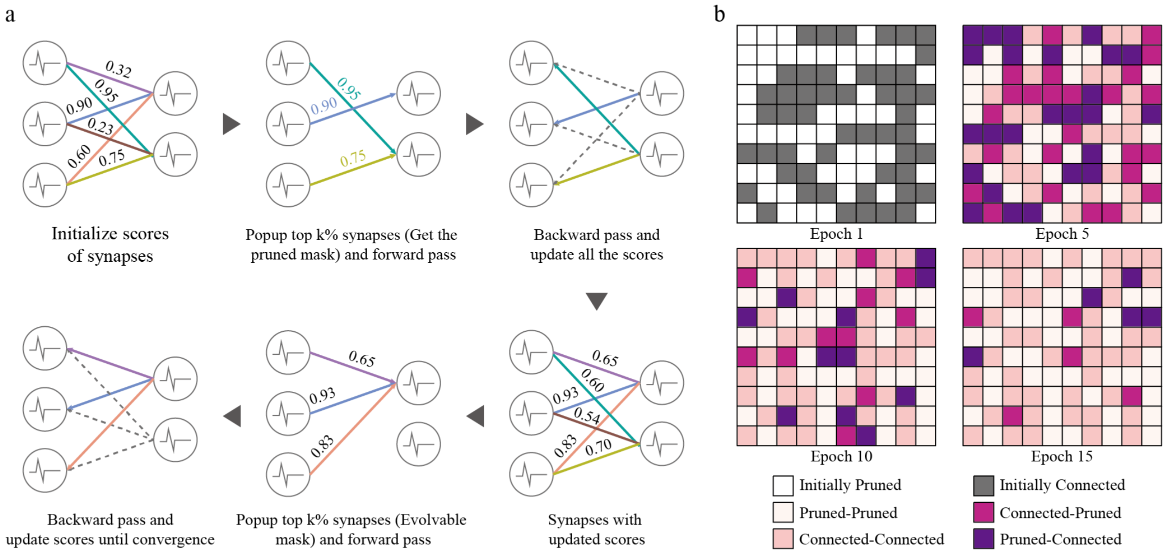

Figure S23: **Overview of topology optimization algorithm. a,** The procedure of score-based topology optimization algorithm. Initially, each random synaptic weight w is assigned a score s, reflecting its importance. Synapses with the top k% scores are retained, while others are pruned to form a subnet. The loss is calculated using this subnet and backpropagated through the entire network to optimize the scores. Then, some pruned synapses with updated scores regrow, and some retained synapses are pruned. This process is iterated until convergence, yielding the optimal subnet. **b,** Evolution of a portion of the pruning mask during training on the N-MNIST classification task. **Pruned-Connected** boxes denote regrowing synapses, while **Connected-Pruned** boxes indicate retained synapses that have been pruned. **Connected-Connected** and **Pruned-Pruned** boxes represent synapses whose connection states remain unchanged.

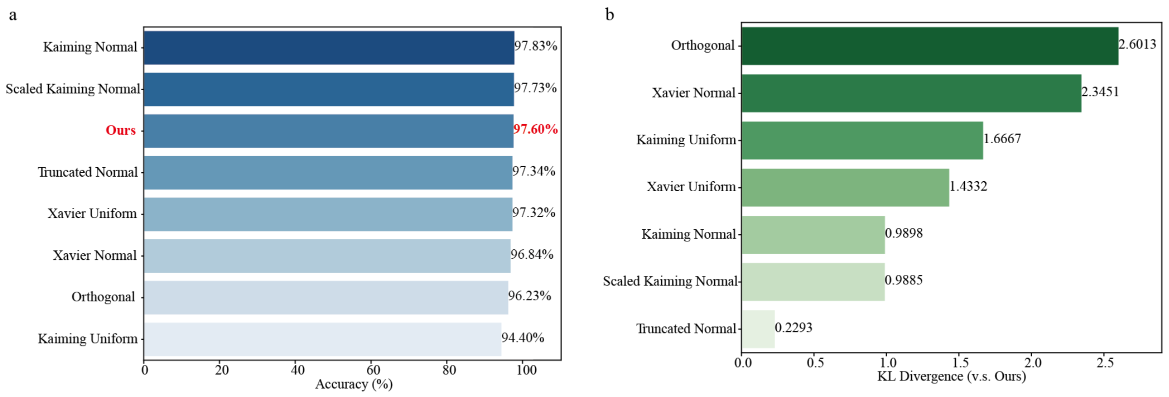

Figure S24: **Experimental image classification for N-MNIST with pruned networks initialized using different distributions. a,** Comparison of classification accuracy for pruned networks initialized using different distributions. **b,** KL divergence between the distribution of memristor-based weights and those of other initialization distributions.

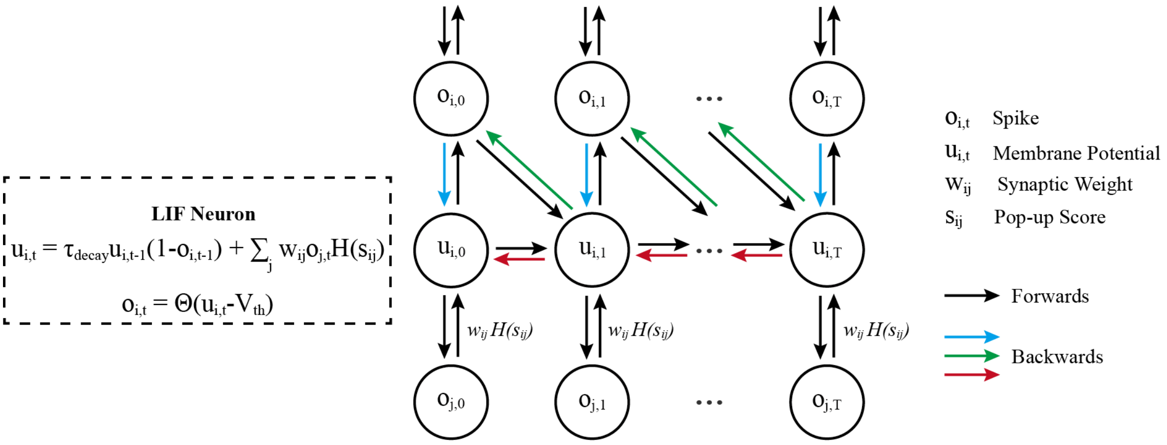

Figure S25: **Illustration of the forward and backward of a spiking neuron as used for BPTT for a sequence $t = 0, ..., T$.**

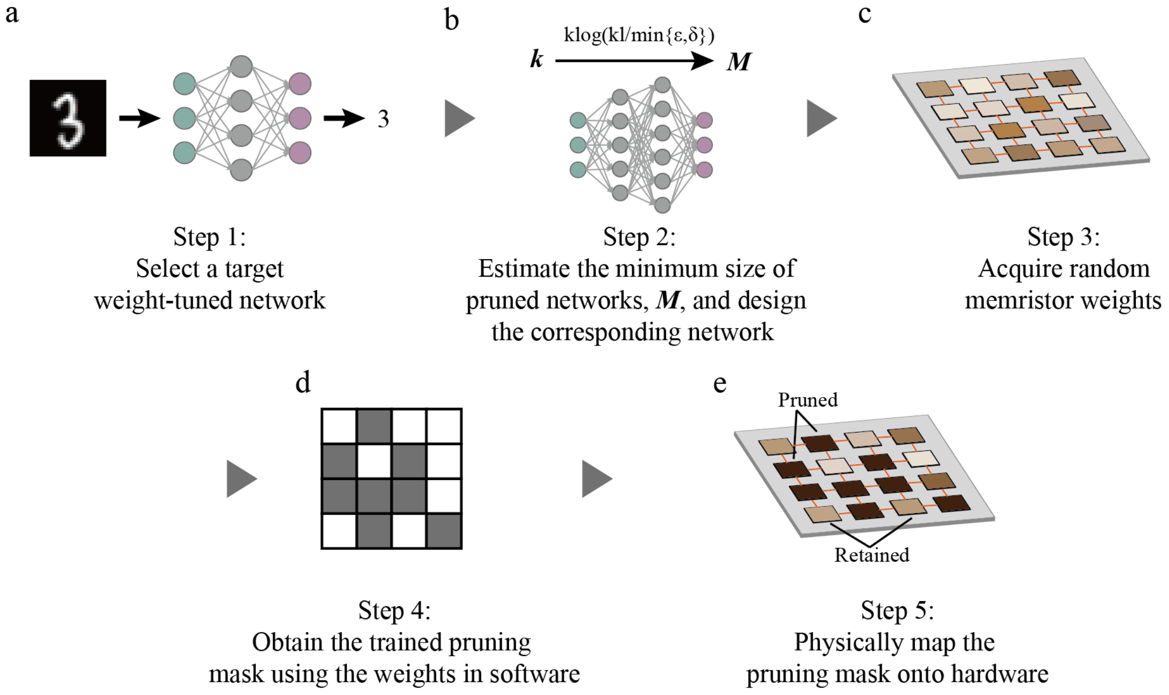

Figure S26: **Steps of determining the appropriate memristor array size.** **a,** Select a target weight-tuned network with a minimal number of parameters, k. **b,** Estimate the required network size, M, for pruning based on the theoretical restrictions, i.e. CNN, $k \log\left(\frac{kl}{\min\{\epsilon,\delta\}}\right)$. And design the over-parameterized random networks by deepening or widening the weight-tuned network to approximate the estimated network size M. **c,** Acquire random memristor weights based on the estimated M. **d,** Obtain the pruning mask for the random memristor weights, trained in software. **e,** Physically map the pruning mask onto the random memristor array.

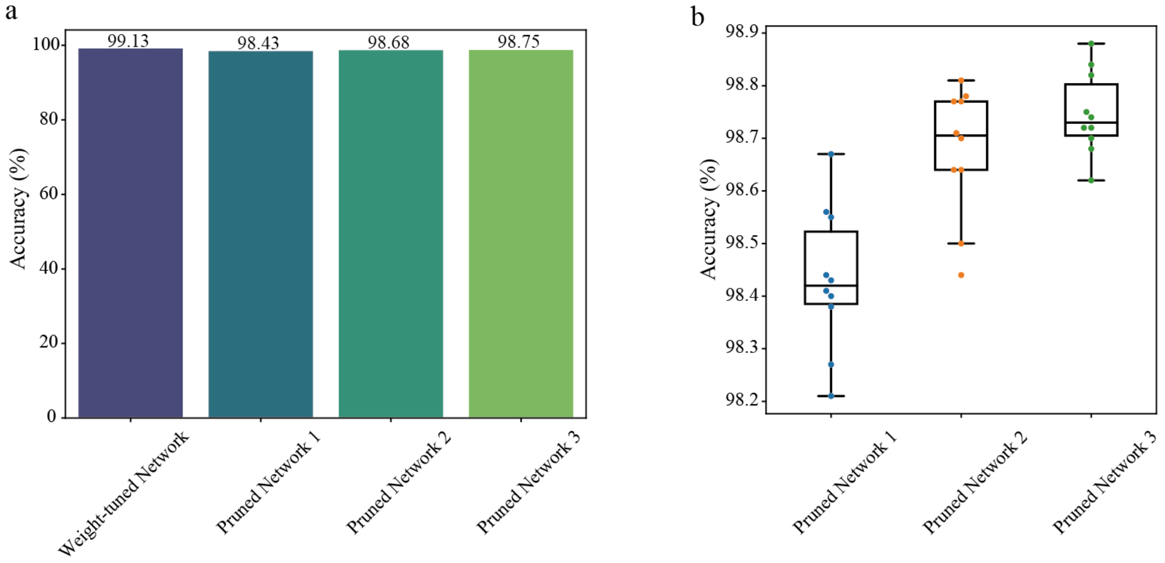

Figure S27: **Experimental image classification for N-MNIST dataset on various network architectures. a,** The classification accuracy comparisons of pruned networks with different sizes. **b,** Box plots of accuracy across 10 trials in three pruned networks. Box plot: median (center line), interquartile range (box) and data range (whiskers). **Weight-tuned Network**: Optimize the weights of the well-performing network in software. **Pruned Network N**: Optimize the topology of networks (N = 1,2,3) with randomly initialized memristor-based weights.

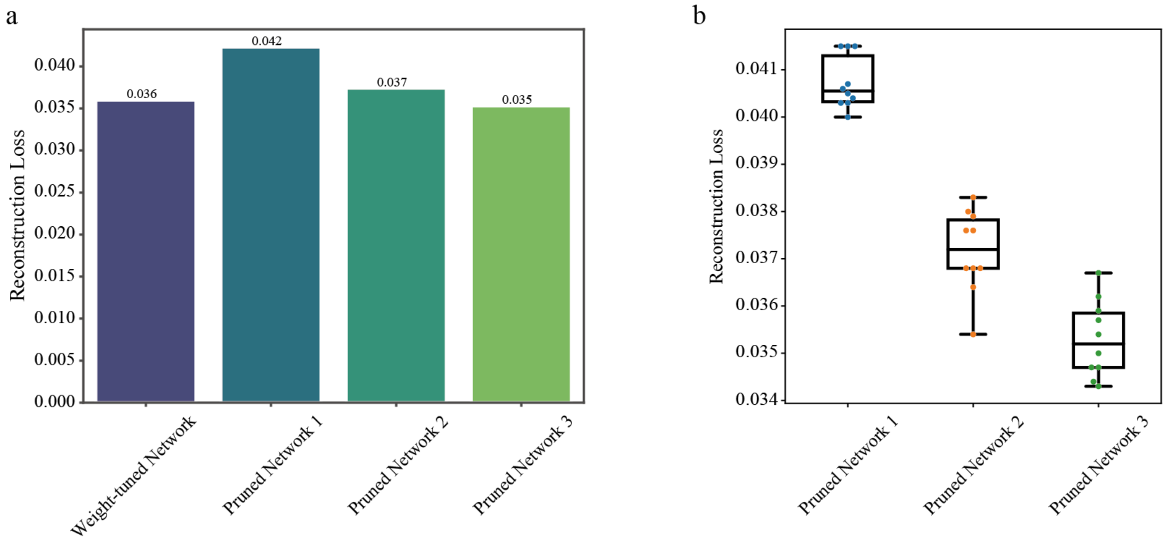

Figure S28: **Experimental image inpainting for MNIST dataset on various network architectures. a,** The reconstruction loss comparisons of pruned networks of various sizes. **b,** Box plots of reconstruction loss across 10 trials in three pruned networks. Box plot: median (center line), interquartile range (box) and data range (whiskers). **Weight-tuned Network**: Optimize the weights of the well-performing network in software. **Pruned Network N**: Optimize the topology of networks (N = 1,2,3) with randomly initialized memristor-based weights.

S3. Supplementary Table

Table S1: Publications about analog circuit or asynchronous input processing in SNNs

	Publication	Hardware	Dataset	Compression
1	NC 2024 (102)	Analog Neuromorphic Chip	Event-based SHD	Time binning
2	NC 2024 (103)	Asynchronous Neuromorphic Chip	DVS-Gesture	Frame binning
3	NC 2024 (67)	Analog Neuromorphic Chip	Event-based SHD	Time binning
4	PNAS 2022 (104)	Analog Neuromorhphic Chip	Event-based SHD	Time binning
5	TETC 2023 (105)	Analog Neuromorphic Chip	DVS-Gesture	Time binning
6	Frontiers in Neuroinformatics 2023 (88)	Asynchronous Analog Circuit	N-MNIST/ DVS-Gesture	Frame binning
7	ICASSP 2024 (87)	Asynchronous Analog Circuit	N-MNIST/ DVS-Gesture	Frame binning
8	AICAS 2020 (106)	Asynchronous input	DVS-Gesture	Time Binning
9	JETCAS 2019 (82)	Asynchronous input	DVS-Gesture	Time binning

Table S2: Comparison of Pruning Methods for SNNs on N-MNIST

	**Goal**	**Pruning Method**	**Sparsity**	**Acc**	**Params** (Example Network)	**Resist.** [*]
**N-MNIST**						
Ours	Optimize Network	Unstructured synapse pruning	50 % Random Weights	97.60%	2 Conv 0.021M	High
Grad R [1]	Network Compression	Unstructured synapse pruning	75% Tuned Weights	98.56%	2 Conv 2 FC 0.149M	Low
ADMM [2]	Network Compression	Unstructured synapse pruning	50% Tuned Weights	98.34%	2 Conv 2 FC 0.031M	Low
SD-SNN [3]	Network Compression	Unstructured neuron pruning and synapse regeneration	58.62% Tuned Weights	99.53%	2 Conv 2 FC 0.246M	Low
SNDP [4]	Network Compression	Unstructured neuron pruning	87.32% Tuned Weights	95.24%	2 Conv 2 FC 0.068M	Low
DPAP [5]	Network Compression	Unstructured neuron and synapse pruning	50.41% Tuned Weights	99.55%	2 Conv 2 FC 0.296M	Low

[1] IJCAI 2021 (89) [2] TNNLS 2021 (91) [3] Information Sciences 2025 (92) [4] ICME 2024 (90) [5] TPAMI 2024 (93)

[*] Resistance to Memristor Programming Stochasticity

Table S3: Comparison of Pruning Methods for SNNs on DVS-Gesture

	**Goal**	**Pruning Method**	**Sparsity**	**Acc**	**Params** (Example Net)	**Resist.** *
**DVS-Gesture**						
Ours	Optimize Network	Unstructured synapse pruning	50 % Random Weights	97.57%	VGG-11 6.662M	High
Deep R [1]	Network Compression	Unstructured neuron and synapse pruning	75.00% Tuned Weights	81.23%	2 Conv 2 FC 3.073M	Low
Grad R [1]	Network Compression	Unstructured synapse pruning	75% Tuned Weights	91.95%	2 Conv 2 FC 3.073M	Low
SCCD [2]	Network Compression	Structured channel pruning	15.10% Tuned Weights	95.49%	Resnet-20 0.14M	Low
SD-SNN [3]	Network Compression	Unstructured neuron pruning and synapse regeneration	55.50% Tuned Weights	98.20%	2 Conv 2 FC 5.470M	Low
DPAP [4]	Network Compression	Unstructured neuron and synapse pruning	64.03% Tuned Weights	98.56%	2 Conv 2 FC 4.422M	Low
Network Slimming [5]	Network Compression	Unstructured neuron and structured channel pruning	20.00% Weight Tuning	94.79%	VGG-13 5.350M	Low
BinWSNN [6]	Network Compression	Structured patch pruning	50.00% Weight Tuning	91.70%	VGG-9 3.885M	Low

[1] IJCAI 2021 (89), [2] Applied Intelligence 2023 (94), [3] Information Sciences 2025 (92) [4] TPAMI 2024 (93) [5] TAI 2024 (95) [6] Arxiv 2023 (96)

* Resistance to Memristor Programming Stochasticity

Table S4: The sizes of convolutional layers and fully-connected layers in spikingVAE and the corresponding memristor array sizes

	Conv Layer[1]	FC Layer[2]	Params.	Memristor Array ($\times$2 means G^+ and G^-)
Layer 1	1 32 3	–	288	9×32×2
Layer 2	32 32 3	–	9216	288×32×2
Layer 3	32 32 3	–	9216	288×32×2
Layer 4	–	128	65536	512×128×2
Layer 5	–	512	65536	128×512×2
Layer 6	32 32 3	–	9216	288×32×2
Layer 7	32 32 3	–	9216	288×32×2
Layer 8	32 32 3	–	9216	288×32×2
Layer 9	32 1 3	–	288	9×32×2

[1] Conv Layer: Input Channel, Output Channel, Kernel Size, [2] FC Layer: Dimension of this layer

Table S5: Training Settings used in PRIME on N-MNIST Classification.

Variable	Value	Variable	Value
Batch Size	128	Learning Rate	0.001
Epoch	14	Weight Decay	0.0005
Timestep	10	Optimizer	Adam
Sparsity	0.5	Loss	CrossEntropy

Table S6: Training Settings used in PRIME on DVS128Gesture Classification.

Variable	Value	Variable	Value
Batch Size	16	Learning Rate	0.1
Epoch	300	Weight Decay	0.0005
Timestep	20	Optimizer	Adam
Sparsity	0.5	Loss	CrossEntropy

Table S7: Training Settings used in PRIME on Image (MNIST, Fashion-MNIST) Inpainting.

Variable	Value	Variable	Value
Batch Size	250	Learning Rate	0.001
Epoch	150	Weight Decay	0.001
Timestep	16	Optimizer	AdamW
Sparsity	0.5	Loss	MSE+mmd

Table S8: Default parameters used in PRIME on N-MNIST Classification.

Variable	Value
Membrane Decay	0.2
Threshold Potential	0.5 / 0.2*
Resting Potential	0
Pseudo-derivative Hyperparameter a	1.0

* Fully-Connected Layer / Convolutional Layer.

Table S9: Default parameters used in PRIME on DVS128Gesture Classification.

Variable	Value
Membrane Decay	0.9
Threshold Potential	1.0
Resting Potential	0
Pseudo-derivative Hyperparameter a	1.0

Table S10: Default parameters used in PRIME on Image (MNIST, Fashion-MNIST) Inpainting.

Variable	Value
Membrane Decay	0.25
Threshold Potential	0.2
Resting Potential	0
Pseudo-derivative Hyperparameter a	1.0

Table S11: Pruned networks corresponding to different ϵ and δ values in N-MNIST classification experiments

	ϵ	δ	$k \log \frac{kl}{\min\{\epsilon,\delta\}}$	Conv Layers	Param.
Original Network	–	–	–	2, 12, 5<br>12, 64, 5	42,840
Pruned Network 1	0.05<br>0.05	0.05<br>0.05	267,060	2, 12, 5<br>12, 64, 5<br>64, 64, 5<br>64, 80, 5	257,400
Pruned Network 2	0.01<br>0.05	0.05<br>0.01	297,004	2, 12, 5<br>12, 64, 5<br>64, 64, 5<br>64, 100, 5	291,200
Pruned Network 3	0.001<br>0.05	0.05<br>0.001	339,844	2, 12, 5<br>12, 64, 5<br>64, 64, 5<br>64, 128, 5	338,520

Table S12: Pruned networks corresponding to different ϵ and δ values in MNIST inpainting experiments

	ϵ	δ	$k \log \frac{kl}{\min\{\epsilon,\delta\}}$	Encoder	Decoder	Param.
Original Network	–	–	–	1, 32, 3<br>32, 32, 3<br>32, 32, 3<br>128	512<br>32, 32, 3<br>32, 32, 3<br>32, 32, 3<br>32, 1, 3	84,256
Pruned Network 1	0.1<br>0.1	0.1<br>0.1	539,467	1, 32, 3<br>32, 64, 5<br>64, 128, 5<br>128, 256, 3<br>128	512<br>256, 128, 3<br>128, 64, 3<br>64, 32, 3<br>32, 32, 3<br>32, 1, 3	518,432
Pruned Network 2	0.001<br>0.1	0.1<br>0.001	707,979	1, 64, 3<br>64, 64, 3<br>64, 256, 3<br>128	512<br>256, 64, 3<br>64, 64, 3<br>64, 32, 3<br>32, 1, 3	709,184
Pruned Network 3	0.0001<br>0.1	0.1<br>0.0001	792,235	1, 32, 3<br>32, 128, 5<br>128, 256, 5<br>128	512<br>256, 128, 3<br>128, 32, 3<br>32, 32, 3<br>32, 1, 3	856,352

Table S13: Cost Estimation and Comparison between SRAM and Memristor, where f^2 Denotes Feature Size of the Process Node.

	SRAM	Memristor
Structure	6-12T	1T1R
Unit Cell Area 1	$> 120f^2$	4-14 f^2
Normalized Array Area on N-MNIST	1^*	0.027^{**}
Application	High-speed caches, In-memory computing on cloud	In-memory computing on edge

* The estimated SRAM chip area is based on original network with 16 bit tuned weights, using $120f^2$ unit area.

** The estimated RRAM chip area is based on Pruned Network 1 with random weights, using $4f^2$ unit area.

Table S14: Comparison of PRIME with other models in generation performance on MNIST Dataset. Smaller values of Fréchet Inception Distance (FID) indicate superior generation performance.

Method	EE Threshold	FID(↓)
Memristor Pruning	0.1	201.8331
Memristor Pruning	1.0	202.0256
Software Pruning	0.1	174.0147
Software Pruning	1.0	174.8826
Weight Tuning	0.1	145.1963
Weight Tuning	1.0	146.813

Table S15: Energy consumption breakdown of the system per timestep.

$E_{RRAM\ Cell}$ = 2.85e-15J<br>E_{ADC} = 1.13e-11J<br>E_{Mux} = 14.1e-15J<br>$E_{Decoder}$ = 0.42e-15J<br>E_{Driver} = 0.18e-15J	Systems	Weight Size[1)]<br>$(in \times out \times k_h \times k_w)$<br>$(height \times width)$	Param.	Ops.[2)]	RRAM Crossbar[3)]	ADC[4)]	MUX[5)]	Decoder[6)]	Driver[7)]	Total[8)]
Image Classification<br>(Full T = 10)	RTX 4090	$2 \times 12 \times 5 \times 5$<br>$12 \times 64 \times 5 \times 5$<br>$2304 \times 10$	42.8K	3.4M	–	–	–	–	–	18.54μJ
	RTX3090				–	–	–	–	–	4.193μJ
	GTX1080				–	–	–	–	–	68.809μJ
	A100				–	–	–	–	–	1.636μJ
	Our System				2.106nJ	0.486μJ	0.605nJ	1.942pJ	15.422pJ	0.489μJ
Image Inpainting<br>(Full T = 16)	RTX 4090	$1 \times 32 \times 3 \times 3$<br>$32 \times 32 \times 3 \times 3$<br>$32 \times 32 \times 3 \times 3$<br>$512 \times 128$<br>Bernoulli Sampling<br>$128 \times 512$<br>$32 \times 32 \times 3 \times 3$<br>$32 \times 32 \times 3 \times 3$<br>$32 \times 32 \times 3 \times 3$<br>$32 \times 1 \times 3 \times 3$	162.1K	13.6M	–	–	–	–	–	74.24μJ
	RTX3090				–	–	–	–	–	16.79μJ
	GTX1080				–	–	–	–	–	275.52μJ
	A100				–	–	–	–	–	6.55μJ
	Our System				2.25nJ	1.25μJ	1.56nJ	0.97pJ	58.35pJ	1.26μJ

[1] The neural network architectures used in different tasks.

[2] "Ops" denotes the quantity of floating-point operations executed in a single inference, calculated as follows:

Convolutional Layer

$$Ops. = out_channel \times in_channel \times kernel_h \times kernel_w \times h \times w, \tag{S44}$$

where $out_channel$, $in_channel$, $kernel_h$, $kernel_w$, h, and w denote the convolutional layer's out channels, in channels, kernel's height and width, and output feature maps' height and width, respectively.

FC Layer

$$Ops. = w_h \times w_w, \tag{S45}$$

where w_h and w_w denote the weight's height and width of fully connected layers, respectively.

[3] The energy consumption of the single working RRAM cell is evaluated as follows:

$$E_{RRAM\ \ Cell} = t_{read} \times V^2 \times \overline{G}, \tag{S46}$$

where t_{read}, V, and $\overline{G}$ denote the cell's read response, read voltage, and the average conductance of RRAM cell. The energy consumption of the resistive memory crossbar is evaluated as follows:

$$E_{Crossbar} = (R \times E_{RRAM\ \ Cell}) \times w_h \times w_w, \tag{S47}$$

where R denote the activated ratio of RRAM cells for the sparse spikes emitted by spiking neurons, w_h, and w_w denote the height, and width of the weight matrix.

[4] The energy consumption is calculated as follows

$$E_{ADC} = Sampling\ \ Rate \times ADC\ \ power, \tag{S48}$$

$$ADC = E_{ADC} \times w_w. \tag{S49}$$

[5] The energy consumption of the multiplexer is evaluated as follows:

$$Mux = E_{Mux} \times w_w. \tag{S50}$$

[6] The energy consumption of the decoder is evaluated as follows:

$$Decoder = E_{Decoder} \times w_w. \tag{S51}$$

[7] The energy consumption of the drivers is evaluated as follows:

$$Drivers = E_{Driver} \times w_h \times w_w. \tag{S52}$$

[8] The total energy consumption is evaluated as follows:

Our system

$$Total = E_{Crossbar} + 2 \times (E_{ADC} + E_{Mux} + E_{Decoder} + E_{Drivers}), \tag{S53}$$

where 2× is associated with differential pairs.

GPUs

$$Total = Ops. \times \frac{Power\ \ Draw}{FP16\ \ Theoretical\ \ Performance}. \tag{S54}$$

Here, the power draw and FP16 theoretical performance of the NVIDIA GeForce RTX 4090, 3090, 1080, and A100 are 450W and 82.58TFLOPS; 350W and 284TFLOPS; 180W and 8.9TFLOPS; 300W and 624TFLOPS.

REFERENCES AND NOTES

1. Y. LeCun, Y. Bengio, G. Hinton, Deep learning. *Nature* **521**, 436–444 (2015).

2. D. Kuzum, R. G. Jeyasingh, B. Lee, H.-S. P. Wong, Nanoelectronic programmable synapses based on phase change materials for brain-inspired computing. *Nano Lett.* **12**, 2179–2186 (2012).

3. T. Zhang, K. Yang, X. Xu, Y. Cai, Y. Yang, R. Huang, Memristive devices and networks for brain-inspired computing. *PSS RRL* **13**, 1900029 (2019).

4. Y. Chang, X. Wang, J. Wang, Y. Wu, L. Yang, K. Zhu, H. Chen, X. Yi, C. Wang, Y. Wang, W. Ye, Y. Zhang, Y. Chang, P. S. Yu, Q. Yang, X. Xing, A survey on evaluation of large language models. *ACM Trans. Intell. Syst. Technol.* **15**, 39 (2024).

5. W. X. Zhao, K. Zhou, J. Li, T. Tang, X. Wang, Y. Hou, Y. Min, B. Zhang, J. Zhang, Z. Dong, D. Yifan, C. Yang, Y. Chen, Z. Chen, J. Jiang, R. Ren, Y. Li, X. Tang, Z. Liu, P. Liu, J.-Y Nie, J.-R Wen, A survey of large language models. arXiv:2303.18223 (2023).

6. T. Brooks, B. Peebles, C. Holmes, W. DePue, Y. Guo, L. Jing, D. Schnurr, J. Taylor, T. Luhman, E. Luhman, C. Ng, R. Wang, A. Ramesh, Video generation models as world simulators. Technical Report (2024); https://openai.com/research/video-generation-models-as-world-simulators.

7. S. Kumar, X. Wang, J. P. Strachan, Y. Yang, W. D. Lu, Dynamical memristors for higher-complexity neuromorphic computing. *Nat. Rev. Mat.* **7**, 575–591 (2022).

8. J. J. Yang, D. B. Strukov, D. R. Stewart, Memristive devices for computing. *Nat. Nanotechnol.* **8**, 13–24 (2013).

9. D. Ielmini, H.-S. P. Wong, In-memory computing with resistive switching devices. *Nat. Electron.* **1**, 333–343 (2018).

10. H. Ning, Z. Yu, Q. Zhang, H. Wen, B. Gao, Y. Mao, Y. Li, Y. Zhou, Y. Zhou, J. Chen, L. Liu, W. Wang, T. Li, Y. Li, W. Meng, W. Li, Y. Li, H. Qiu, Y. Shi, Y. Chai, H. Wu, X. Wang, An

in-memory computing architecture based on a duplex two-dimensional material structure for in situ machine learning. *Nat. Nanotechnol.* **18**, 493–500 (2023).

11. M. A. Zidan, J. P. Strachan, W. D. Lu, The future of electronics based on memristive systems. *Nat. Electron.* **1**, 22–29 (2018).

12. P. Lennie, The cost of cortical computation. *Curr. Biol.* **13**, 493–497 (2003).

13. M. G. Mattar, M. Lengyel, Planning in the brain. *Neuron* **110**, 914–934 (2022).

14. J. Snider, D. Lee, H. Poizner, S. Gepshtein, Prospective optimization with limited resources. *PLoS Comput. Biol.* **11**, e1004501 (2015).

15. M. Keramati, P. Smittenaar, R. J. Dolan, P. Dayan, Adaptive integration of habits into depth-limited planning defines a habitual-goal–directed spectrum. *Proc. Natl. Acad. Sci. U.S.A.* **113**, 12868–12873 (2016).

16. B. Van Opheusden, G. Galbiati, Z. Bnaya, Y. Li, W. J. Ma, "A computational model for decision tree search," in *Proceedings of the Annual Meeting of the Cognitive Science Society* (UC Merced, 2017), vol. 39; https://escholarship.org/uc/item/0v94n7cd.

17. R. Llinás, H. Jahnsen, Electrophysiology of mammalian thalamic neurones in vitro. *Nature* **297**, 406–408 (1982).

18. G. Hahn, A. Ponce-Alvarez, G. Deco, A. Aertsen, A. Kumar, Portraits of communication in neuronal networks. *Nat. Rev. Neurosci.* **20**, 117–127 (2019).

19. S. Woźniak, A. Pantazi, T. Bohnstingl, E. Eleftheriou, Deep learning incorporating biologically inspired neural dynamics and in-memory computing. *Nat. Mach. Intell.* **2**, 325–336 (2020).

20. A. Mehonic, A. J. Kenyon, Brain-inspired computing needs a master plan. *Nature* **604**, 255–260 (2022).

21. R. C. Paolicelli, G. Bolasco, F. Pagani, L. Maggi, M. Scianni, P. Panzanelli, M. Giustetto, T. A. Ferreira, E. Guiducci, L. Dumas, D. Ragozzino, C. T. Gross, Synaptic pruning by microglia is necessary for normal brain development. *Science* **333**, 1456–1458 (2011).

22. T. E. Faust, G. Gunner, D. P. Schafer, Mechanisms governing activity-dependent synaptic pruning in the developing mammalian CNS. *Nat. Rev. Neurosci.* **22**, 657–673 (2021).

23. C. M. Sellgren, J. Gracias, B. Watmuff, J. D. Biag, J. M. Thanos, P. B. Whittredge, T. Fu, K. Worringer, H. E. Brown, J. Wang, A. Kaykas, R. Karmacharya, C. P. Goold, S. D. Sheridan, R. H. Perlis, Increased synapse elimination by microglia in schizophrenia patient-derived models of synaptic pruning. *Nat. Neurosci.* **22**, 374–385 (2019).

24. A. Knoblauch, G. Palm, F. T. Sommer, Memory capacities for synaptic and structural plasticity. *Neural Comput.* **22**, 289–341 (2010).

25. L. Abbott, W. G. Regehr, Synaptic computation. *Nature* **431**, 796–803 (2004).

26. Z. Wang, H. Wu, G. W. Burr, C. S. Hwang, K. L. Wang, Q. Xia, J. J. Yang, Resistive switching materials for information processing. *Nat. Rev. Mat.* **5**, 173–195 (2020).

27. S.-T. Wei, B. Gao, D. Wu, J.-S. Tang, H. Qian, H.-Q. Wu, Trends and challenges in the circuit and macro of RRAM-based computing-in-memory systems. *Chip* **1**, 100004 (2022).

28. B. Chen, F. Cai, J. Zhou, W. Ma, P. Sheridan, W. D. Lu, "Efficient in-memory computing architecture based on crossbar arrays," in *2015 IEEE International Electron Devices Meeting* (IEDM) (IEEE, 2015), pp. 17–5.

29. V. Ramanujan, M. Wortsman, A. Kembhavi, A. Farhadi, M. Rastegari, "What's hidden in a randomly weighted neural network?," in *Proceedings of the IEEE/CVF conference on computer vision and pattern recognition* (IEEE, 2020), pp. 11893–11902.

30. A. Pensia, S. Rajput, A. Nagle, H. Vishwakarma, D. Papailiopoulos, Optimal lottery tickets via subset sum: Logarithmic over-parameterization is sufficient. *Adv. Neural. Inf. Process. Syst.* **33**, 2599–2610 (2020).

31. Y. Li, A. Moitra, T. Geller, P. Panda, "Input-aware dynamic timestep spiking neural networks for efficient in-memory computing," in *2023 60th ACM/IEEE Design Automation Conference (DAC)* (IEEE, 2023), pp. 1–6.

32. Y. Li, T. Geller, Y. Kim, P. Panda, SEENN: Towards temporal spiking early-exit neural networks. *Adv. Neural. Inf. Process. Syst.* **36**, 63327–63342 (2024).

33. C. Li, E. G. Jones, S. Furber, "Unleashing the potential of spiking neural networks with dynamic confidence," in *Proceedings of the IEEE/CVF International Conference on Computer Vision* (IEEE, 2023), pp. 13350–13360.

34. T. Salimans, I. Goodfellow, W. Zaremba, V. Cheung, A. Radford, X. Chen, Improved techniques for training GANS. *Adv. Neural. Inf. Process. Syst.* **29**, 2234–2242 (2016).

35. G. Orchard, A. Jayawant, G. K. Cohen, N. Thakor, Converting static image datasets to spiking neuromorphic datasets using saccades. *Front. Neurosci.* **9**, 159859 (2015).

36. W. Fang, Y. Chen, J. Ding, Z. Yu, T. Masquelier, D. Chen, L. Huang, H. Zhou, G. Li, Y. Tian, SpikingJelly: An open-source machine learning infrastructure platform for spike-based intelligence. *Sci. Adv.* **9**, eadi1480 (2023).

37. L. Van der Maaten, G. Hinton, Visualizing data using t-SNE. *J. Mach. Learn. Res.* **9**, 2579–2605 (2008).

38. Z. Wang, C. Li, P. Lin, M. Rao, Y. Nie, W. Song, Q. Qiu, Y. Li, P. Yan, J. P. Strachan, N. Ge, N. McDonald, Q. Wu, M. Hu, H. Wu, R. Williams, Q. Xia, J. J. Yang, In situ training of feed-forward and recurrent convolutional memristor networks. *Nat. Mach. Intell.* **1**, 434–442 (2019).

39. Q. Xia, J. J. Yang, Memristive crossbar arrays for brain-inspired computing. *Nat. Mater.* **18**, 309–323 (2019).

40. C. Li, M. Hu, Y. Li, H. Jiang, N. Ge, E. Montgomery, J. Zhang, W. Song, N. Dávila, C. E. Graves, Z. Li, J. P. Strachan, P. Lin, Z. Wang, M. Barnell, Q. Wu, R. Williams, J. J. Yang, Q. Xia,

Analogue signal and image processing with large memristor crossbars. *Nat. Electron.* **1**, 52–59 (2018).

41. A. Amir, B. Taba, D. Berg, T. Melano, J. McKinstry, C. Di Nolfo, T. Nayak, A. Andreopoulos, G. Garreau, M. Mendoza, J. Kusnitz, M. Debole, S. Esser, T. Delbruck, M. Flickner, D. Modha, "A low power, fully event-based gesture recognition system," in *Proceedings of the IEEE conference on computer vision and pattern recognition* (IEEE, 2017), pp. 7243–7252.

42. H. Kamata, Y. Mukuta, T. Harada, Fully spiking variational autoencoder. *Proc. AAAI Conf. Artif. Intell.* **36**, 7059–7067 (2022).

43. R. A. Yeh, C. Chen, T. Yian Lim, A. G. Schwing, M. Hasegawa-Johnson, M. N. Do, "Semantic image inpainting with deep generative models," in *Proceedings of the IEEE conference on computer vision and pattern recognition* (IEEE, 2017), pp. 5485–5493.

44. J. Peng, D. Liu, S. Xu, H. Li, "Generating diverse structure for image inpainting with hierarchical VQ-VAE," in *Proceedings of the IEEE/CVF Conference on Computer Vision and Pattern Recognition* (IEEE, 2021), pp. 10775–10784.

45. Y. LeCun, L. Bottou, Y. Bengio, P. Haffner, Gradient-based learning applied to document recognition. *Proc. IEEE* **86**, 2278–2324 (1998).

46. S. Barratt, R. Sharma, A note on the inception score. arXiv:1801.01973 (2018).

47. H. Xiao, K. Rasul, R. Vollgraf, Fashion-MNIST: A novel image dataset for benchmarking machine learning algorithms. arXiv:1708.07747 (2017).

48. P. Lin, C. Li, Z. Wang, Y. Li, H. Jiang, W. Song, M. Rao, Y. Zhuo, N. K. Upadhyay, M. Barnell, W. Qing, J. J. Yang, Q. Xia, Three-dimensional memristor circuits as complex neural networks. *Nat. Electron.* **3**, 225–232 (2020).

49. A. Sebastian, M. Le Gallo, E. Eleftheriou, Computational phase-change memory: Beyond von Neumann computing. *J. Phys. D Appl. Phys.* **52**, 443002 (2019).

50. A. Sebastian, M. Le Gallo, R. Khaddam-Aljameh, E. Eleftheriou, Memory devices and applications for in-memory computing. *Nat. Nanotechnol.* **15**, 529–544 (2020).

51. Y. Wu, L. Deng, G. Li, J. Zhu, Y. Xie, L. Shi, Direct training for spiking neural networks: Faster, larger, better. *Proc. AAAI Conf. Artif. Intell.* 1311–1318 (2019).

52. Y. Bengio, N. Léonard, A. Courville, Estimating or propagating gradients through stochastic neurons for conditional computation. arXiv:1308.3432 (2013).

53. L. Orseau, M. Hutter, O. Rivasplata, Logarithmic pruning is all you need. *Adv. Neural. Inf. Process. Syst.* **33**, 2925–2934 (2020).

54. A. da Cunha, E. Natale, L. Viennot, "Proving the strong lottery ticket hypothesis for convolutional neural networks," in *ICLR 2022-10th International Conference on Learning Representations* (2022).

55. E. Malach, G. Yehudai, S. Shalev-Schwartz, O. Shamir, "Proving the lottery ticket hypothesis: Pruning is all you need," in *International Conference on Machine Learning* (PMLR, 2020), pp. 6682–6691.

56. B. Rueckauer, I.-A. Lungu, Y. Hu, M. Pfeiffer, S.-C. Liu, Conversion of continuous-valued deep networks to efficient event-driven networks for image classification. *Front. Neurosci.* **11**, 682 (2017).

57. J. Ding, Z. Yu, Y. Tian, T. Huang, Optimal ann-snn conversion for fast and accurate inference in deep spiking neural networks. arXiv:2105.11654 (2021).

58. Y. Cao, Y. Chen, D. Khosla, Spiking deep convolutional neural networks for energy-efficient object recognition. *Int. J. Comput. Vis.* **113**, 54–66 (2015).

59. W. Zhang, P. Li, Temporal spike sequence learning via backpropagation for deep spiking neural networks. *Adv. Neural. Inf. Process. Syst.* **33**, 12022–12033 (2020).

60. Y. Zhu, Z. Yu, W. Fang, X. Xie, T. Huang, T. Masquelier, Training spiking neural networks with event-driven backpropagation. *Adv. Neural. Inf. Process. Syst.* **35**, 30528–30541 (2022).

61. A. Levisse, B. Giraud, J.-P. Noel, M. Moreau, J.-M. Portal, "RRAM crossbar arrays for storage class memory applications: Throughput and density considerations," in *2018 Conference on Design of Circuits and Integrated Systems (DCIS)* (IEEE, 2018), pp. 1–6.

62. T. Huynh-Bao, S. Sakhare, J. Ryckaert, A. Spessot, D. Verkest, A. Mocuta, "SRAM designs for 5nm node and beyond: Opportunities and challenges," in *2017 IEEE International Conference on IC Design and Technology (ICICDT)* (IEEE, 2017), pp. 1–4.

63. J. Ko, D. Kwon, J. Hwang, K.-H. Lee, S. Oh, J. Kim, J. Im, R.-H. Koo, J.-J. Kim, J.-H. Lee, SNNSim: Investigation and optimization of large-scale analog spiking neural networks based on flash memory devices. *Adv. Int. Syst.* **6**, 2300456 (2024).

64. A. J. López-Martín, S. Baswa, J. Ramirez-Angulo, R. G. Carvajal, Low-voltage super class AB CMOS OTA cells with very high slew rate and power efficiency. *IEEE J. Solid-State Circuits* **40**, 1068–1077 (2005).

65. G. Gallego, T. Delbrück, G. Orchard, C. Bartolozzi, B. Taba, A. Censi, S. Leutenegger, A. J. Davison, J. Conradt, K. Daniilidis, D. Scaramuzza, Event-based vision: A survey. *IEEE Trans. Pattern Anal. Mach. Intell.* **44**, 154–180 (2020).

66. H. Rebecq, D. Gehrig, D. Scaramuzza, "ESIM: An open event camera simulator," in *Conference on Robot Learning* (PMLR, 2018), pp. 969–982.

67. M. Yao, O. Richter, G. Zhao, N. Qiao, Y. Xing, D. Wang, T. Hu, W. Fang, T. Demirci, M. De Marchi, L. Deng, T. Yan, C. Nielsen, S. Sheik, C. Wu, Y. Tian, B. Xu, G. Li, Spike-based dynamic computing with asynchronous sensing-computing neuromorphic chip. *Nat. Commun.* **15**, 4464 (2024).

68. W. Fang, Z. Yu, Z. Zhou, D. Chen, Y. Chen, Z. Ma, T. Masquelier, Y. Tian, Parallel spiking neurons with high efficiency and ability to learn long-term dependencies. *Adv. Neural. Inf. Process. Syst.* **36**, 53674–53687 (2024).

69. C. Zhou, H. Zhang, L. Yu, Y. Ye, Z. Zhou, L. Huang, Z. Ma, X. Fan, H. Zhou, Y. Tian, Direct training high-performance deep spiking neural networks: A review of theories and methods. *Front. Neurosci.* **18**, 1383844 (2024).

70. Y. Zhu, W. Fang, X. Xie, T. Huang, Z. Yu, Exploring loss functions for time-based training strategy in spiking neural networks. *Adv. Neural. Inf. Process. Syst.* **36**, 65366–65379 (2024).

71. Y. Huang, X. Lin, H. Ren, H. Fu, Y. Zhou, Z. Liu, B. Pan, B. Cheng, Clif: Complementary leaky integrate-and-fire neuron for spiking neural networks. arXiv:2402.04663 (2024).

72. W. Fang, Z. Yu, Y. Chen, T. Huang, T. Masquelier, Y. Tian, Deep residual learning in spiking neural networks. *Adv. Neural. Inf. Process. Syst.* **34**, 21056–21069 (2021).

73. L. Cordone, B. Miramond, S. Ferrante, "Learning from event cameras with sparse spiking convolutional neural networks," in *2021 International Joint Conference on Neural Networks* (IJCNN) (IEEE, 2021), pp. 1–8.

74. J. Tang, J.-H. Lai, L. Yang, X. Xie, "Spike-temporal latent representation for energy-efficient event-to-video reconstruction," in *European Conference on Computer Vision* (Springer, 2025), pp. 163–179.

75. H. Ren, Y. Zhou, Y. Huang, H. Fu, X. Lin, J. Song, B. Cheng, Spikepoint: An efficient point-based spiking neural network for event cameras action recognition. arXiv:2310.07189 (2023).

76. S. A. Tumpa, A. Devulapally, M. Brehove, E. Kyubwa, V. Narayanan, "SNN-ANN hybrid networks for embedded multimodal monocular depth estimation," in *2024 IEEE Computer Society Annual Symposium on VLSI (ISVLSI)* (IEEE, 2024), pp. 198–203.

77. L. Zhu, X. Wang, Y. Chang, J. Li, T. Huang, Y. Tian, "Event-based video reconstruction via potential-assisted spiking neural network," in *Proceedings of the IEEE/CVF Conference on Computer Vision and Pattern Recognition* (IEEE, 2022), pp. 3594–3604.

78. J. Hagenaars, F. Paredes-Vallés, G. De Croon, Self-supervised learning of event-based optical flow with spiking neural networks. *Adv. Neural. Inf. Process. Syst.* **34**, 7167–7179 (2021).

79. B. Yin, F. Corradi, S. M. Bohté, Accurate online training of dynamical spiking neural networks through forward propagation through time. *Nat. Mach. Intell.* **5**, 518–527 (2023).

80. Q. Meng, M. Xiao, S. Yan, Y. Wang, Z. Lin, Z.-Q. Luo, "Towards memory-and time-efficient backpropagation for training spiking neural networks," in *Proceedings of the IEEE/CVF International Conference on Computer Vision* (IEEE, 2023), pp. 6166–6176.

81. M. Xiao, Q. Meng, Z. Zhang, D. He, Z. Lin, Online training through time for spiking neural networks. *Adv. Neural. Inf. Process. Syst.* **35**, 20717–20730 (2022).

82. A. Yousefzadeh, M. A. Khoei, S. Hosseini, P. Holanda, S. Leroux, O. Moreira, J. Tapson, B. Dhoedt, P. Simoens, T. Serrano-Gotarredona, B. Linares-Barranco, Asynchronous spiking neurons, the natural key to exploit temporal sparsity. *IEEE J. Emerg. Sel. Top. Circuits Syst.* **9**, 668–678 (2019).

83. V.-N. Dinh, N.-M. Bui, V.-T. Nguyen, D. John, L.-Y. Lin, Q.-K. Trinh, NUTS-BSNN: A non-uniform time-step binarized spiking neural network with energy-efficient in-memory computing macro. *Neurocomputing* **560**, 126838 (2023).

84. H. Jiang, V. Zoonekynd, G. De Masi, B. Gu, H. Xiong, "TAB: Temporal accumulated batch normalization in spiking neural networks," in *The Twelfth International Conference on Learning Representations* (2024).

85. S. Deng, H. Lin, Y. Li, S. Gu, "Surrogate module learning: Reduce the gradient error accumulation in training spiking neural networks," in *International Conference on Machine Learning* (PMLR, 2023), pp. 7645–7657.

86. Z. Song, P. Katti, O. Simeone, B. Rajendran, Xpikeformer: Hybrid analog-digital hardware acceleration for spiking transformers. arXiv:2408.08794 (2024).

87. M. Abdullah-Al Kaiser, A. R. Jaiswal, "Hardware-algorithm co-design enabling processing-in-pixel-in-memory (P 2 M) for neuromorphic vision sensors," in *ICASSP 2024-2024 IEEE International Conference on Acoustics, Speech and Signal Processing (ICASSP)* (IEEE, 2024), pp. 13356–13360.

88. M. A.-A. Kaiser, G. Datta, Z. Wang, A. P. Jacob, P. A. Beerel, A. R. Jaiswal, Neuromorphic-p2m: Processing-in-pixel-inmemory paradigm for neuromorphic image sensors. *Front. Neuroinform.* **17**, 1144301 (2023).

89. Y. Chen, Z. Yu, W. Fang, T. Huang, Y. Tian, Pruning of deep spiking neural networks through gradient rewiring. arXiv:2105.04916 (2021).

90. H. Huang, L. He, F. Liu, R. Zhao, L. Shi, :Neural dynamics pruning for energy-efficient spiking neural networks," in *2024 IEEE International Conference on Multimedia and Expo (ICME)* (IEEE, 2024), pp. 1–6.

91. L. Deng, Y. Wu, Y. Hu, L. Liang, G. Li, X. Hu, Y. Ding, P. Li, Y. Xie, Comprehensive snn compression using admm optimization and activity regularization. *IEEE Trans. Neural. Netw. Learn. Syst.* **34**, 2791–2805 (2023).

92. B. Han, F. Zhao, W. Pan, Y. Zeng, Adaptive sparse structure development with pruning and regeneration for spiking neural networks. *Inform. Sci.* **689**, 121481 (2025).

93. B. Han, F. Zhao, Y. Zeng, G. Shen, Developmental plasticity-inspired adaptive pruning for deep spiking and artificial neural networks. *IEEE Trans. Pattern Anal. Mach. Intell.* **47**, 240–251 (2025).

94. L. Meng, G. Qiao, X. Zhang, J. Bai, Y. Zuo, P. Zhou, Y. Liu, S. Hu, An efficient pruning and fine-tuning method for deep spiking neural network. *Appl. Intell.* **53**, 28910–28923 (2023).

95. Y. Li, X. Fang, Y. Gao, D. Zhou, J. Shen, J. K. Liu, G. Pan, Q. Xu, Efficient structure slimming for spiking neural networks. *IEEE Trans. Artif. Intell.* **5**, 3823–3831 (2024).

96. H. Cheng, J. Cao, E. Xiao, M. Sun, L. Yang, J. Zhang, X. Lin, B. Kailkhura, K. Xu, R. Xu, Pursing the sparse limitation of spiking deep learning structures. arXiv:2311.12060 (2023).

97. M. Dampfhoffer, T. Mesquida, A. Valentian, L. Anghel, Backpropagation-based learning techniques for deep spiking neural networks: A survey. *IEEE Trans. Neural. Netw. Learn. Syst.* **35**, 11906–11921 (2024).

98. Y. Kim, H. Park, A. Moitra, A. Bhattacharjee, Y. Venkatesha, P. Panda, Rate coding or direct coding: "Which one is better for accurate, robust, and energy-efficient spiking neural networks?," in *ICASSP 2022-2022 IEEE International Conference on Acoustics, Speech and Signal Processing (ICASSP)* (IEEE, 2022), pp. 71–75.

99. S. Ke, F. Tong, Y. Jin, Y. Xiao, J. Meng, S. Chen, Z. Zhang, J. Wang, C. Ye, Highly uniform silver ion memristors with ultralow leakage current for constructing homogeneously spiking LIF neurons. *IEEE Trans. Electron. Devices* **71**, 7911–7915 (2024).

100. W. Guo, M. E. Fouda, A. M. Eltawil, K. N. Salama, Neural coding in spiking neural networks: A comparative study for robust neuromorphic systems. *Front. Neurosci.* **15**, 638474 (2021).

101. D. Arribas, Y. Zhao, I. M. Park, Rescuing neural spike train models from bad MLE. *Adv. Neural. Inf. Process. Syst.* **33**, 2293–2303 (2020).

102. S. D'Agostino, F. Moro, T. Torchet, Y. Demirağ, L. Grenouillet, N. Castellani, G. Indiveri, E. Vianello, M. Payvand, DenRAM: Neuromorphic dendritic architecture with RRAM for efficient temporal processing with delays. *Nat. Commun.* **15**, 3446 (2024).

103. T. Dalgaty, F. Moro, Y. Demirağ, A. De Pra, G. Indiveri, E. Vianello, M. Payvand, Mosaic: In-memory computing and routing for small-world spike-based neuromorphic systems. *Nat. Commun.* **15**, 142 (2024).

104. B. Cramer, S. Billaudelle, S. Kanya, A. Leibfried, A. Grübl, V. Karasenko, C. Pehle, K. Schreiber, Y. Stradmann, J. Weis, J. Schemmel, F. Zenke, Surrogate gradients for analog neuromorphic computing. *Proc. Natl. Acad. Sci. U.S.A.* **119**, e2109194119 (2022).

105. M. P. E. Apolinario, A. K. Kosta, U. Saxena, K. Roy, Hardware/software co-design with adc-less in-memory computing hardware for spiking neural networks. *IEEE Trans. Emerg. Top. Comput.* **12**, 35–47 (2024).

106. M. A. Khoei, A. Yousefzadeh, A. Pourtaherian, O. Moreira, J. Tapson, "Sparnet: Sparse asynchronous neural network execution for energy efficient inference," in *2020 2nd IEEE International Conference on Artificial Intelligence Circuits and Systems (AICAS)* (IEEE, 2020), pp. 256–260.
